# Supplementary material for: Method for the quantitative evaluation of ecosystem services in coastal regions
Source: PeerJ. 2019 Jan 14;6:e6234. doi: 10.7717/peerj.6234 (PMC6336092; doi:10.7717/peerj.6234)
Supplement: Supplemental Information 1 [file peerj-07-6234-s001.docx]

**Supplemental Information for the Calculation of the Coastal Ecosystem Service Index**

In this supplemental information, we describe the calculation processes and data sources used to determine the Coastal Ecosystem services Index (CEI). In the CEI, a service score (*I_i_*) is calculated for each of 12 services (*i*=1 to 12). In addition, a trend score (*T_i_*), pressure and resilience score (*PR_i_*), and sustainability score (*S_i_*) were calculated as part of the calculation process of the service score (*I_i_*). In an evaluation using the CEI, each of these scores can have important implications.

**1. Food provision (*i* = 1)**

**1.1. Setting the index**

In tidal flats, various marine products are caught and provision of food is one of the most popular ecosystem services (Barbier et al., 2011). There are two approaches to evaluating food provision: considering biomass (stock) and considering the quantities caught (flow). With the first method, the system is evaluated by considering the potential capacity (option value) of the ecosystem service; with the second, we evaluate the ecosystem service at the present time. To evaluate the state and sustainability of ecosystem services, it is more direct to use the biomass (i.e., the potential capacity) rather than the catch (Halpern et al., 2015). Therefore, we used the wet weight of commercially important species rather than the catch. We assigned an index value of 1 to the existing quantity (wet weight) of commercially important species. Because the existing quantity of commercially important species is strongly influenced by the size of the tidal flat, the existing quantity per unit area (g-wet/m^2^) was used as the indicator. Furthermore, to eliminate the bias of seasonal variation of biomass to the extent possible, the index (*X*_1_) was averaged annually and calculated as follows:

$X_{1}=\sum_{k=1}^{N} \sum_{l=1}^{M} ({Fish}_{k,l}/M)/N$ (S1)

where ${Fish}_{k,l}$ is the biomass of commercially important species (g-wet/m^2^) in survey *l* at station *k*, *N* is the number of stations, and *M* is the number of surveys per year.

Commercially important species were defined as species targeted for fishing in the intertidal zone and consisted of 12 species (*Anadara broughtonii, Scapharca kagoshimensis, Atrina pectinata, Fulvia mutica, Ruditapes philippinarum, Meretrix lusoria, Tresus keenae, Stichopus japonica Celenka, Mercenaria mercenaria, Corbicula japonica, Solen strictus*, and *Mactra chinensis*) (Japanese Fisheries Agency Marino Forum 21, 2007; Water Environment Committee of the Ministry of the Environment Central Environment Council, 2007; Central Block Council for Fisheries Industry R&D, 2013).

**1.2. Conceptual model**

We created a conceptual model of the environmental factors affecting the appearance of commercially important species (Fig. S1). In Tokyo Bay, deterioration of water quality accompanying eutrophication has been remarkable (Furukawa and Okada, 2006). Anoxic water of the bottom layer occurs 3–4 months a year (Furukawa and Okada, 2006). Sulfide is eluted from the bottom sediments due to the anaerobic condition, and the sulfide sometimes reaches the surface layer via upwelling caused by the wind. The sulfides reaching the surface layer oxidize and turn a pale white color, and this water mass is called blue tide. Because this water mass contains much sulfide, it is highly toxic for aquatic organisms, and organisms on tidal flats with poor migration abilities die in large quantities during a blue tide. In Tokyo Bay, the blue tide occurs about four times a year (Furukawa and Okada, 2006), and it induces death in marine organisms, including commercial species (Uzaki et al., 2003: Toba et al., 2008: Nagasoe et al., 2011). On the other hand, eutrophic conditions in Tokyo Bay supply enough feed to some commercial species, such as clams.

**Figure S1. Conceptual model of environmental factors for food provision**

Other factors in the natural system affect food provision. For example, competing species such as *Musculista senhousia* and predator species such as *Glossaulax didyma* influence the habitat of *Ruditapes philippinarum*, which is major commercial species (Kakino, 1992). In addition, bivalves are inhibited in tidal flats where floating sand, erosion, or subsidence occurs (Kakino, 2000). Furthermore, the supply of larvae from the surrounding habitat is necessary for the reproduction of commercial species (Kakino, 1992).

In terms of the social system, to maintain the production of commercial species, it is important to have groups that manage the fishing area environment. In addition, the management groups also need to conduct species protection activities, such as seedings, control predators and competitors, and manage ground conditions (e.g., by sand capping, cultivation and removing massive *Ulva* sp.).

Considering the above environmental conditions, we set the environmental factors of the food provision as shown in Table S1. Anoxic water, blue tide, predatory or competitive species, primary productivity, stability of ground, and source of juveniles were included in the natural system. Management of the ground and protection of species were included in the social system. Each environmental factor acts both as a pressure and resilience force and will be assigned a PR score as described below.

**Table S1. Environmental factors for food provision**

**1.3. Data collection**

To calculate the wet weight of commercially important species, we collected survey results for benthic organisms for 2009 to 2013 (Ministry of Land, Infrastructure, Transport and Tourism [MLIT], Kanto Regional Development Bureau, Tokyo International Airport Construction Development Office, 2008, 2009a, 2009b, 2010a, 2010b, 2011, 2012, 2013a, 2013b; MLIT, Kanto Regional Development Bureau, Yokohama Port and Airport Technology Investigation Office, 2009, 2011a, 2012a, 2013a, 2014a; Biodiversity Center, Nature Conservation Bureau, Ministry of the Environment, 2010, 2011, 2012, 2013; Yokohama Environmental Science Research Institute, 2010, 2014). The data used included benthic organism survey data for three locations in SN, three locations in UK, four locations in TR, and five locations in OR (Fig. S2).

**Figure S2. Location of survey stations for benthic organisms in four tidal flats: (a) SN, (b) UK, (c) TR, and (d) OR**

As quantitative data for anoxic waters and primary productivity, we collected DO concentration and Chl-a concentration data, respectively, from surrounding waters for 2009 to 2013 (MLIT, Kanto Regional Development Bureau, Tokyo International Airport Construction Office, 2008, 2009a, 2009b, 2010a, 2010b, 2011, 2012, 2013a, 2013b; MLIT, Kanto Regional Development Bureau, Yokohama Port and Airport Technology Investigation Office, 2009, 2011a, 2012a, 2013a, 2014a; Yokohama Environmental Planning Bureau, 2009, 2010, 2011, 2012, 2013; Subcommittee on Monitoring of the Tokyo Bay Renaissance Promotion Conference, 2010, 2011, 2012, 2013, 2014). With respect to the other environmental factors, we conducted interviews with administrators, caretaker organizations, and fishermen.

**1.4. Calculation results**

Mean annual wet weights for commercially important species were calculated by averaging data from a given year. Present status (*x*_1_) was calculated with Equation (2), using the highest mean annual wet weight recorded for the four tidal flats over the most recent 5 years (3323 g/m^2^ in 2010 for SN) as the reference point *X*_1,R_ (Table S2). In addition, the PR scores for each environmental factor at each tidal flat were evaluated (Fig. S3) as part of the calculation of the overall PR score as described in the main text. The trend score (*T*_1_), PR score (*PR*_1_), likely near-term future status (*x*_1,F_) service score (*I*_1_), and sustainability score (*S*_1_) were obtained by using Equations (1)–(4) (Table S3). Please note the index of food provision (*X*_1_) does not represent the annual catch (flow), but the biomass (stock). Therefore, even if the maximum value is used as the reference point, there is no risk of evaluating an overfishing status as optimal.

**Figure S3. Radar chart of PR_1_ scores for each environmental factor in food provision: (a) SN, (b) UK, (c) TR, and (d) OR**

**Table S2. Mean annual wet weights of commercially important species (*X*_1_; g-wet/m^2^) and** **present status (*x*_1_) values for food provision**

**Table S3.** **Present status (*x*_1_),** **trend score (*T*_1_), PR score (*PR*_1_), likely near-term future status (*x*_1,F_), service score (*I*_1_), and sustainability score (*S*_1_) for each tidal flat for food provision**

**2. Coastal protection (*i* = 2)**

**2.1. Setting the index**

Waves from offshore break in front of tidal areas because of their shallow depth. Since the wave energy is reduced when the wave breaks, a tidal flat is expected to have the function of maintaining the coastline. In addition, to protect the area near the coast from waves and storm surges during weather disturbances, seawalls have often been constructed behind tidal flats (on the landward side) in urban areas in Japan. Therefore, we considered both of these functions for the coastal protection service.

As the index of the function of maintaining the coastline during normal periods, we used the reduction ratio of wave energy. For the protection index, we used the wave run-up height ratio of the wave relative to the seawall. Because it is difficult to quantitatively clarify a superiority/inferiority relationship between these two indices, they were weighted equally in the main index:

$x_{2}=W_{2}\times x_{2.1}+(1-W_{2})\times x_{2.2}$ (S2)

where $x_{2}$ is the present status of coastal protection, $x_{2.1}$ is the present status of the wave energy reduction ratio, $x_{2.2}$ is the present status of the wave run-up height ratio, and $W_{2}$ is the weighting coefficient (i.e., 0.5).

The wave energy reduction ratio can be calculated from Equations (S3) and (S4):

$X_{2.1}=(E_{0}-E_{S})/E_{0}\times100$ (S3)

*E*$=\rho gH^{2}/8$ (S4)

where $X_{2.1}$ is the wave energy reduction ratio, *E*_0_ and *E_S_* are offshore and coast wave energies (N/m), *ρ* is seawater density (1025 kg/m^3^), *g* is gravitational acceleration (9.8 m/s^2^), and *H* is the wave height (m).

The offshore wave height *H*_0_ was estimated from wind direction and wind speed data by the SMB method (Bretschneider, 1957). The wave height (*H*) at the coast was calculated from the wave height change equation for shallow water areas (Mase et al., 1986):

$\tilde{H}=H/H_{b}$ (S5)

$\tilde{h}=h/h_{b}$ (S6)

$\tilde{H}^{-4}=\left( 1+4/9K \right)\tilde{h}+4/9K\tilde{h}^{-1/2}$ (S7)

where *H_b_* is the breaking wave height (m), *h* is the depth of water (m), and *h_b_* is the depth of the breaking wave (m). *K* is calculated from Equations (S8)–(S10), tan θ is the seabed gradient, and *L*_0_ is the offshore wave length.

$K=\sqrt{2\gamma/\pi}B\varepsilon^{-1/2}$ (S8)

$\varepsilon=tan\theta/\sqrt{H_{0}/L_{0}}$ (S9)

$\gamma=0.7+5tan\theta$ (S10)

Coefficient *B* is a function of water depth as shown in Equation (S11) (Mase et al., 1986). The breaking wave height (*H_b_*) is calculated from Equation (S12) (Goda, 1970).

$B=\left\{ \begin{aligned} 11-10\tilde{h}, if 0.6<\tilde{h}<1.0 \\ 5, if 0.6\geq\tilde{h} \end{aligned} \right.$ (S11)

$H_{b}/L_{0}=0.17\left\{ 1-exp(-1.5\pi h_{b}/L_{0}(1+15{tan}^{4/3}\theta)) \right\}$ (S12)

The wave run-up height was calculated using Equations (S13) and (S14) (Tamada et al., 2009); it was scored as the ratio of the wave run-up height of the wave to the top height of the seawall (Equation S15).

$\frac{R}{H_{0}}=a+b\exp\left( {cI}_{r,s} \right)$ (S13)

$I_{r,s}=tan \alpha/\sqrt{H_{0}/L_{0}}$ (S14)

$X_{2.2}=(1-R/h_{c})$ (S15)

In these equations, *R* is the wave run-up height (m); *H*_0_ is the offshore wave height (m); *L*_0_ is the offshore wave wavelength (m); *tan α* is the improved virtual gradient (Fig. S4); *I_r,s_* is the Iribarren constant; *a*, *b*, and *c* are constants, and *h* is water depth (m). The constants *a*, *b*, and *c* corresponde to a wave run-up height of R_2%_ for wave numbers of 2% of the incident wave (*a* = 2.99, *b* = –2.73, *c* = –0.57).

**Figure S4. Definition of variables for calculating wave run up height**

**2.2. Conceptual model**

We created a conceptual model of the environmental factors affecting the wave energy reduction ratio and relative wave run-up height (Fig. S5). During normal times, it is important to maintain the intertidal zone shape where wave energy decreases. For this purpose, the ground needs to be stable and little or no ground subsidence, erosion, and floating sand should occur. Ground condition management such as beach nourishment and leveling to maintain the topology are also important. To reduce wave run-up height during weather disturbances, it is important that the height of the top of seawall is maintained, which requires that seawalls be inspected and restored.

**Figure S5. Conceptual model of environmental factors for coastal protection**

Based on the conceptual model, we defined three environmental factors for the coastal protection service. Ground stability was defined as an environmental factor in the natural system, and management of ground condition and inspection and repair were defined in the social system (Table S4).

**Table S4. Environmental factors for coastal protection**

**2.3. Data collection**

To calculate the wave energy reduction and wave run-up height ratios, wind direction and speed data from 2009 to 2013 and topography data for each tidal flat were collected (Japan Oceanographic Data Center; Japan Meteorological Agency; Yokohama Offshore Environmental Conservation Agency, 2000; MLIT, Kanto Regional Development Bureau, Yokohama Port and Airport Technology Investigation Office, 2010, 2011b, 2012b, 2013b, 2014b; Port and Airport Research Institute, unpublished data). Information about the three environmental factors were collected by interviews with administrators, caretaker organizations, and fishermen.

**2.4. Calculation results**

In the four tidal flats, we calculated the distribution of wave energy in the shore-off direction using Equations (S3)–(S10) and found that wave energy was reduced sufficiently in each tidal flat, except for SN (Fig. S6). The wave energy reduction ratio in the past 5 years had almost no annual variation, and the reference point was set at 0.97 at OR in 2013 (Table S5). In addition, the difference in the wave run-up height ratios among four tidal flats was small, ranging from 0.68 to 0.86 (Table S6). The wave run-up height ratio in the past 5 years had almost no annual variation, and the reference point was set 0.88 at UK in 2012 (Table S7). The weighted average rate *W*_2_ was set at 0.5, and the present status *x*_2_ was calculated (Table S8). In addition, the PR score for each environmental factor at each tidal flat was determined (Fig. S7), as were *T*_2_, *x*_2,F_, *I*_2_, and *S*_2_ (Table S9).

**Figure S6. Relationship between distance from shoreline and mean annual wave energy: (a) SN, (b) UK, (c) TR, and (d) OR**

**Figure S7. Radar chart of PR_2_ scores for each environmental factor in coastal protection: (a) SN, (b) UK, (c) TR, and (d) OR**

**Table S5. Mean annual wave energy reduction ratio (*X*_2.1_) and present status (*x*_2.1_) values for coastal protection**

**Table S6. Calculation results for annual maximum wave run-up height ratio (*X*_2.2_) in 2013**

**Table S7. Annual maximum wave run-up height ratio (*X*_2.2_) and present status (*x*_2.2_) values for coastal protection**

**Table S8. Present status (*x*_2_) values for coastal protection**

**Table S9. Present status (*x*_2_), trend score (*T*_2_), PR score (*PR*_2_), likely near-term future status (*x*_2,F_), service score (*I*_2_), and sustainability score (*S*_2_) for each tidal flat for coastal protection**

**3. Recreation: water front use (*i* = 3)**

**3.1. Setting the index**

Tidal flats are places that offer many recreational opportunities (i.e., cultural services) such as shell gathering, swimming, and leisure fishing (Garcia Rodrigues et al., 2017). In Japan, shell gathering is particularly popular. Many people visit tidal flats for shell gathering from spring to summer. In some tidal flats, there are tens of thousands of visitors per day participating in this activity. We used the number of visitors who came for the purpose of recreation per year as an index of recreation. Because that number is strongly influenced by the size of the tidal flat, the index used visits per unit area as shown in Equation (S16):

$X_{3}=Rec/A$ (S16)

where *Rec* represents the number of visitors to the tidal flat per year (people/y) and *A* represents the area (m^2^).

**3.2. Conceptual model**

We created a conceptual model of the environmental factors affecting the number of visitors for the purpose of recreation (Fig. S8). In terms of natural systems, for recreation use, a health natural system is important as well as the absence of mass die-offs, the bad odor accompanying blue tide, and the outbreak of specific species (*Ulva* sp.). In addition, to maintain good seascapes, erosion or subsidence need to be avoided. Because recreation is a human activity, social factors have a great influence. Therefore, infrastructure such as rest huts, public toilets, event rooms, and wash facilities are needed. Publicity works such as public relations via the web and distribution of advertisements to attract visitors for a variety of activities are also needed. Moreover, to maintain a good seascape, a management organization is needed to conduct and supervise cleaning activities and other facility maintenance. Finally, accessibility is an important factor for visitors, so access to parking or a nearby public transportation station is important.

**Figure S8. Conceptual model of environmental factors for recreation**

From the conceptual model in recreation, seven environmental factors were set (Table S10). Two were in the natural system (healthy habitat and stability of ground) and five were in the social system, two of which were related to infrastructure (accessibility and incidental facilities) and three were related to management (management groups, attracting visitors, and publicity work).

**Table S10. Environmental factors for recreation**

**3.3. Data collection**

The number of visitors for recreation was collected from surveys by the government and other local administrators (Chiba Prefecture Commerce and Industry Labor Tourism Planning Division, 2010, 2011, 2012, 2013, 2014; Kanagawa Prefecture Tourism Promotion Council, 2010, 2012, 2012, 2013, 2014; MLIT, River Bureau). Because all of the environmental factors are qualitative, information about them was collected through interviews with administrators, caretaker organizations, and fishermen. SN is a demonstration facility and is not open to the public, so it is not used for recreation. We therefore omitted it from the evaluation process for this service.

**3.4. Calculation results**

The number of visitors per unit area (*X*_3_) was calculated from the survey data (Table S11). The survey frequency was different for each tidal flat because the survey administrator was different for each tidal flat. The present status (*x*_3_) was obtained with the reference point *X*_3,R_, which was 2220 people/m^2^/y at UK in 2009 (Table S11). In addition, the PR scores for each environmental factor at each tidal flat were evaluated (Fig. S9), and *T*_3_, *PR*_3_, *x*_3,F_, *I*_3_, and *S*_3_ were obtained by using Equations (1)–(4) (Table S12).

**Figure S9. Radar chart of PR_3_ scores for each environmental factor in recreation: (a) UK, (b) TR, and (c) OR. SN was excluded because recreation is not allowed in this area.**

**Table S11. Annual number of visitors for the purpose of recreation (*X*_3_; people/m^2^/y) and present status (*x*_3_) values for recreation**

**Table S12. Present status (*x*_3_), trend score (*T*_3_), PR score (*PR*_3_), likely near-term future status (*x*_3,F_), service score (*I*_3_), and sustainability score (*S*_3_) for each tidal flat for recreation**

**4. Environmental education: water front use (*i* = 4)**

**4.1. Setting the indicators**

Environmental education activities have been implemented as a part of educational activities since the 1950s in Japan (Ogawa, 2009), and these activities can be defined as a different service than other cultural services (e.g., recreation) (Millennium Ecosystem Assessment, 2005). Tidal flats are places where many organisms live and people can interact with the ocean and are thus one of the best places for environmental education activities in Japan. The index was defined as the number of people who visited for the purpose of an environmental education activity. In general, the number of visitors per one environmental education activity was limited to about 50 people at most. Therefore, tidal flat size was not considered to be a strong constraint of the number of visitors for this purpose, and the total number of annual visitors was used as the index without adjustment for area.

$X_{4}=Edu$ (S17)

where *Edu* indicates the number of annual visitors for the purpose of environmental education on the tidal flat (people/y).

**4.2. Conceptual model**

We created a conceptual model of the environmental factors that influence the number of visitors for environmental education activities (Fig. S10). To maintain or increase the number of visitors, similar to recreation, the natural system should be healthy and a good seascape and biodiversity must be maintained. In the social system, factors similar to recreation are also important for environmental education for the same reasons. In addition, it is also important that species protection activities to maintain biodiversity and educational activities are conducted.

**Figure S10. Conceptual model of environmental factors for environmental education**

Based on the conceptual model, nine environmental factors of environmental education were established (Table S13). Three of these were in the natural system (healthy habitat, diversity of creatures, and stability of ground). There were six in the social system, of which accessibility and incidental facilities were related to infrastructure and management groups, attracting visitors, publicity work, and protection of species were related to maintenance and management.

**Table S13. Environmental factors for environmental education**

**4.3. Data collection**

The number of visitors for the purpose of environmental education activities was estimated from interviews with facility administrators, caretaker organizations, and fishermen. We asked about the average number of visitors per activity and the number of activities implemented per year. Using these numbers, we estimated the number of visitors per year.

Data needed for calculating the diversity index (see Section 11.1) for the diversity of creatures were obtained from public benthos research for 2009 to 2013 (see Section 1.3). For the other environmental factors, we conducted interviews with administrators, caretaker organizations, and fishermen.

**4.4. Calculation results**

For SN, we were able to calculate the number of visitors for the purpose of environmental education activities every year from the visitor records. For the other sites, which do not record the number of visitors every year, the number of visitors for this purpose in 2013 was estimated from the hearing (Table S14). The maximum value of the past 5 years, 331 people/y in SN in 2009, was taken as the reference point *X*_4,R_, and the present status (*x*_4_) was calculated. In addition, the PR score for each environmental factor at each tidal flat was calculated (Fig. S11), as were *T*_4_, *PR*_4_, *x*_4,F_, *I*_4_, and *S*_4_ (Table S15).

**Figure S11. Radar chart of PR_4_ scores for each environmental factor in environmental education: (a) SN, (b) UK, (c) TR, and (d) OR**

**Table S14. Annual number of visitors for the purpose of environmental education (*X*_4_; people/y) and present status (*x*_4_) values for environmental education**

**Table S15. Present status (*x*_4_), trend score (*T*_4_), PR score (*PR*_4_), likely near-term future status (*x*_4,F_), service score (*I*_4_), and sustainability score (*S*_4_) for each tidal flat for environmental education**

**5. Research: water front use (*i* = 5)**

**5.1. Setting the indicators**

Tidal flats are an attractive research subject for researchers and have various characteristic physical, chemical, biological, and social phenomena. The number of published papers and reports related to each tidal flat was used as an index of research. Because the number of publications is not considered to be affected by the size of the tidal flat, the index was not corrected for area.

$X_{5}=Stu$ (S18)

where *Stu* shows the number of papers and reports published per year related to each tidal flat.

**5.2. Conceptual model**

Motivation for research is very diverse, and any phenomenon in a tidal flat can be the subject of research. For example, ecosystem dynamics in natural tidal flats is an interesting theme. On the other hand, in artificial tidal flats in an urban region, the ecosystem response to human activity is of interest. For this reason, it was difficult to define specific environmental factors for research. Therefore, we did not create a conceptual model of the environmental factors affecting research.

**5.3. Data collection**

The number of papers and reports from 2009 to 2013 was collected by using a web search and interviews. We used four web search engines, Google Scholar, CiNi, J-Stage, and Agri-Knowledge, and set three keywords for each tidal flat (Table S16). In addition, we conducted interviews with members of research institutions, administrators, and nonprofit organizations related to tidal flats.

**Table S16. Keywords used for web search in research**

**5.4. Calculation results**

The number of papers and reports per year (*X*_5_) is shown in Table S17. The present status *x*_5_ was obtained with the reference point *X*_5,R_, which was 14 papers/y at OR in 2012 (Table S18). In addition, *T*_5_, *x*_5,F_, *I*_5_, and *S*_5_ were obtained by using Equations (1)–(4).

**Table S17. Annual number of papers (*X*_5_; papers/y) and present status (*x*_5_) values for research**

**Table S18. Present status (*x*_5_), trend score (*T*_5_), PR score (*PR*_5_), likely near-term future status (*x*_5,F_), service score (*I*_5_), and sustainability score (*S*_5_) for each tidal flat for research**

**6. Historical designation as special sites: sense of place (*i* = 6)**

**6.1. Setting the indicators**

The ocean has been recognized as a symbol or object of faith since ancient times in Japan, and various rites and festivals have traditionally been held in coastal zones. In addition, buildings related to faith such as *torii*, a traditional Japanese gate most commonly found at the entrance of or within a Shinto shrine, have been built in coastal areas (Akiyama et al., 2017). In the historical designation as special sites, the annual number of rites and festivals and the number of faith-related buildings were used as indices. Because these indices are not considered to be affected by the scale of the tidal flats, we did not correct for area in the indicator. The following equations were used for calculations:

$x_{6}=W_{6}\times x_{6.1}+(1-W_{6})\times x_{6.2}$ (S19)

$X_{6.1}=Fes$ (S20)

$X_{6.2}=Str$ (S21)

where $x_{6}$ is the present status of the historical designation as special sites. $W_{6}$ is the weighting coefficient between *x*_6.1_ and *x*_6.2_, which was set at 1:1 because it is difficult to clarify quantitatively the difference between the two indices, where $x_{6.1}$ is the present status of the annual number of rites and festivals and $x_{6.2}$ is the present status of the number of faith-related buildings, which are obtained from Equation (2) using $X_{6.1}$ and $X_{6.2}$, respectively. *Fes* is the annual number of rites and festivals, and *Str* is the number of faith-related buildings.

**6.2. Conceptual model**

We created a conceptual model of environmental factors that affect the number of rites and festivals and the number of faith-related buildings (Fig. S12). To maintain rites/festivals and faith-related buildings, it is very important to secure successors who will be responsible for maintaining these activities and facilities. In many regions in Japan, festivals in coastal areas are directly or indirectly linked with fishing-related prosperity. In addition, in order for people to have a sense of reverence for the nature, the seascape must be maintained, which requires some sort of management organization that conducts cleaning activities and facility maintenance. Furthermore, similar to recreation and environmental education, the natural system needs to be healthy and the ground has to be stable. Management of the ground condition, such as beach nourishment and leveling, are also important to maintain the tidal flat.

**Figure S12. Conceptual model of environmental factors for historical designation as special sites**

Based on conceptual model, five environmental factors were established for historical designation as special sites (Table S19). Fishery, management groups, and management of ground conditions were defined as environmental factors in the social system and healthy habitat and stability of ground were defined in the natural system.

**Table S19. Environmental factors for historical designation as special sites**

**6.3. Data collection**

Numbers of rites/festivals and faith-related buildings were collected from interviews with facility managers, nonprofit organizations, neighboring residents, and published sources. Because SN and UK are artificial tidal flats, religious activities are prohibited. Therefore, they were excluded from valuation in historical designation as special sites.

**6.4. Calculation results**

In TR, rites and festivals continuing from the Edo era (1603–1868) are held twice a year, in May and January, to wish for a bountiful catch and the safety of the fishery. In OR, a religious ceremony is held on 7 January every year, in which a local young man walks off the tidal flat and then a ritual is held offshore (Kisarazu City, 2012). Currently, these festivals and ceremonies are managed by local fishermen. In addition, there is a *torii* in OR (Figure S13). From these results, the reference point of the annual number of rites and festivals *X*_6.1,R_ was set at 2 (Table S20). The reference point for the number of faith-related buildings *X*_6.2,R_ was set at 1. In addition, the PR score for each environmental factor at each tidal flat was evaluated (Fig. S14), and *x*_6_, *T*_6_, *PR*_6_, *x*_6,F_, *I*_6_ , and *S*_6_ were obtained by using Equations (1)–(4) (Table S21).

**Figure S13. Photo of a *torii* (a traditional Shinto gate) in OR**

**Figure S14. Radar chart of PR_6_ scores for each environmental factor in historical designation as special sites: (a) SN, (b) UK, (c) TR, and (d) OR. SN and UK were excluded because these types of activities and structures are not permitted in these areas.**

**Table S20. Annual number of number of rites and festivals (*X*_6.1_), number of faith-related buildings (*X*_6.2_), and present status (*x*_6_) values for historical designation as special sites**

**Table S21. Present status (*x*_6_), trend score (*T*_6_), PR score (*PR*_6_), likely near-term future status (*x*_6,F_), service score (*I*_6_), and sustainability score (*S*_6_) for each tidal flat historical designation as special sites**

**7. Places for everyday rest and relaxation: sense of place (*i* = 7)**

**7.1. Setting the indicators**

The tidal flat provides attractive amenities for people, and people obtain mental relief or enjoyment from them. These values are defined by a sense of place or aesthetics and are a different kind of value from the dynamic activity and enjoyment obtained from recreation (Millennium Ecosystem Assessment.2005; Halpern et al, 2012; Garcia Rodrigues et al., 2017). In this study, we defined these values as places for everyday rest and relaxation in association with the specific behavior of visitors to tidal flats. The index was set as the total duration of everyday (routine) use, which was defined as people resting, walking, and running at the beach and tidal flat area. In addition, we adjusted the total duration of stay by the visitors’ awareness of the importance of the tidal flat to obtain the index (*X*_7_). The following equation was used for calculations:

$X_{7}= V\times{(\sum_{k=1}^{N} a_{k}\times w_{k}\times h_{k}\times r_{k}}/N)$ (S22)

where *V* is the number of routine users per day (people/d), *k* is the identify number of questionnaire respondents, *N* is the number of respondents in the questionnaire described below, *a_k_* is a dummy variable that distinguishes respondents (everyday users=1, others=0), $w_{k}$ is a variable that distinguishes a respondent who recognized the existence of the tidal flat (recognize=1, not=0), *h_k_* is the duration of stay in the tidal flat (hour), and *r_k_* is the weighting coefficient of awareness of tidal flat. *r_k_* was obtained from answers to the question, “Is this tidal flat important for your community?”: very important=1, important=0.8, rather important=0.6, rather unimportant = 0.4, not important = 0.2, and unnecessary = 0.0. Data for these variables were obtained from an on-site survey described later.

**7.2. Conceptual model**

We created a conceptual model of environmental factors that affect the total duration of stay by everyday users and their awareness of the importance of the tidal flat (Fig. S15). To function as places for everyday rest and relaxation, at tidal flat must be healthy and provide amenities. Therefore, as with recreation, the natural system must be healthy and the ground be stable. In the social system, infrastructure such as rest huts, public toilets, and wash facilities are also needed. In addition, a management organization is needed to ensure that cleaning activities are conducted and that ground condition maintenance, such as sand capping, cultivation, and removal of massive *Ulva* sp., is conducted.

**Figure S15. Conceptual model of environmental factors for places for rest and relaxation**

Based on the conceptual model, five environmental factors of places for everyday rest and relaxation were considered (Table S22), healthy habitat and stability of ground in the natural system and incidental facilities, management groups, and management of ground condition in the social system.

**Table S22. Environmental factors for places for everyday rest and relaxation**

**7.3. Data collection**

Data for this service could not be obtained from existing monitoring or statistical data. Therefore, we conducted a field survey in each tidal flat except SN (which is not open to the public). SN was treated as not subject to valuation in places for everyday rest and relaxation.

We set survey stations for counting the number of visitors and conducting questionnaire surveys (Fig. S16). The field survey was conducted on 20 November 2014 at OR and on 21 November 2014 at UK and TR. The survey was conducted on a weekday from sunrise to sunset to collect data on routine use. We counted the number of visitors to each tidal flat at the survey stations. Use was classified and recorded from appearance in the categories of walking/resting, running, bicycling, fishing, shell gathering, and other recreation. The number of everyday users (*V*) was defined as the sum of the number of users engaged in walking/resting and running.

**Figure S16. Location of survey stations for field survey in three tidal flats: (a) UK, (b) TR, and (c) OR**

We also conducted a questionnaire survey for visitors on the same days. The questionnaire survey was conducted face to face with randomly selected visitors. We asked about personal information (gender, age, distance from place of residence, how they arrived), information on the use and awareness of tidal flats (purpose for visit, duration of stay, and recognition and awareness of the tidal flat). The number of respondents to the questionnaire and the percentage of respondents to the total number of visitors was 81 people (7.3%) in UK, 11 people (40.7%) in TR, and 14 people (37.8%) in OR. Although we only evaluated survey results from one day at each location, we plan to conduct additional similar surveys in different seasons to improve the evaluation’s accuracy. In addition to the survey data, those for the environmental factors were collected from interviews with administrators, caretaker organizations, and fishermen.

**7.4. Calculation results**

There were 1,098 visitors to UK on the survey day, of which 836 visitors were classified as everyday users (Fig. S17). In TR, there were 94 visitors, but 71% of those only biked through the area on a sidewalk, so the number of everyday users was 17 people. In OR, there were 76 visitors, of which 36 people were everyday users. The percentage of people who recognized the existence of tidal flats was more than 90% in all tidal flats (Fig. S18a). The duration of the stay at UK and OR was less than 30 minutes to 1 hour for more than 60% of respondents (Fig. S18b). In contrast, at TR where many users are fishing, more than 60% of respondents stayed for more than 1 hour. Most respondents stated that tidal flats were very important, important, or rather important at all three locations (Fig. S18c).

**Figure S17. Number of visitors per day by usage type**

**Figure S18. Results of questionnaire survey: (a) recognition of the existence of the tidal flat, (b) duration of stay (h), and (c) awareness of the importance of the tidal flat**

Based on the survey results, we used the number of routine users per day and total duration of stay adjusted by awareness of the importance of the tidal flat to calculate *X*_7_ (Table S23). The maximum value of 522 at the UK was set as reference point *X*_7,R_, and the present status (*x*_7_) was obtained by using Equation (2) (Table S23). In addition, the PR score for each environmental factor at each tidal flat was evaluated (Fig. S19), and *T*_7_, *PR*_7_, *x*_7,F_, *I*_7_, and *S*_7_ were obtained by using Equations (1)–(4) (Table S24).

**Figure S19. Radar chart of PR_7_ scores for each environmental factor in places for rest and relaxation: (a) UK, (b) TR, and (c) OR. SN was excluded because these activities are not permitted in this area.**

**Table S23. Number of everyday users per day (*V*, people/day), total duration of everyday use adjusted by an awareness factor (*X*_7_) and present status (*x*_7_) values for places for everyday rest and relaxation**

**Table S24. Present status (*x*_7_), trend score (*T*_7_), PR score (*PR*_7_), likely near-term future status (*x*_7,F_), service score (*I*_7_), and sustainability score (*S*_7_) for each tidal flat for everyday rest and relaxation**

**8. Suspended material removal: water quality (*i* = 8)**

**8.1. Setting the indicators**

In tidal flats, filter feeders such as bivalves remove suspended materials such as phytoplankton from sea water via predation activities (Prins and Smal, 1994; Hosokawa et al., 1996; Magni et al., 2000; Kohata et al., 2003). This is a very important service for maintaining transparency and water quality in coastal areas, and in suspended material removal, the amount of water filtered by bivalves was used as the index. The volume of water filtration by bivalves can be obtained from the following equation (Hosokawa et al., 1996):

$Q=0.085\times{Tw}^{1.25}\times W^{-0.78}$ (S23)

where *Q* is the filtration volume (L/h/g-wet), *Tw* is the water temperature (°C), and *W* is the individual wet weight of bivalves (g-wet).

Bivalve water filtration volume strongly depends on the scale of the tidal flats, so the bivalve water filtration volume per unit area ($X_{8}$) was obtained from the average wet weight per bivalve and the average number of individuals at each survey station as follows:

$X_{8}= \sum_{k=1}^{N} \sum_{l=1}^{M} (Q_{k,l}/M)/N$ (S24)

$Q_{k,l}= 0.085\times{{wt}_{k,l}}^{1.25}\times\left( W_{k,l} \right)^{-0.78}\times P_{k.l}$ (S25)

where $Q_{k,l}$ is the amount of filtered water per unit area (L/m^2^/h) in survey *l* at station *k*, *N* is the number of stations, and *M* is the number of surveys per year, ${wt}_{k.l}$ is the water temperature (°C), $W_{k,l}$ is the average weight of a bivalve (g-wet), and $P_{k.l}$ is the number of bivalves per unit area (individuals/m^2^).

**8.2. Conceptual model**

We created a conceptual model of the environmental factors affecting the water filtration volume of bivalves (Fig. S20). A stable habitat and appropriate water environment are important to maintain or increase the abundance of bivalves. Therefore, as is the case with food provision, damage to the ecosystem stemming from anoxic waters and blue tides needs to be avoided (Uzaki et al., 2003: Toba et al., 2008: Nagasoe et al., 2011). Sufficient primary production and a source of larvae in the surrounding area are also important (Kakino, 1992). In addition, the ground should be stable and there should be no floating sand, erosion, and subsidence. In the social system, ground condition management is important, including sand capping, cultivation, and removal of massive *Ulva* sp.

**Figure S20. Conceptual model of environmental factors for suspended material removal**

Based on the results of the conceptual model, six environmental factors were considered for suspended material removal (Table S25), five in the natural system (anoxic waters, blue tide, primary productivity, ground stability, and source of juveniles) and one in the social system (management of ground condition).

**Table S25. Environmental factors for suspended material removal**

**8.3. Data collection**

Benthic organism survey data from 2009 to 2013 were collected from public survey data (MLIT, Kanto Regional Development Bureau, Tokyo International Airport Construction Development Office, 2008, 2009a, 2009b, 2010a, 2010b, 2011, 2012, 2013a, 2013b; MLIT, Kanto Regional Development Bureau, Yokohama Port and Airport Technology Investigation Office, 2009, 2011a, 2012a, 2013a, 2014a; Biodiversity Center, Nature Conservation Bureau, Ministry of the Environment, 2010, 2011, 2012, 2013;Yokohama Environmental Science Research Institute, 2010, 2014; Chiba Prefectural Fisheries Research Center, unpublished data). The data used included survey data for three locations in SN, 10 locations in UK, four locations in TR, and one location in OR (Fig. S21).

**Figure S21. Location of survey stations for bivalves in four tidal flats: (a) SN, (b) UK, (c) TR, and (d) OR**

Quantitative data were used for anoxic waters and primary productivity; we collected DO concentration data and Chl-*a* concentration data, respectively, from surrounding waters from 2009 to 2013 (see Section 1.3). For the other environmental factors, we conducted interviews with administrators, caretaker organizations, and fishermen.

**8.4. Calculation results**

The mean annual amounts of filtered water were calculated with Equations (S23)–(S25). Present status (*x*_8_) was calculated with Equation (2), using the highest mean annual amount of filtered water over the most recent 5 years, 13.7 m^3^/m^2^/h in 2010 for SN, as the reference point *X*_8,R_ (Table S26). In addition, the PR scores for each environmental factor at each tidal flat were evaluated (Fig. S22), and *T*_8_, *PR*_8_, *x*_8,F_, *I*_8_, and *S*_8_ were obtained by using Equations (1)–(4) (Table S27).

**Figure S22. Radar chart of PR_8_ scores for each environmental factor in suspended material removal: (a) SN, (b) UK, (c) TR, and (d) OR**

**Table S26. Annual mean of bivalve water filtration volume (*X*_8_; m^3^/m^2^/h) and present status (*x*_8_) values for suspended material removal**

**Table S27. Present status (*x*_8_), trend score (*T*_8_), PR score (*PR*_8_), likely near-term future status (*x*_8,F_), service score (*I*_8_), and sustainability score (*S*_8_) for each tidal flat for suspended material removal**

**9. Organic matter decomposition: water quality use (*i* = 9)**

**9.1. Setting the indicators**

Organic matter is consumed and decomposed into nutrients as a result of the activities of consumers and decomposers. Nutrients are used by primary producers, such as benthic microalgae, and transferred to consumers (Aller, 1982; Prins and Smaal, 1994; Epstein, 1997; Kohata et al., 2003). Furthermore, because of the unique physical environment in a tidal flat with its repeating ebb and flow tides, a redox boundary is easy to develop and denitrification occurs (Kaspar, 1983). Therefore, a tidal flat has essentially the same service as a sewage treatment plant. Here we focused on the process of organic matter purification by benthic organisms and defined the service of organic matter decomposition. Organic matter ingested by organisms is divided into parts that are assimilated as body tissues and parts that are excreted by metabolism (Ursin, 1967; Goulletquer & Bacher, 1988; Padres et al., 2000). The purification process was defined as the assimilation part of this process. The amount of organic matter assimilated by benthic organisms was estimated using the P/B (production/abundance) ratio, which was converted into the chemical oxygen demand (COD) purification amount. In addition, because the COD purification amount strongly depends on the area, the COD purification amount per unit area was used as the index, *X*_9_:

$X_{9}= \sum_{k=1}^{N} {OMD}_{k}/N$ (S26)

${OMD}_{k}= \sum_{s=1}^{S} {\gamma_{s}\times B}_{s}\times{P/B}_{s}$ (S27)

where ${OMD}_{k}$ is the COD purification amount per unit area (g-COD/m^2^/y) at the station *k*, *N* is the number of stations, and *S* is the total number of species, $\gamma_{s}$ is the conversion coefficient from the wet weight of species *s* to the COD (g-COD/g-wet), $B_{s}$ is the annual average wet weight (g-wet/m^2^) of species *s*, and ${P/B}_{s}$ is the P/B ratio of species *s*. Because there is not enough information to determine $\gamma_{s}$ and ${P/B}_{s}$ in these tidal flats, we set these coefficients for each phylum by referring to past research (Schwinghamer et al., 1986; Miura et al., 2013) (Table S28).

**Table S28. Production-to-biomass ratio and conversion coefficient (**$\gamma_{s})$**from the wet weight to COD (g-COD/g-wet) for each phylum**

As mentioned above, there are many processes related to purification in tidal flats, including decomposition of organic substances and denitrification by microorganisms. However, there is currently not enough information concerning these other purification processes; therefore, they were not considered as subjects for evaluation in this study. We recognize this as a future task to improve the index.

**9.2. Conceptual model**

In Japanese tidal flats, bivalves often make up most of the benthic organism biomass. Therefore, the environmental factors affecting the biomass of benthic organisms can be regarded as equivalent to those affecting bivalve biomass. For this reason, we assumed that organic matter decomposition has the same environmental factors as suspended matter removal (see Section 8.2). Therefore, the conceptual model of organic matter decomposition was defined using the same factors as those used for suspended material removal (Fig. S23, Table S29).

**Figure S23. Conceptual model of environmental factors for organic matter decomposition**

**Table S29. Environmental factors for organic matter decomposition**

**9.3. Data collection**

To calculate the wet weight of benthic organisms, we collected the necessary survey results for benthic organisms for 2009 to 2013 (see Section 1.3). We used quantitative data for anoxic waters and primary productivity; we collected DO concentration data and Chl-*a* concentration data, respectively, from surrounding waters for 2009 to 2013 (see Section 1.3). With respect to the other environmental factors, we conducted interviews with administrators, caretaker organizations, and fishermen.

**9.4. Calculation results**

The annual COD purification amount was calculated from the wet weight of benthic organisms. The present status (*x*_9_) was calculated with Equation (2), using the maximum annual COD purification amount over the most recent 5 years, 214 g-COD/m^2^/y in 2010 for SN, as reference point *X*_9,R_ (Table S30). In addition, the PR scores for each environmental factor at each tidal flat were evaluated (Fig. S24), and *T*_9_, *PR*_9_, *x*_9,F_, *I*_9_, and *S*_9_ were obtained by using Equations (1)–(4) (Table S31).

**Figure S24. Radar chart of PR_8_ scores for each environmental factor in organic matter decomposition: (a) SN, (b) UK, (c) TR, and (d) OR**

**Table S30. Annual COD purification amount by benthic organisms (*X*_9_; g-COD/m^2^/y) and present status (*x*_9_) values for organic matter decomposition**

**Table S31. Present status (*x*_9_), trend score (*T*_9_), PR score (*PR*_9_), likely near-term future status (*x*_9,F_), service score (*I*_9_), and sustainability score (*S*_9_) for each tidal flat for organic matter decomposition**

**10. Carbon storage (*i* = 10)**

**10.1. Setting the indicators**

The high biological activity in coastal areas is conducive to carbon storage. In particular, mangroves, tidal marshes, and seagrasses are defined as blue carbon, and it was estimated that the amount of CO_2_ absorption as blue carbon is from 50% to 71% of the total CO_2_ absorption by all oceans (Nellemann et al., 2009). The absorption of CO_2_ in tidal flats is largely influenced by environment factors, and both sources and sinks have been reported (Migné et al., 2002, 2004; Spilmont et al., 2005; Klaassen and Spilmont, 2012). Current knowledge concerning carbon storage in tidal flats is not sufficient to use carbon storage flux as an index. Therefore, in this study, we set the amount of carbon fixation in a tidal flat as the indicator. In other words, we considered that the relative carbon storage function can be evaluated from the carbon fixation amount. We recognize that scores for carbon storage are based on a relative comparison between tidal flats, and they do not guarantee a contribution to the mitigation of global warming or climate change. Developing an evaluation method for carbon storage flux remains as a future task.

The carbon fixation amount in a tidal flat was defined as the sum of the amounts of benthic organism carbon fixation and sediment carbon fixation. Both of these fixation amounts show seasonal variations, so to avoid an overestimation, the annual minimum value was used as an index. In addition, because both of these amounts strongly depend on area, the carbon fixation amount per unit area was used as the index:

$X_{10}= min(C_{ben,l}+C_{sed,l})$ (S28)

where $X_{10}$ is carbon fixation amount per unit area (g-C/m^2^), $C_{ben,l}$ is benthic organism carbon fixation amount in survey *l* (g-C/m^2^), and $C_{sed,l}$ is sediment carbon fixation amount (g-C/m^2^). $C_{ben,l}$ and $C_{sed,l}$ are defined by the following equations:

$C_{ben,l}= \sum_{d=1}^{4} {c_{d}\times b}_{d,l}$ (S29)

$C_{sed,l}= {DW}_{sed,l}\times{TOC}_{LOI,l}\times{10}^{-2}$ (S30)

${TOC}_{LOI,l}= {(LOI}_{l}\times0.4)-0.21$ (S31)

where $c_{d}$ is carbon content of the organism at phylum *d* (g-C/g-wet), $b_{d,l}$ is wet weight per unit area of phylum *d* in survey *l* (g-wet/m^2^), ${DW}_{sed,l}$ is dry weight of sediment from the surface layer to a depth of 0.1 m (g-dry/m^2^), ${TOC}_{LOI,l}$ is TOC content per gram dry mud (g-C/g-dry), and ${LOI}_{l}$ is ignition loss (%). The carbon content of each phylum ($c_{d})$was set according to a study in tidal flat areas in Japan as shown in Table S32. Equation (S31), which is a conversion equation from ignition loss to TOC content, is from Fourqurean et al. (2012). The evaluation range of the sediment carbon fixation amount was defined as 0.1 m because the sampling depth in most of the survey data was 0.1 m.

**Table S32. Carbon contents for each phylum (Miura et al., 2013)**

**10.2. Conceptual model**

The influences of environmental factors on the amounts of benthic organism carbon fixation and sediment carbon fixation differ greatly; therefore, we created separate conceptual models for each type of carbon fixation (Figs. S25, S26). Carbon fixation by benthic organisms increases with increasing benthic biomass, so the same index used for organic matter decomposition applies here, and the environmental factors are also the same (see Section 9.2). The sediment carbon fixation amount increases through processes that create organic loads to sediment. Therefore, eutrophication and the deaths of organisms associated with anoxic waters and blue tide are resilience factors. In addition, it is also important to avoid floating sand and erosion so that organic matter can be buried in the bottom mud. In the social system, the countermeasures related to sediment are the main environmental factor, but their influence on sediment carbon fixation depends on the type of countermeasure. Countermeasures that keep organic matter in the sediment (e.g., sand capping) are resilience factors, but those that make it easier to transport sediment organic matter out of the bottom mud (e.g., cultivation) are pressure factors.

**Figure S25. Conceptual model of environmental factors for carbon storage in benthic organisms**

**Figure S26. Conceptual model of environmental factors for carbon storage in sediment**

The environmental factors for the benthic organism carbon fixation amount were the same as those for suspended material removal and organic matter decomposition (Table S33). For the sediment carbon fixation amount, four environmental factors were evaluated: death of organisms, organic load, and ground stability in the natural system, and embedding organic matter in the social system (Table S34). The PR score of carbon storage was calculated by a weighted average of the PR scores of the benthic organism carbon fixation amount and the sediment carbon fixation amount. The ratio of the amounts of benthic organism carbon fixation to sediment carbon fixation was approximately 1:9 in all of the tidal flats, and the weighted average was also set at a 1:9 ratio.

**Table S33. Environmental factors for annual minimum benthic organism carbon fixation amount**

**Table S34. Environmental factors for annual minimum sediment carbon fixation amount**

**10.3. Data collection**

The data for benthos and sediment from 2009 to 2013 necessary for calculating the amount of carbon in organisms and sediments were collected from public survey data (MLIT, Kanto Regional Development Bureau, Tokyo International Airport Construction Development Office, 2008, 2009a, 2009b, 2010a, 2010b, 2011, 2012, 2013a, 2013b; MLIT, Kanto Regional Development Bureau, Yokohama Port and Airport Technology Investigation Office, 2009, 2011a, 2012a, 2013a, 2014a; Biodiversity Center, Nature Conservation Bureau, Ministry of the Environment, 2010, 2011, 2012, 2013; Yokohama Environmental Science Research Institute, 2010, 2014; Port and Airport Research Institute, unpublished data).The data used included benthic organism survey data for three locations in SN, two locations in UK, four locations in TR, and four locations in OR. They also included sediment survey data for three locations in SN, one location in UK, four locations in TR, and two locations in OR (Fig. S27).

**Figure S27. Location of survey stations for benthic organisms and sediment in four tidal flats: (a) SN, (b) UK, (c) TR, and (d) OR**

We used quantitative data of anoxic waters and primary productivity, for which we collected DO concentration data and Chl-*a* concentration data, respectively, from surrounding waters for 2009 to 2013 (see Section 1.3). With respect to the other environmental factors, we conducted interviews with administrators, caretaker organizations, and fishermen.

**10.4. Calculation results**

The minimum carbon fixation (*X*_10_) was calculated for each area (Table S35). More than 90% of the carbon fixation was in sediment. The value of TR in 2009, which had the highest value among all the tidal flats over the past 5 years, was defined as the reference point *X*_10,R_, and the values for the present status index (*x*_10_) were obtained (Table S35). The PR scores of benthic organism carbon fixation were the same as the PR scores for suspended material removal　(Fig. S28). The PR scores for each environmental factor of sediment carbon fixation were calculated (Fig. S29), and *T*_10_, *PR*_10_, *x*_10,F_, *I*_10_, and *S*_10_ were obtained by using Equations (1)–(4) (Table S36).

**Figure S28. Radar chart of PR_10_ scores for each environmental factor in carbon storage with benthic organisms: (a) SN, (b) UK, (c) TR, and (d) OR**

**Figure S29. Radar chart of PR_10_ scores for each environmental factor in carbon storage with sediments: (a) SN, (b) UK, (c) TR, and (d) OR**

**Table S35. Annual minimum carbon fixation in benthic organisms and sediments (*X*_10_; g-C/m^2^) and present status (*x*_10_) values for carbon storage**

**Table S36. Present status (*x*_10_), trend score (*T*_10_), PR score (*PR*_10_), likely near-term future status (*x*_10,F_), service score (*I*_10_), and sustainability score (*S*_10_) for each tidal flat for carbon storage**

**11. Degree of diversity: biodiversity (*i* = 11)**

**11.1. Setting the indicators**

Biodiversity is the foundation of ecosystem services and also has value in itself (Millennium Ecosystem Assessment, 2005; Chan et al., 2016). There are many indices showing whether the composition of the species is diverse. In this study, the Shannon-Wiener diversity index (*H'*), which can be used to evaluate diversity while accounting for population bias, was used (Shannon and Weaver, 1949). In addition, there are several definitions of diversity that vary depending the spatial range to be evaluated. The diversity in one environment is known as α diversity, the differences in diversity between different environments represent β diversity, and the species diversity of all environments in evaluating a region is called γ diversity (Crist et al., 2003). In this study, because there are tidal flats with different environments within the evaluation area, γ diversity was applied. In addition, species richness varies according to the sampling effort (Gotelli and Colwell, 2001). Therefore, the survey area and number of surveys are expected to influence *H'*, but there were insufficient data to develop an appropriate model to correct for these factors, so we did not correct for scale of the evaluation area. Instead, to reduce the influence of the sampling effort on the index (*X*_11_), we unified the sampling method of the data to be used. The following equations were used:

$X_{11}= \sum_{l=1}^{M} {H'}_{l}/M$ (S32)

${H'}_{l}= -\sum_{s=1}^{S} p_{s}lnp_{s}$ (S33)

where${H'}_{l}$ is the diversity index of the entire evaluation area in survey *l*, $M$is total number of surveys in a year, $p_{s}$ is the ratio of the number of individuals in species *s* to the total number of individuals, and *S* is the total number of species.

**11.2. Conceptual model**

We created a conceptual model of the environmental factors affecting the diversity index (Fig. S30). A healthy natural system and diverse habitats are needed to maintain or improve the diversity index. For this reason, it is important that there is little damage to ecosystems from anoxic waters and blue tides (Kodama et al., 2012) and that there is no floating sand, erosion, and subsidence. The surrounding area also needs to have healthy habitats and supply various species’ larvae. On the other hand, alien species often destroy traditional ecosystems and reduce biodiversity. In the social system, therefore, species protection activities, such as removal of alien species and maintenance and creation of diversified habitats, are important countermeasures.

**Figure S30. Conceptual model of environmental factors for degree of diversity**

Based on these results, six environmental factors were considered (Table S37). Healthy habitat, ground stability, source of juveniles, and alien species were defined as environmental factors of the natural system, and diversity of environment, and protection of species were defined in the social system.

**Table S37. Environmental factors for degree of diversity**

**11.3. Data collection**

Public survey data for benthos from 2009 to 2013 were used to calculate the diversity index (see Section 1.3). For the other environmental factors, we conducted interviews with administrators, caretaker organizations, and fishermen.

**11.4. Calculation results**

The present status (*x*_11_) was calculated from the annual average value of the diversity index (*H**'*) in each tidal flat (Table S38), with the maximum value of the past 5 years, 3.46 in OR in 2012, set as the reference point *X*_11,R_. In addition, PR scores for each environmental factor at each tidal flat were evaluated (Fig. S31) and *T*_9_, *PR*_9_, *x*_9,F_, *I*_9_, and *S*_9_ were obtained by using Equations (1)–(4) (Table S39).

**Figure S31. Radar chart of PR_11_ scores for each environmental factor in degree of diversity: (a) SN, (b) UK, (c) TR, and (d) OR**

**Table S38. Diversity index (*X*_11_, also *H****'***) and present status (*x*_11_) values for degree of diversity**

**Table S39. Present status (*x*_11_), trend score (*T*_11_), PR score (*PR*_11_), likely near-term future status (*x*_11,F_), service score (*I*_11_), and sustainability score (*S*_11_) for each tidal flat for degree of diversity**

**12. Rare species: biodiversity (*i* = 12)**

**12.1. Setting the indicators**

The extinction of species is a serious global problem, and the conservation of threatened species is an important goal to stop the loss of biodiversity (Butchart et al., 2010). Tidal flats are valuable habitats for many species and are necessary habitats for reproduction (Barbier et al., 2011). For rare species, we set the number of threatened species confirmed in the area in a year as the index $(X_{12})$. In addition, because it is important to consider the different types of threatened categories, we weighted the number of threatened species by category. The number of threatened species is expected to have bias associated with the sampling effort for both survey area and times, but there was no suitable model for sampling effort correction and the index was therefore not adjusted. The following equation was used:

$X_{12}= \sum_{i=1}^{N} (\alpha_{i}\times S_{i})$ (S34)

where $\alpha_{i}$ is the weighting coefficient for threatened category *i* (Table S40), *N* is the number of threatened categories and $S_{i}$ is the number of species in threatened category *i* for a given year. Threatened categories conformed to the definitions of the Japan Association of Benthology (2012).

**Table S40. Weighting factors for endangered species categories**

**12.2. Conceptual model**

We created a conceptual model of the environmental factors affecting the number of threatened species (Fig. S32). To maintain or increase the number of threatened species, the natural system must provide appropriate habitat. Therefore, similar to the case of degree of diversity, it is important that there is little damage to ecosystems caused by anoxic waters and blue tides; that there is no floating sand, erosion, and subsidence; and that the surrounding area has healthy habitats and that various species’ larvae are supplied. In addition, alien species and predators or competing species for threatened species will inhibit the survival of threatened species. In the social system, species protection activities, such as the removal of alien species and predatory or competitive species and the establishment of protected areas, are considered to be important.

**Figure S32. Conceptual model of environmental factors for rare species**

Based on the conceptual model, six environmental factors were considered (Table S41). Healthy habitat, ground stability, source of juveniles, alien species, and predator or competing species were defined as environmental factors of the natural system, and protection of species was defined in the social system.

**Table S41. Environmental factors for rare species**

**12.3. Data collection**

Public survey data for benthos from 2009 to 2013 were used to obtain the annual confirmed species list necessary for calculating rare species (see Section 1.3). Necessary information for the other environmental factors was collected through interviews with administrators, caretaker organizations, and fishermen.

**12.4. Calculation results**

In each tidal flat, the number of threatened species weighted by category was calculated. The reference point (*X*_12,R_) was set at 1.20, the maximum point at OR in 2009, 2012, and 2013, and the present status (*x*_12_) values were obtained (Table S42). In addition, the PR scores for each environmental factor at each tidal flat were evaluated (Fig. S33), and *T*_12_, *PR*_12_, *x*_12,F_, *I*_12_ , and *S*_12_ were obtained by using Equations (1)–(4) (Table S43).

**Figure S33. Radar chart of PR_12_ score for each environmental factor in rare species: (a) SN, (b) UK, (c) TR, and (d) OR**

**Table S42. Annual number of threatened species weighted by threatened category (*X*_12_) and present status (*x*_12_) values for rare species**

**Table S43. Present status (*x*_12_), trend score (*T*_12_), PR score (*PR*_12_), likely near-term future status (*x*_12,F_), service score (*I*_12_), and sustainability score (*S*_12_) for each tidal flat for rare species**

**References**

Akiyama, Y., Kuroiwa, H., Yamauchi, I. and Okada, T. 2017. A Quantitative Assessment of Marine Spiritual Benefits Related to a Shinto Shrine. Journal of Coastal Zone Studies 30: 91-102.

Aller R.C. 1982. The Effects of Macrobenthos on Chemical Properties of Marine Sediment and Overlying Water. In: McCall P.L., Tevesz M.J.S. (eds) Animal-Sediment Relations. Topics in Geobiology, vol 100. Springer, Boston, MA. DOI: <https://doi.org/10.1007/978-1-4757-1317-6_2>

Barbier, Edward B.; Hacker, Sally D.; Kennedy, Chris; Kock, Evamaria W.; Stier AC and BRS. 2011. The value of estuarine and coastal ecosystem services. Ecological Monographs 81:169–193. DOI: 10.1890/10-1510.1.

Biodiversity Center, Nature Conservation Bureau, Ministry of the Environment 2010. Annual report: The tidal flats and algal beds, the Monitoring Sites 1000 in FY2009, 409p, <http://www.biodic.go.jp/moni1000/findings/reports/index.html> (January, 2018).

Biodiversity Center, Nature Conservation Bureau, Ministry of the Environment. 2011. Annual report: The tidal flats and algal beds, the Monitoring Sites 1000 in FY2010, 271p, <http://www.biodic.go.jp/moni1000/findings/reports/index.html> (January, 2018).

Biodiversity Center, Nature Conservation Bureau, Ministry of the Environment. 2012. Annual report: The tidal flats and algal beds, the Monitoring Sites 1000 in FY2011, 416p, <http://www.biodic.go.jp/moni1000/findings/reports/index.html> (January, 2018).

Biodiversity Center, Nature Conservation Bureau, Ministry of the Environment. 2013. Annual report: The tidal flats and algal beds, the Monitoring Sites 1000 in FY2002, 384p, <http://www.biodic.go.jp/moni1000/findings/reports/index.html> (January, 2018).

Bretschneider CL. 1957. Chapter 3 Revisions in Wave Forecasting: Revisions in Wave Forecasting. Coastal Engineering Proceedings 6: 30–67. DOI: https://doi.org/10.9753/icce.v6.3.

Butchart, S. H. M., Walpole, M., Collen, B., Van Strien, A., Scharlemann, J. P. W., Almond, R. E. A., … Watson, R. 2010. Global biodiversity: Indicators of recent declines. Science, 328(5982), 1164–1168. DOI: <https://doi.org/10.1126/science.1187512>

Chan KMA., Balvanera P., Benessaiah K., Chapman M., Díaz S., Gómez-Baggethun E., Gould R., Hannahs N., Jax K., Klain S., Luck GW., Martín-López B., Muraca B., Norton B., Ott K., Pascual U., Satterfield T., Tadaki M., Taggart J., Turner N. 2016. Opinion: Why protect nature? Rethinking values and the environment. Proceedings of the National Academy of Sciences 113:1462–1465. DOI: 10.1073/pnas.1525002113.

Central Block Council for Fisheries Industry R&D. 2013. Edo-style Revival! Towards the Restoration of the Tokyo Bay, 35p, [http://nria.fra.affrc.go.jp/ hakko/Teigen.pdf](http://nria.fra.affrc.go.jp/%20hakko/Teigen.pdf) (January, 2018).

Crist, T. O., Veech, J. A., Gering, J. C., & Summerville, K. S. 2003. Partitioning Species Diversity across Landscapes and Regions: A Hierarchical Analysis of a, b, and g Diversity. The American Naturalist, 162(6), 734–743.

Chiba Prefecture Commerce and Industry Labor Tourism Planning Division. 2010. Reports of tourism entry survey in FY 2009, 32p. <https://www.pref.chiba.lg.jp/kankou/toukeidata/kankoukyaku/documents/h21gaiyou.pdf> (January, 2018).

Chiba Prefecture Commerce and Industry Labor Tourism Planning Division. 2011. Reports of tourism entry survey in FY 2010, 32p. https://www.pref.chiba.lg.jp/kankou/toukeidata/kankoukyaku/documents/h22irikomi.pdf (January, 2018).

Chiba Prefecture Commerce and Industry Labor Tourism Planning Division. 2012. Reports of tourism entry survey in FY 2011, 36p. https://www.pref.chiba.lg.jp/kankou/toukeidata/kankoukyaku/documents/h23irikomi.pdf (January, 2018).

Chiba Prefecture Commerce and Industry Labor Tourism Planning Division. 2013. Reports of tourism entry survey in FY 2012, 36p. https://www.pref.chiba.lg.jp/kankou/toukeidata/kankoukyaku/documents/h24irikomi.pdf (January, 2018).

Chiba Prefecture Commerce and Industry Labor Tourism Planning Division. 2014. Reports of tourism entry survey in FY 2013, 34p. https://www.pref.chiba.lg.jp/kankou/toukeidata/kankoukyaku/documents/h25houkokusyo.pdf (January, 2018).

Epstein, S. 1997. Microbial Food Webs in Marine Sediments. I. Trophic Interactions and Grazing Rates in Two Tidal Flat Communities. Microb Ecol 34: 188-198.DOI: https://doi.org/10.1007/s002489900048

Fourqurean JW., Duarte CM., Kennedy H., Marbà N., Holmer M., Mateo MA., Apostolaki ET., Kendrick GA., Krause-Jensen D., McGlathery KJ., Serrano O. 2012. Seagrass ecosystems as a globally significant carbon stock. Nature Geoscience 5:505–509. DOI: 10.1038/ngeo1477.

Furukawa, K. and Okada, T. 2006. Tokyo Bay: its environmental status - past, present and future -, in: E. Wolanski (Ed.), The Environment in Asia Pacific Harbors, Springer, pp. 15-34.

Garcia Rodrigues J., Conides A., Rivero Rodriguez S., Raicevich S., Pita P., Kleisner K., Pita C., Lopes P., Alonso Roldán V., Ramos S., Klaoudatos D., Outeiro L., Armstrong C., Teneva L., Stefanski S., Böhnke-Henrichs A., Kruse M., Lillebø A., Bennett E., Belgrano A., Murillas A., Sousa Pinto I., Burkhard B., Villasante S. 2017. Marine and Coastal Cultural Ecosystem Services: knowledge gaps and research priorities. One Ecosystem 2:e12290. DOI: 10.3897/oneeco.2.e12290.

Goda, Y. 1970. A synthesis of breaker indices. Journal of Japan Society of Civil Engineers 180: 39-49. DOI: https://doi.org/10.2208/jscej1969.1970.180_39

Gotelli, N. J., & Colwell, R. K. 2001. Quantifying biodiversity: procedures and pitfalls in the measurment and comparison of species richness. Ecology Letters, 4(May 1988), 379–391. <https://doi.org/10.1046/j.1461-0248.2001.00230.x>

Goulletquer, P., Bacher, C. 1988. Empirical modehng of the growth of *Ruditapes phiLippinarum* by means of non linear regression on factorial coordmates. Aquat Liv Res 1:141-154. DOI: https://doi.org/10.1051/alr:1988016

Halpern BS., Longo C., Stewart Lowndes JS., Best BD., Frazier M., Katona SK., Kleisner KM., Rosenberg AA., Scarborough C., Selig ER. 2015. Patterns and emerging trends in global ocean health. PLoS ONE 10. DOI: 10.1371/journal.pone.0117863.

Hosokawa, Y., Kibe, E., Miyoshi, E., Kuwae, T. and Furukawa T. 1996. Distribution of areal filtration rate of short-necked clam in coastal tidal flat. Technical Note of the Port and Harbour Research Institute Ministry of Land, Infrastructure and Transport, Japan, (844), 3–21. http://ci.nii.ac.jp/naid/40001275569/en/ (January, 2018)

Japan Oceanographic Data Center: JODC Data On-line Service System.

http://www.jodc.go.jp/jodcweb/JDOSS/index.html (January, 2018)

Japanese Fisheries Agency Marino Forum 21. 2007. The Manual for Evaluating the Health of Sandy Tidal Flat, 33p.

Japan Association of Benthology ed. 2012. Threatened animals of Japanese tidal flats: Red data book of seashore benthos. Kanagawa: Tokai University Press.

Kakino, J. 1992. Recent situation on the Japanese littleneck fisheries. Fisheries Engineering, 29, 31–39. DOI: <https://doi.org/10.18903/fisheng.29.1_31>

Kakino, J. 2000. Dispersal of Japanese littleneck clam Ruditapes philippinarum (ADAMS and REEVE) in relation to change of bottom level dur to wave action on Banzu tidal flat, Tokyo Bay. Fisheries Engineering 37: 115-128.

Kanagawa Prefecture Tourism Promotion Council. 2010. Kanagawa prefecture inclusion tourists survey report, 29p. <http://www.pref.kanagawa.jp/docs/ya3/cnt/f80022/documents/49503.pdf> (January, 2018)

Kanagawa Prefecture Tourism Promotion Council. 2011. Kanagawa prefecture inclusion tourists survey report in FY 2010, 29p. http://www.pref.kanagawa.jp/docs/ya3/cnt/f80022/documents/369064.pdf (January, 2018)

Kanagawa Prefecture Tourism Promotion Council. 2012. Kanagawa prefecture inclusion tourists survey report in FY 2011, 29p. http://www.pref.kanagawa.jp/docs/ya3/cnt/f80022/documents/459335.pdf (January, 2018)

Kanagawa Prefecture Tourism Promotion Council. 2013. Kanagawa prefecture inclusion tourists survey report in FY 2012, 28p. http://www.pref.kanagawa.jp/docs/ya3/cnt/f80022/documents/459335.pdf (January, 2018)

Kanagawa Prefecture Tourism Promotion Council. 2014. Kanagawa prefecture inclusion tourists survey report in FY 2013, 28p. http://www.pref.kanagawa.jp/docs/ya3/cnt/f80022/documents/623910.pdf (January, 2018)

Kaspar, H.F. 1983. Denitrification, nitrate reduction to ammonium, and inoganic nitrogen pools in intertidal sediments. Marine Biolgy 74: 133-139. DOI:https://doi.org/10.1007/BF00413916

Kisarazu City. 2012. History of Kisarazu. Chiba: Adomakes Press.

Klaassen W., Spilmont N. 2012. Inter-annual variability of CO_2_ exchanges between an emersed tidal flat and the atmosphere. Estuarine, Coastal and Shelf Science 100:18–25. DOI: 10.1016/j.ecss.2011.06.002.

Kodama. K., Lee, J. H., Oyama, M., Shiraishi, H. and Horiguchi, T. 2012. Disturbance of benthic macrofauna in relation to hypoxia and organic enrichment in a eutrophic coastal bay. Marine Environmental Research 76:80-89. DOI: https://doi.org/10.1016/j.marenvres.2011.08.007.

Kohata, K., Hiwatari, T. and Hagiwara, T. 2003. Natural water-purification system observed in a shallow coastal lagoon: Matsukawa-ura, Japan. Marine Pollution Bulletin 47: 148-154. DOI: https://doi.org/10.1016/S0025-326X(03)00055-9

Magni P., Montani S., Takada C., Tsutsumi H. 2000. Temporal scaling and relevance of bivalve nutrient excretion on a tidal flat of the Seto Inland Sea, Japan. Marine Ecology Progress Series 198:139–155. DOI: 10.3354/meps198139.

Mase, H., Matsumoto, A. and Iwagaki Y. 1986. Calculation model of random wave transformation in shallow water. Journal of Japan Society of Civil Engineers 375: 221-230. DOI: https://doi.org/10.2208/jscej.1986.375_221

Migné, A., Davoult, D., Spilmont, N., Menu, D., Boucher, G., Gattuso, J.-P., Rybarczyk, H. 2002. A closed chamber CO_2_-flux method for estimating inter- tidal primary production and respiration under emersed conditions. Marine Biology 140: 865-869. DOI: 10.1007/s00227-001-0741-1

Migné, A., Spilmont, N., Davoult, D. 2004. In situ measurements of benthic primary production during emersion: seasonal variations and annual production in the Bay ofSomme (eastern English Channel, France). Continental Shelf Research 24, 1437-1449. DOI: <https://doi.org/10.1016/j.csr.2004.06.002>

Millennium Ecosystem Assessment (MEA). 2005. Ecosystem and Human Well Being: Synthesis. Island Press.

Ministry of Land, Infrastructure, Transport and Tourism (MLIT), Kanto Regional Development Bureau, Tokyo International Airport Construction Office. 2008. Annual report: The sea area, Haneda airport environmental survey in FY2008, 444p

Ministry of Land, Infrastructure, Transport and Tourism, Kanto Regional Development Bureau, Tokyo International Airport Construction Office. 2009a. Annual report II: The sea area, Haneda airport environmental survey in FY2008, 305p

Ministry of Land, Infrastructure, Transport and Tourism, Kanto Regional Development Bureau, Tokyo International Airport Construction Office. 2009b. Annual report: The sea area, Haneda airport environmental survey in FY2009, 282p

Ministry of Land, Infrastructure, Transport and Tourism, Kanto Regional Development Bureau, Tokyo International Airport Construction Office. 2010a. Annual report II: The sea area, Haneda airport environmental survey in FY2009, 279p

Ministry of Land, Infrastructure, Transport and Tourism, Kanto Regional Development Bureau, Tokyo International Airport Construction Office. 2010b. Annual report: The sea area, Haneda airport environmental survey in FY2010, 382p

Ministry of Land, Infrastructure, Transport and Tourism, Kanto Regional Development Bureau, Tokyo International Airport Construction Office. 2011. Annual report II: The sea area, Haneda airport environmental survey in FY2010, 262p

Ministry of Land, Infrastructure, Transport and Tourism, Kanto Regional Development Bureau, Tokyo International Airport Construction Office. 2012. Annual report: The sea area, Haneda airport environmental survey in FY2011, 365p

Ministry of Land, Infrastructure, Transport and Tourism, Kanto Regional Development Bureau, Tokyo International Airport Construction Office. 2013a. Annual report: The sea area, Haneda airport environmental survey in FY2012, 536p

Ministry of Land, Infrastructure, Transport and Tourism, Kanto Regional Development Bureau, Tokyo International Airport Construction Office. 2013b. Annual report II: The sea area, Haneda airport environmental survey in FY2013, 618p

Ministry of Land, Infrastructure, Transport and Tourism, Kanto Regional Development Bureau, Yokohama Port and Airport Technology Investigation Office. 2009. Report of water quality and sediment condition survey on artificial beach in FY2008, 224p

Ministry of Land, Infrastructure, Transport and Tourism, Kanto Regional Development Bureau, Yokohama Port and Airport Technology Investigation Office. 2010. Report of Tokyo bay environmental monitoring in FY2009, 117p.

Ministry of Land, Infrastructure, Transport and Tourism, Kanto Regional Development Bureau, Yokohama Port and Airport Technology Investigation Office. 2011a. Report of organism and habitat survey on seawall supporting symbiotic environment in FY2010, 3-57.

Ministry of Land, Infrastructure, Transport and Tourism, Kanto Regional Development Bureau, Yokohama Port and Airport Technology Investigation Office. 2011b. Report of Tokyo bay environmental monitoring in FY2010, 138p.

Ministry of Land, Infrastructure, Transport and Tourism, Kanto Regional Development Bureau, Yokohama Port and Airport Technology Investigation Office. 2012a. Report of organism and habitat survey on seawall supporting symbiotic environment in FY2011, 5-15.

Ministry of Land, Infrastructure, Transport and Tourism, Kanto Regional Development Bureau, Yokohama Port and Airport Technology Investigation Office. 2012b. Report of Tokyo bay environmental monitoring in FY2011, 130p.

Ministry of Land, Infrastructure, Transport and Tourism, Kanto Regional Development Bureau, Yokohama Port and Airport Technology Investigation Office. 2013a. Report of organism and habitat survey on seawall supporting symbiotic environment in FY2012,71.

Ministry of Land, Infrastructure, Transport and Tourism, Kanto Regional Development Bureau, Yokohama Port and Airport Technology Investigation Office. 2013b. Report of Tokyo bay environmental monitoring in FY2012, 172p.

Ministry of Land, Infrastructure, Transport and Tourism, Kanto Regional Development Bureau, Yokohama Port and Airport Technology Investigation Office. 2014a. Report of environmental resilience in Keihin port in FY2013, 92p.

Ministry of Land, Infrastructure, Transport and Tourism, Kanto Regional Development Bureau, Yokohama Port and Airport Technology Investigation Office. 2014b. Report of Tokyo bay environmental monitoring in FY2013, 141p.

Ministry of Land, Infrastructure, River Bureau: Survey on actual situation of river space utilization in River environmental data base. <http://mizukoku.nilim.go.jp/ksnkankyo/mizukokuweb/kuukan/index.htm> (January, 2018)

Miura, H., Ito, Y., Yoshida, T. 2013. : Ecosystem Structure of a Fishing Port, and Presumption of a living Thing standing Stock, J. JSCE, ser. B2 (Coastal engineering) 69: I_1211-I_1215. DOI: https://doi.org/10.2208/kaigan.69.I_1211

Nagasoe S., Yurimoto T., Suzuki K., Maeno Y., Kimoto K. 2011. Effects of hydrogen sulfide on the feeding activity of Manila clam Ruditapes philippinarum. Aquatic Biology 13:293–302. DOI: 10.3354/ab00374.

Nellemann, C., Corcoran, E., Durate, C. M. Valdes, L., De Young, C., Fonseca, L. and Grimsditch G. 2009. Blue Carbon. A rapid response assessment. United Nation Environment Programme, GRID-Arendal. http://www.grida.no/ (January, 2018)

Ogawa, K. 2009. Some viewpoints of environmental education considered from development of nature conservation education in Japan. Japanese Journal of Environmental Education, 19-1, pp. 68-76. DOI: <https://doi.org/10.5647/jsoee.19.1_68>

Padres R., Canu DM., Rossi R. 2000. Modeling the growth of Tapes philippinarum in Northern Adriatic lagoons. Marine Ecology Progress Series 199: 137-148. DOI: 10.3354/meps199137.

Prins TC, Smaal AC. 1994. The role of the blue mussel Mytilus eduiis in the cycling of nutrients in Oosterschelde estuary (the Netherlands). Hydrobiologia 282: 413-429. DOI: <https://doi.org/10.1007/BF00024645>

Schwinghamer, P., Hargravem B., Peer, D. and Hawkins CM. 1986. Partitioning of production and respiration among size groups pf organisms in an intertidal benthic community. Marine Ecology Progress Series 31:131–142.

Shannon, C.E. & Weaver, W. 1949. The mathematical theory of communication. The University of Illinois Press,Urbana, 117pp.

Spilmont, N., Migné, A., Lefevre, A., Artigas, L.F., Rauch, M., Davoult, D. 2005. Temporal variability of intertidal benthic metabolism under emersed conditions in an exposed sandy beach (Wimereux, eastern English Channel, France). Journal of Sea Research 53, 161-167. DOI: https://doi.org/10.1016/j.seares.2004.07.004

Subcommittee on Monitoring of the Tokyo Bay Renaissance Promotion Conference. 2010. Report of Tokyo Bay General Survey for Water Environment in 2009. <http://www1.kaiho.mlit.go.jp/KANKYO/TB_Renaissance/Information/20090907publicity.pdf> (January, 2018)

Subcommittee on Monitoring of the Tokyo Bay Renaissance Promotion Conference. 2011. Tokyo Bay General Survey for Water Environment in 2010. <http://www1.kaiho.mlit.go.jp/KANKYO/TB_Renaissance/Monitoring/General_survey/GS_report2010.pdf> (January, 2018)

Subcommittee on Monitoring of the Tokyo Bay Renaissance Promotion Conference. 2012. Tokyo Bay General Survey for Water Environment in 2011. <http://www1.kaiho.mlit.go.jp/KANKYO/TB_Renaissance/Monitoring/General_survey/GS_report2011.pdf> (January, 2018)

Subcommittee on Monitoring of the Tokyo Bay Renaissance Promotion Conference. 2013. Tokyo Bay General Survey for Water Environment in 2012. <http://www1.kaiho.mlit.go.jp/KANKYO/TB_Renaissance/Monitoring/General_survey/GS_report2012.pdf> (January, 2018)

Subcommittee on Monitoring of the Tokyo Bay Renaissance Promotion Conference. 2014. Tokyo Bay General Survey for Water Environment in 2013. <http://www1.kaiho.mlit.go.jp/KANKYO/TB_Renaissance/Monitoring/General_survey/GS_report2013.pdf> (January, 2018)

Tamada, T., Mase, H.，Yasuda, T. 2009. Random Wave Runup Formulae for Seawall with Composite Cross Section. J. JSCE, ser. B2 (Coastal engineering) 65: 936-940. (in Japanese)

Toba M., Kosemura T., Yamakawa H., Sugiura Y., Kobayashi, Y. 2008. Field and laboratory observations on the hypoxic impact on survival and distribution of short-necked clam Ruditapes philippinarum larvae in Tokyo Bay , central Japan. Plankton Benthos Research 3:165–173. DOI: 10.3800/pbr.3.165.

Ursin E. 1967. A mathematical model of some aspects of fish growth, respiration and mortality. Journal of the Fisheries Research Board of Canada 24: 2355-2453. DOI: https://doi.org/10.1139/f67-190

Uzaki N., Kai M., Aoyama H., Suzuki T. 2003. Changes in mortality rate and glycogen content of the Manila clam *Ruditapes philippinarum* during the development of oxygen-deficient waters. Fisheries Science 69:936–943. DOI: 10.1046/j.1444-2906.2003.00710.x.

Water Environment Committee of the Ministry of the Environment Central Environment Council. 2007. The Eighth Meeting of the Expert Committee on the Water Quality Standards for the Conservation of Aquatic Life. <https://www.env.go.jp/council/09water/y0910-08b.html> (January, 2018).

Yokohama Environmental Planning Bureau. 2009. Results of the FY 2009 Water Quality Survey of Public Water Areas in Yokohama. <http://www.city.yokohama.lg.jp/kankyo/mamoru/kanshi/index09.html> (January, 2018)

Yokohama Environmental Planning Bureau. 2010. Results of the FY 2010 Water Quality Survey of Public Water Areas in Yokohama. <http://www.city.yokohama.lg.jp/kankyo/mamoru/kanshi/index10.html> (January, 2018)

Yokohama Environmental Planning Bureau. 2011. Results of the FY 2011 Water Quality Survey of Public Water Areas in Yokohama, <http://www.city.yokohama.lg.jp/kankyo/mamoru/kanshi/index11.html> (January, 2018)

Yokohama Environmental Planning Bureau. 2012. Results of the FY 2012 Water Quality Survey of Public Water Areas in Yokohama, <http://www.city.yokohama.lg.jp/kankyo/mamoru/kanshi/index12.html>. (January, 2018)

Yokohama Environmental Planning Bureau. 2013. Results of the FY 2013 Water Quality Survey of Public Water Areas in Yokohama, <http://www.city.yokohama.lg.jp/kankyo/mamoru/kanshi/index13.html> (January, 2018).

Yokohama Environmental Science Research Institute. 2010. the survey reports River and Marine Organisms in Yokohama, Yokohama Environmental Planning Bureau, No.12, 188p.

Yokohama Environmental Science Research Institute. 2014. the survey reports River and Marine Organisms in Yokohama, Yokohama Environmental Planning Bureau, No.13, 266p.

Yokohama Offshore Environmental Conservation Agency. 2000. The reports environmental survey of marine park waters area in FY1999, pp.15-17.
