# Supplementary figures and images for: Method for the quantitative evaluation of ecosystem services in coastal regions
Source: PeerJ. 2019 Jan 14;6:e6234. doi: 10.7717/peerj.6234 (PMC6336092; doi:10.7717/peerj.6234)

# Social System

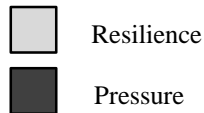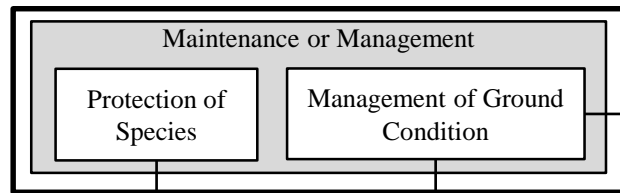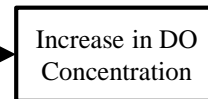

# Natural System

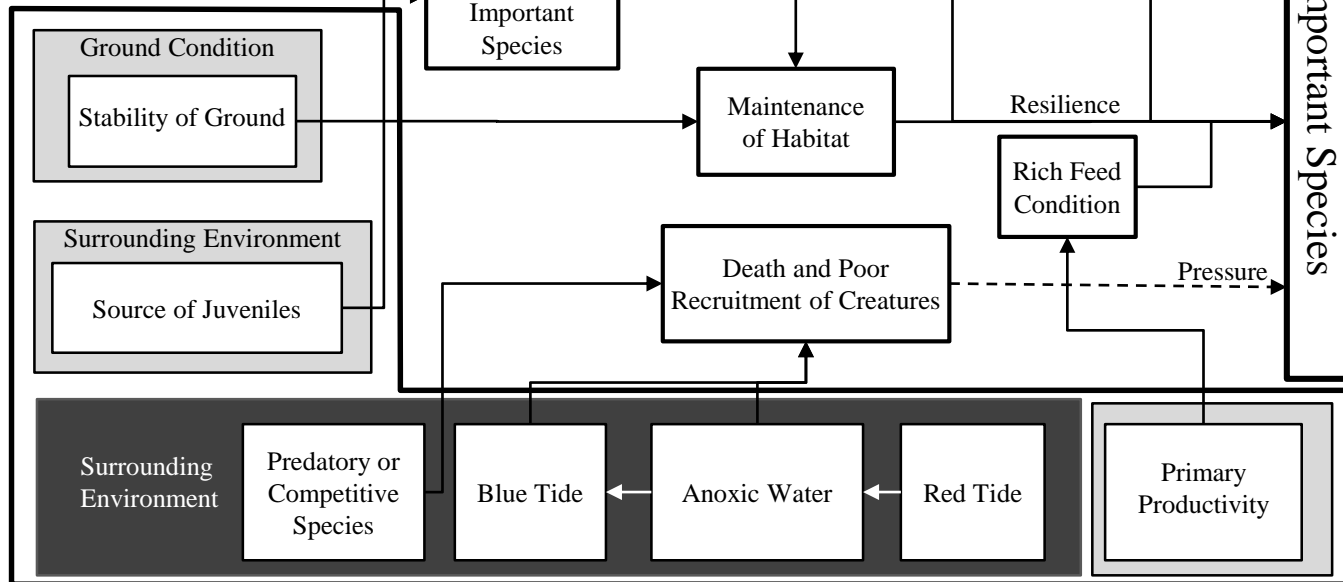

Supplement: Supplemental Information 2 [file peerj-07-6234-s002.pdf]

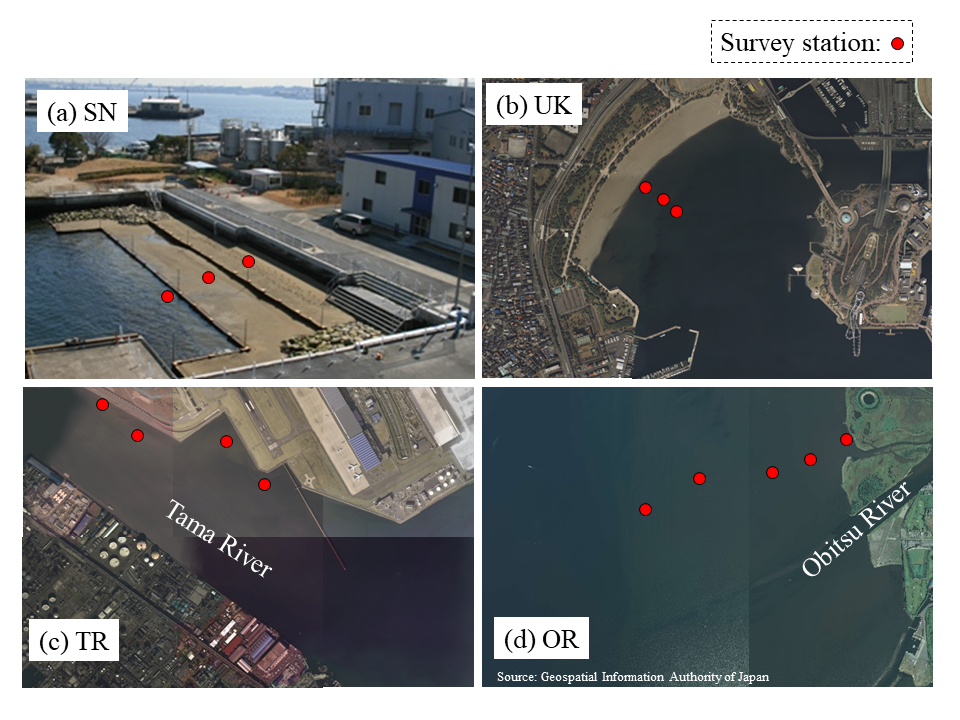

Supplement: Supplemental Information 3 — (d) source: aerial photograph of Geospatial Information Authority of Japan, https://mapps.gsi.go.jp/. [file peerj-07-6234-s003.png]

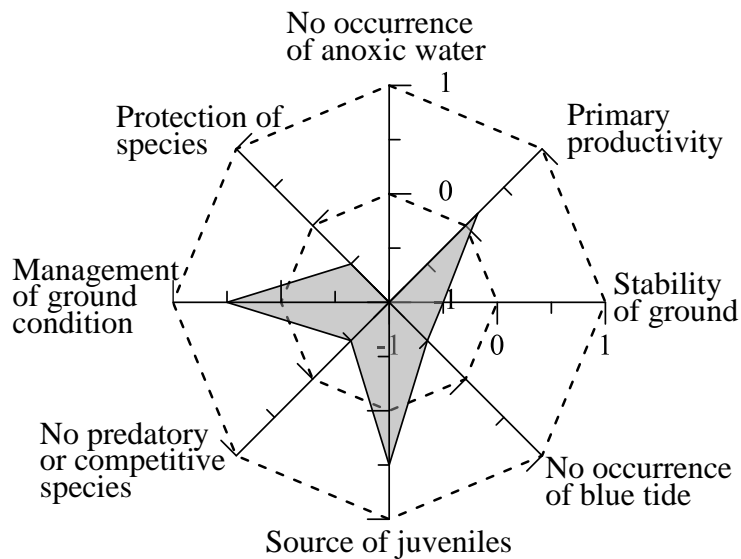

(a)

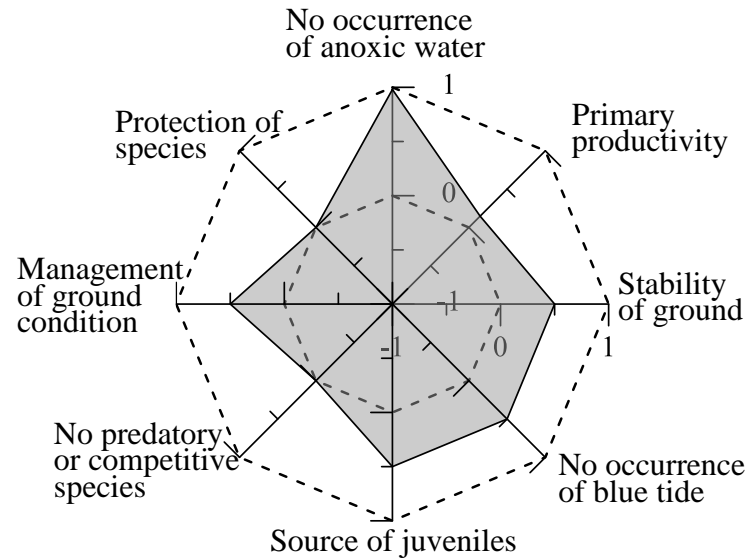

(b)

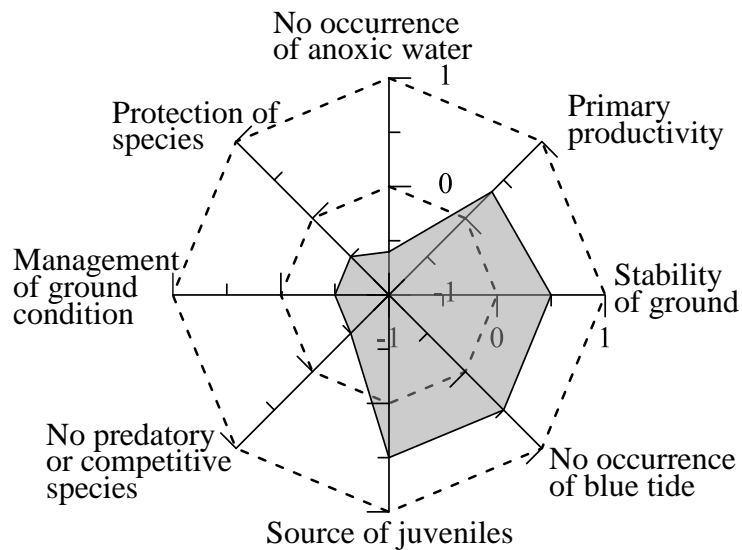

(c)

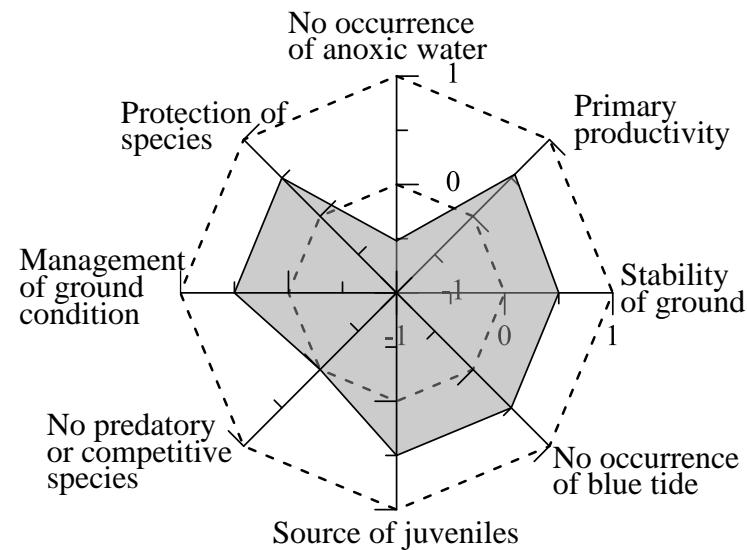

(d)

Supplement: Supplemental Information 4 [file peerj-07-6234-s004.pdf]

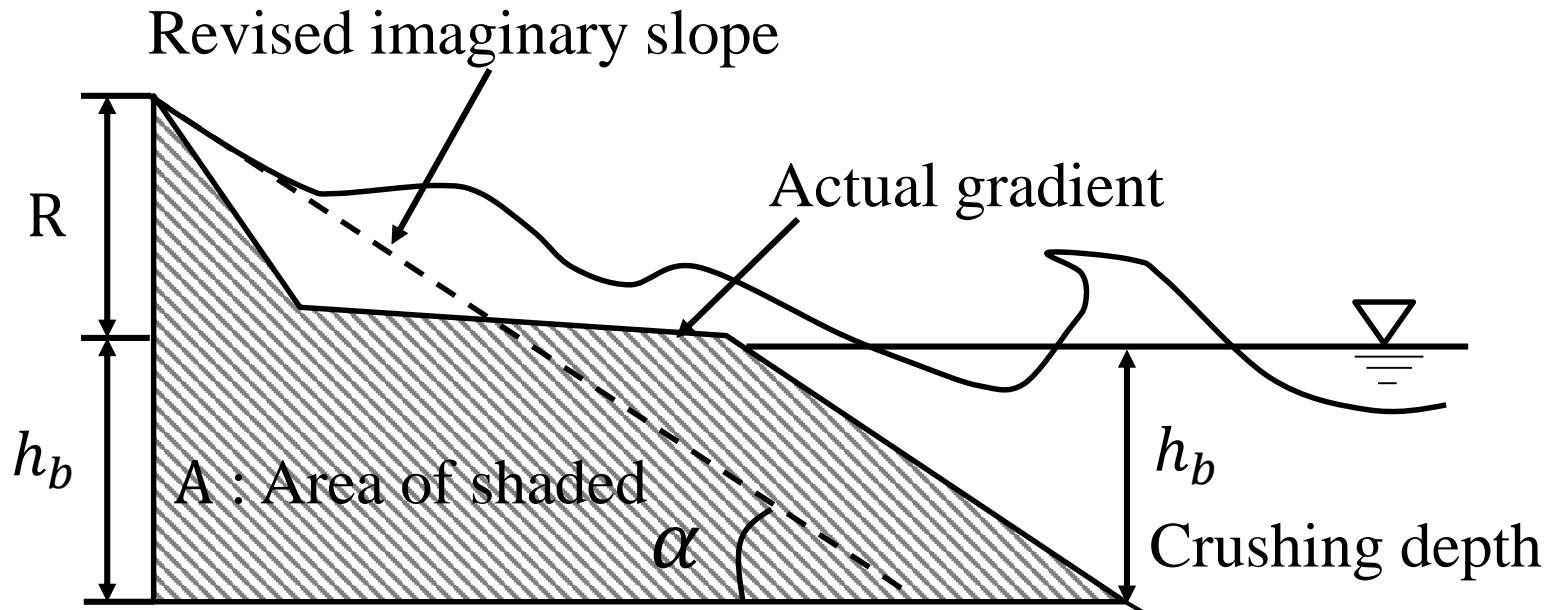

$$\cot \alpha = \frac{2A}{(h_b + R)^2}$$

Supplement: Supplemental Information 5 [file peerj-07-6234-s005.pdf]

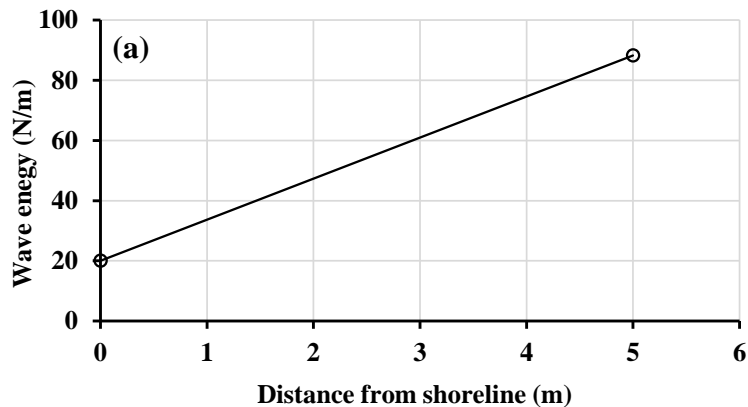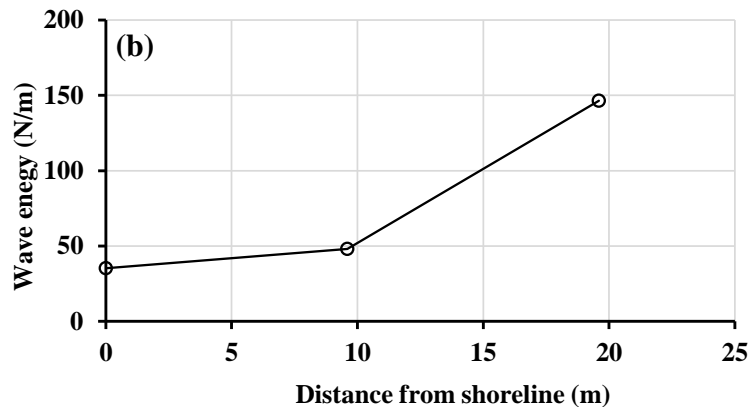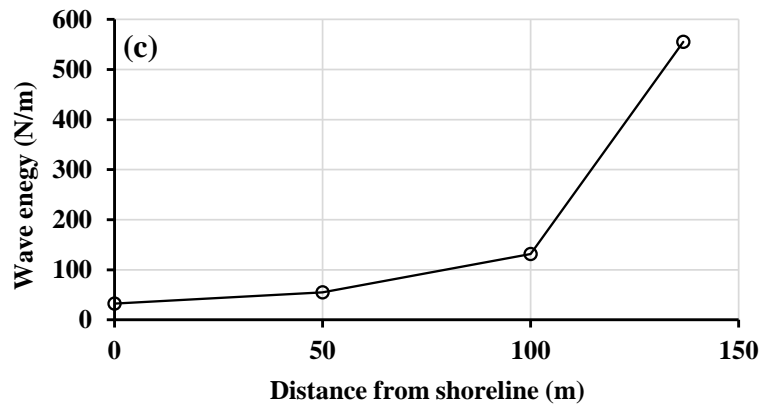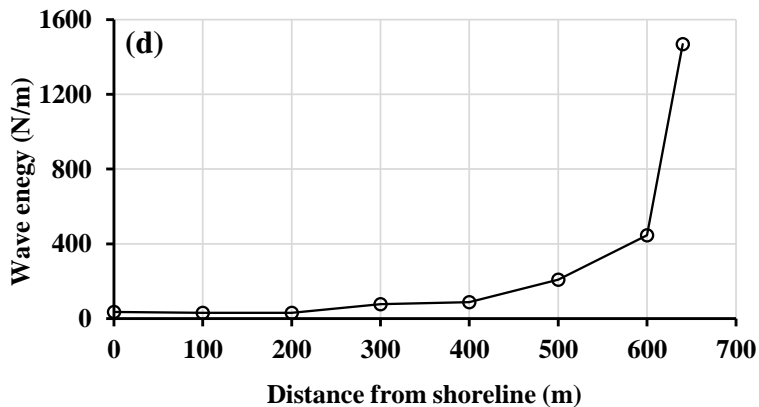

Supplement: Supplemental Information 7 [file peerj-07-6234-s007.pdf]

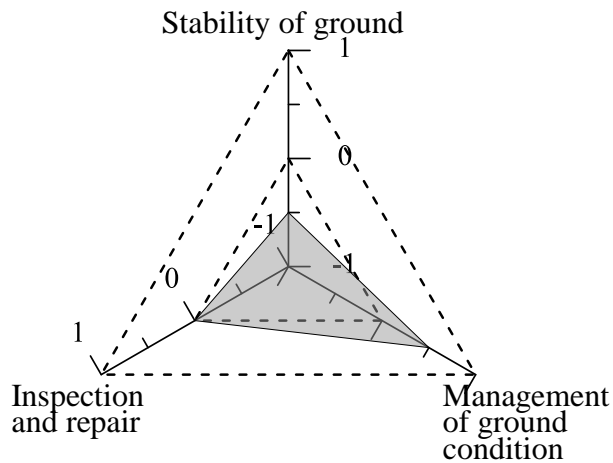

(a)

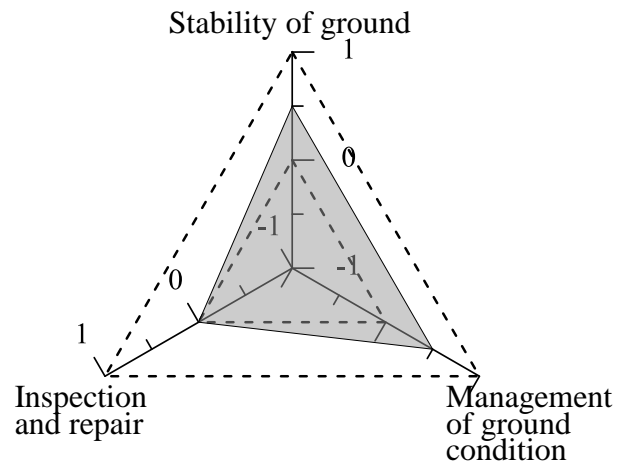

(b)

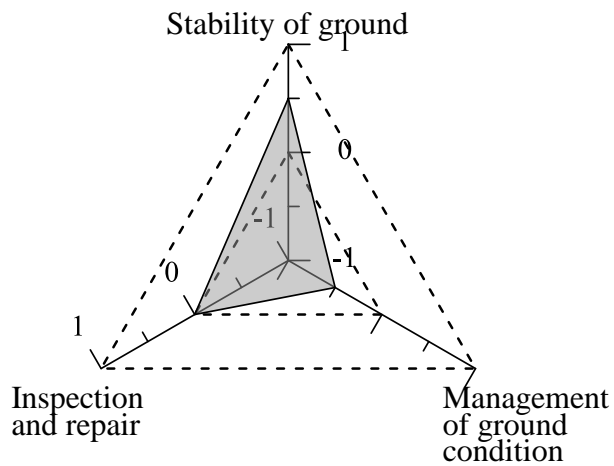

(c)

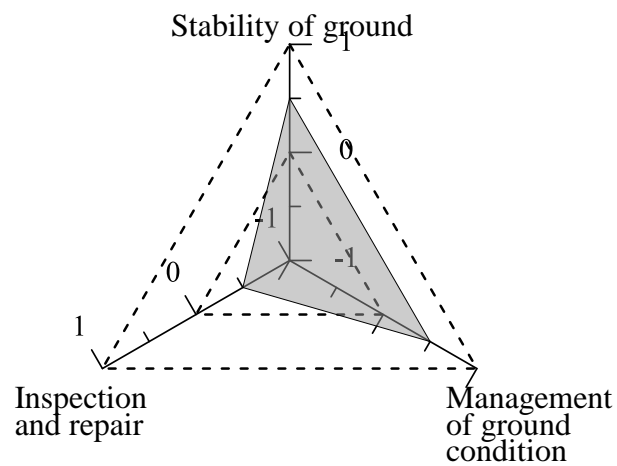

(d)

Supplement: Supplemental Information 8 [file peerj-07-6234-s008.pdf]

# Social System

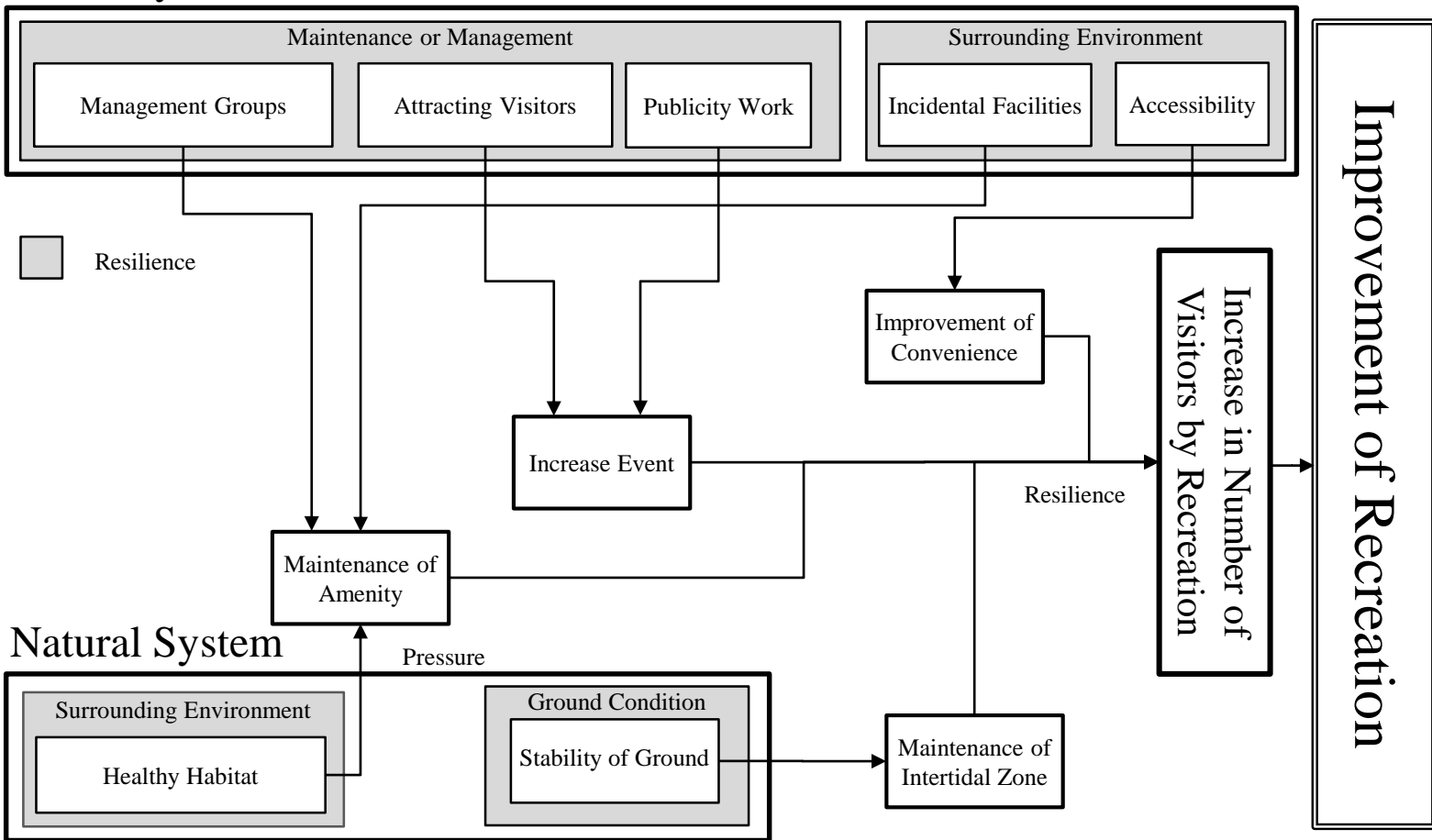

Supplement: Supplemental Information 9 [file peerj-07-6234-s009.pdf]

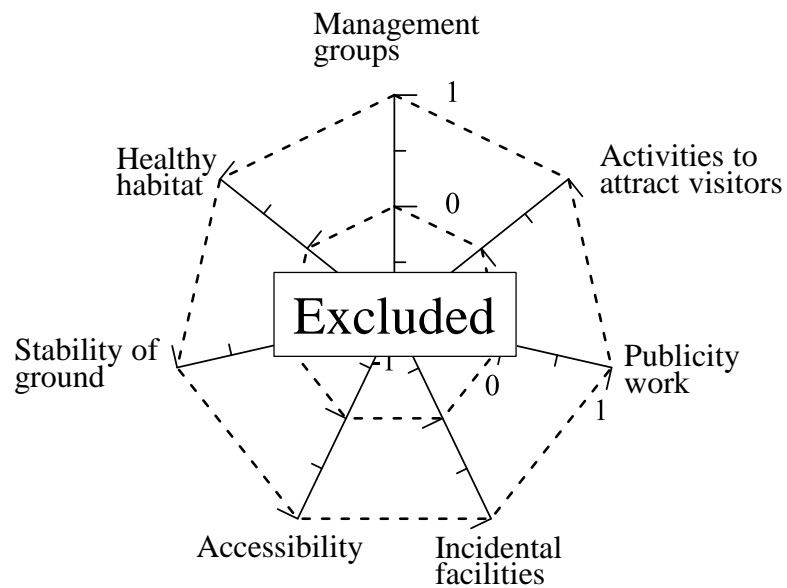

(a)

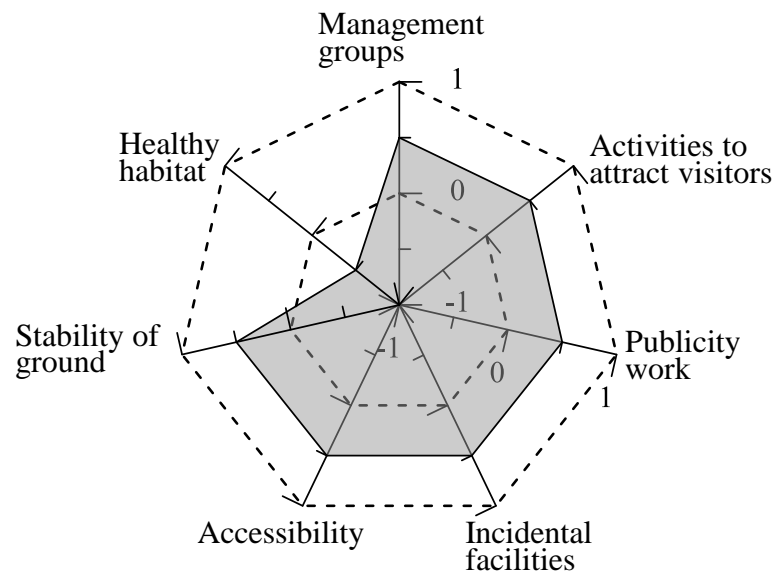

(b)

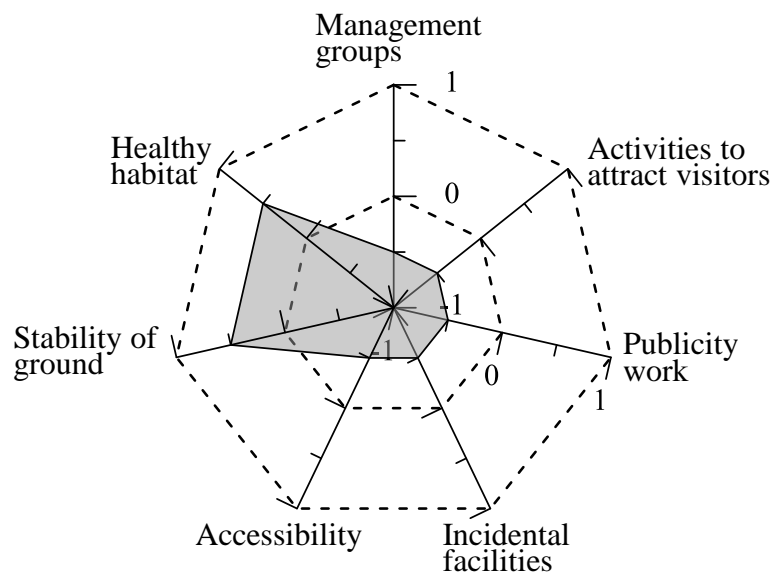

(c)

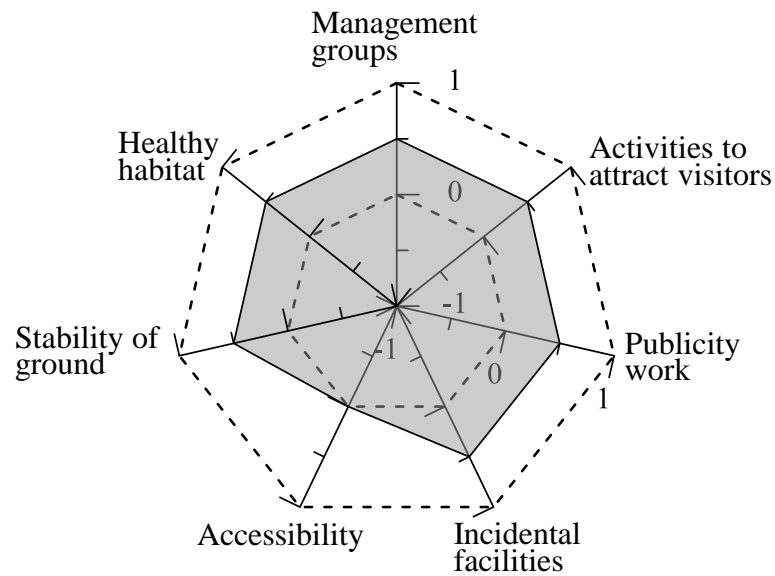

(d)

Supplement: Supplemental Information 10 — SN was excluded because recreation is not allowed in this area. [file peerj-07-6234-s010.pdf]

Social System

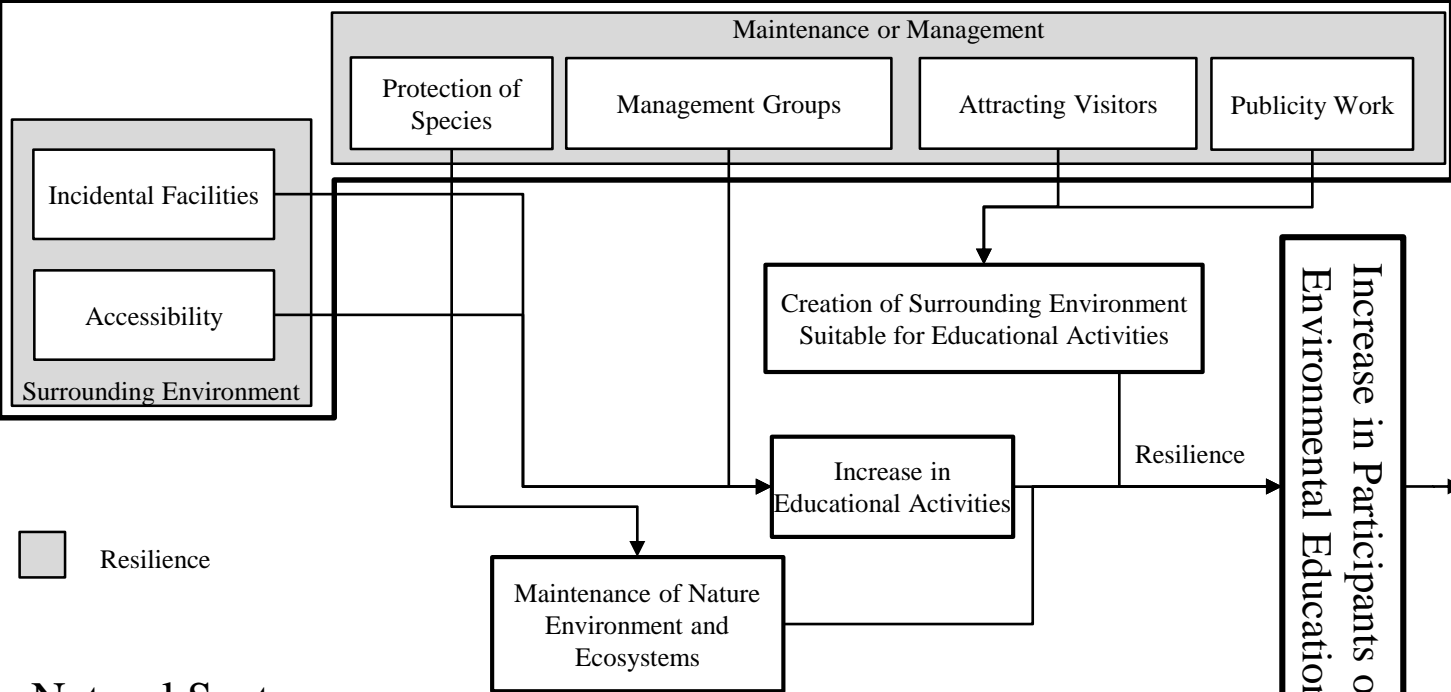

Resilience

Natural System

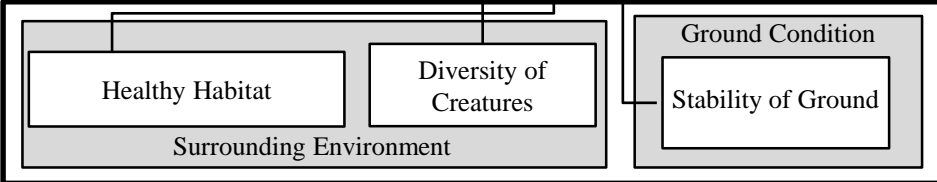

Spread and Improvement of Environmental Education

Supplement: Supplemental Information 11 [file peerj-07-6234-s011.pdf]

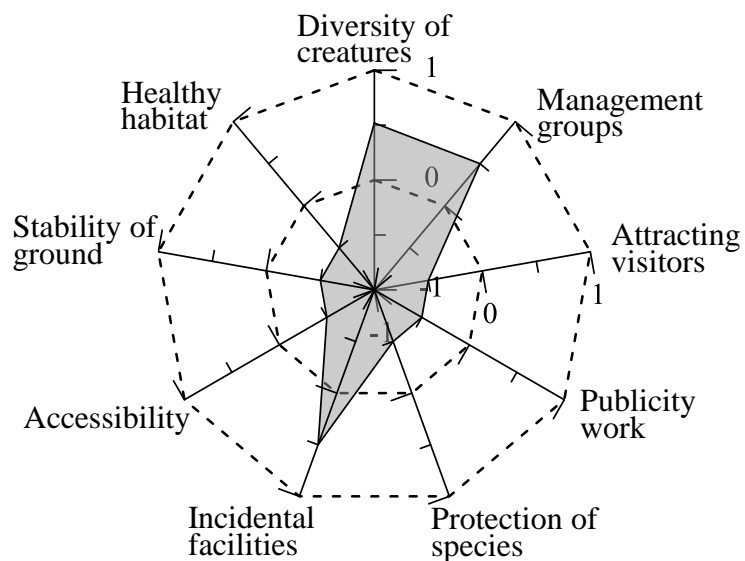

(a)

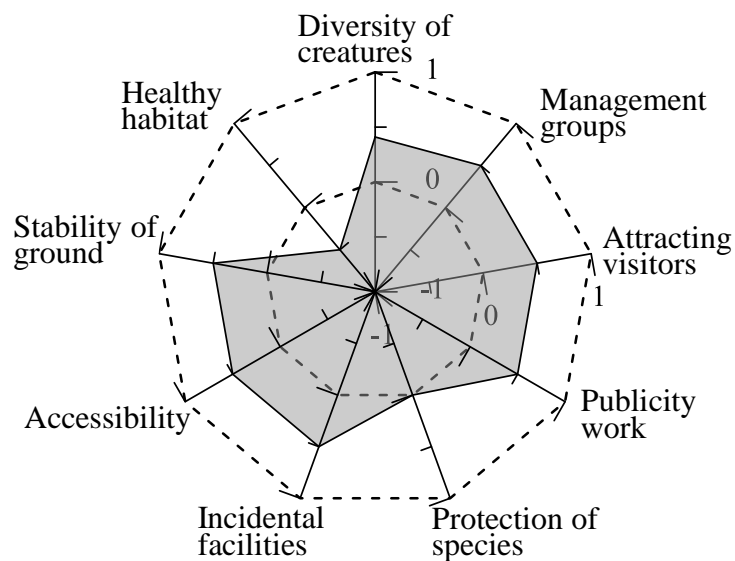

(b)

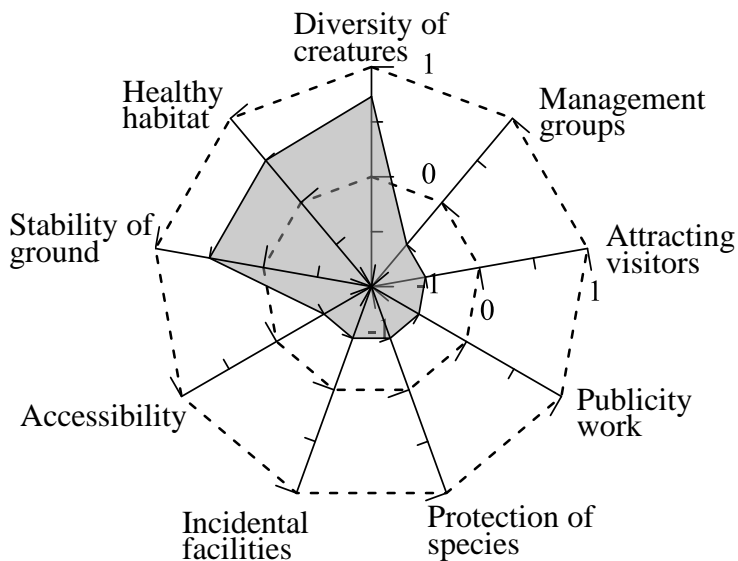

(c)

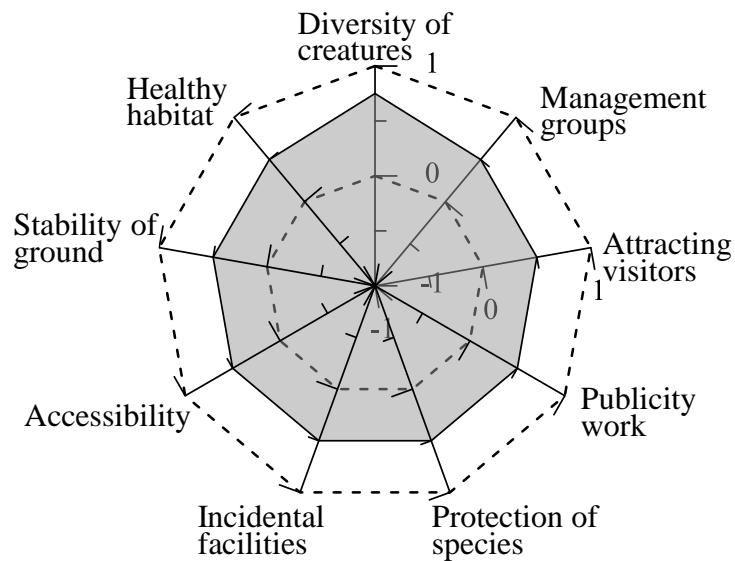

(d)

Supplement: Supplemental Information 12 [file peerj-07-6234-s012.pdf]

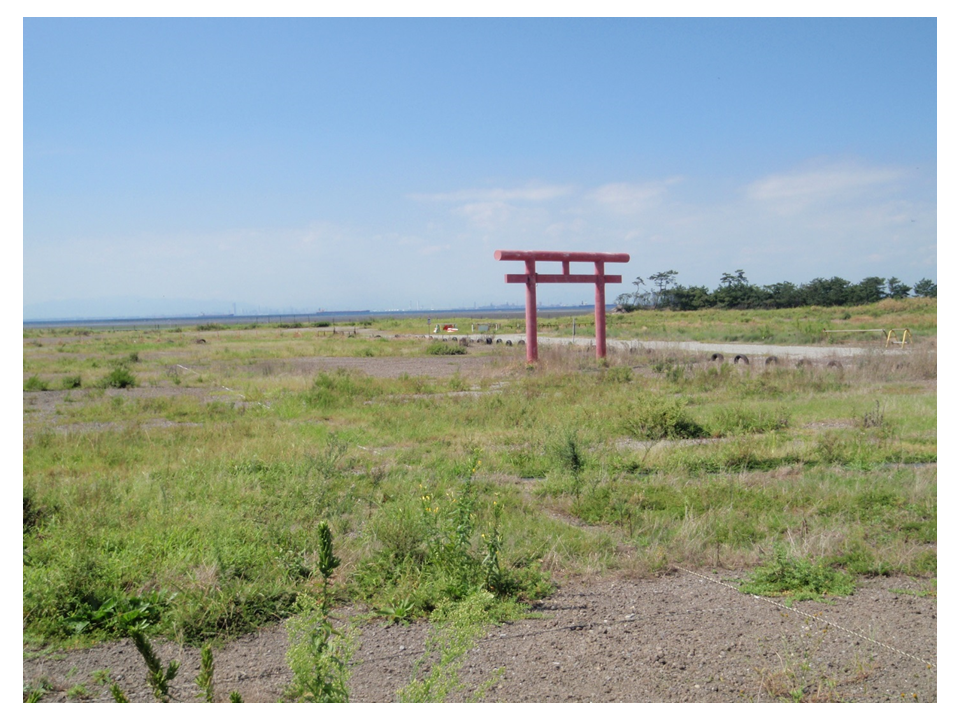

Supplement: Supplemental Information 14 [file peerj-07-6234-s014.png]

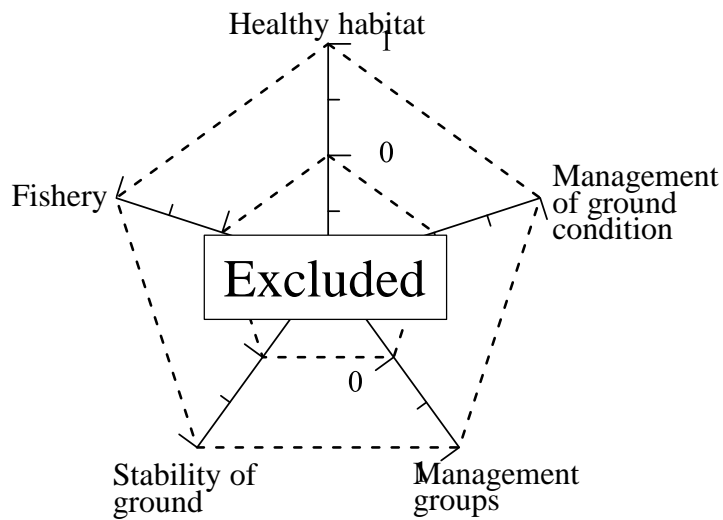

(a)

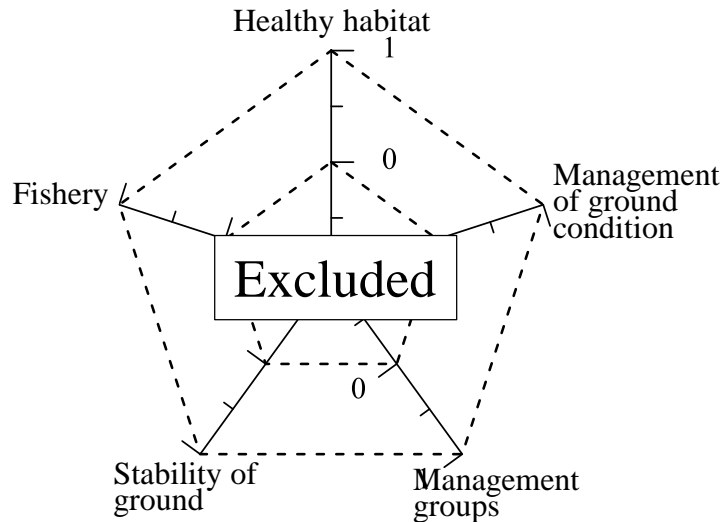

(b)

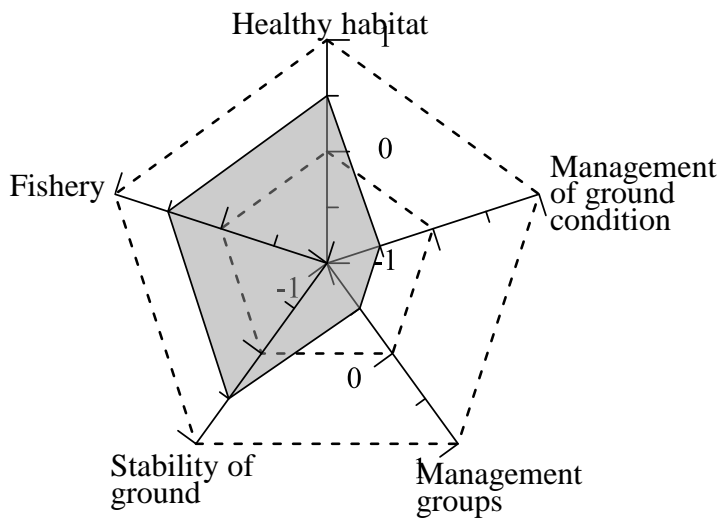

(c)

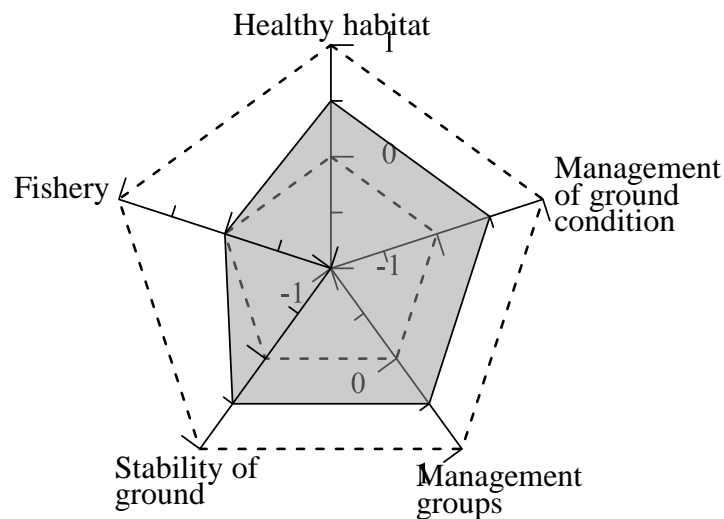

(d)

Supplement: Supplemental Information 15 — SN and UK were excluded because these types of activities and structures are not permitted in these areas. [file peerj-07-6234-s015.pdf]

## Social System

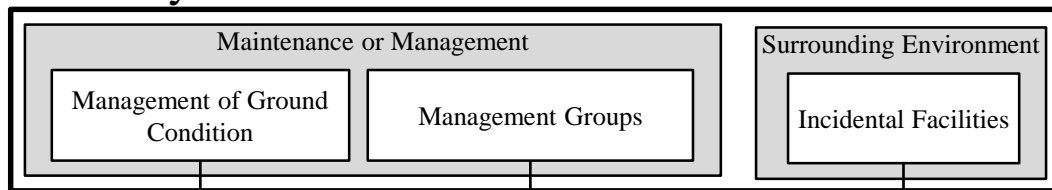

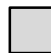 Resilience

## Natural System

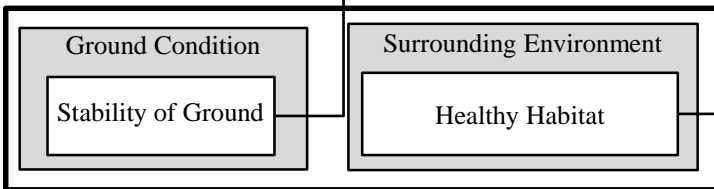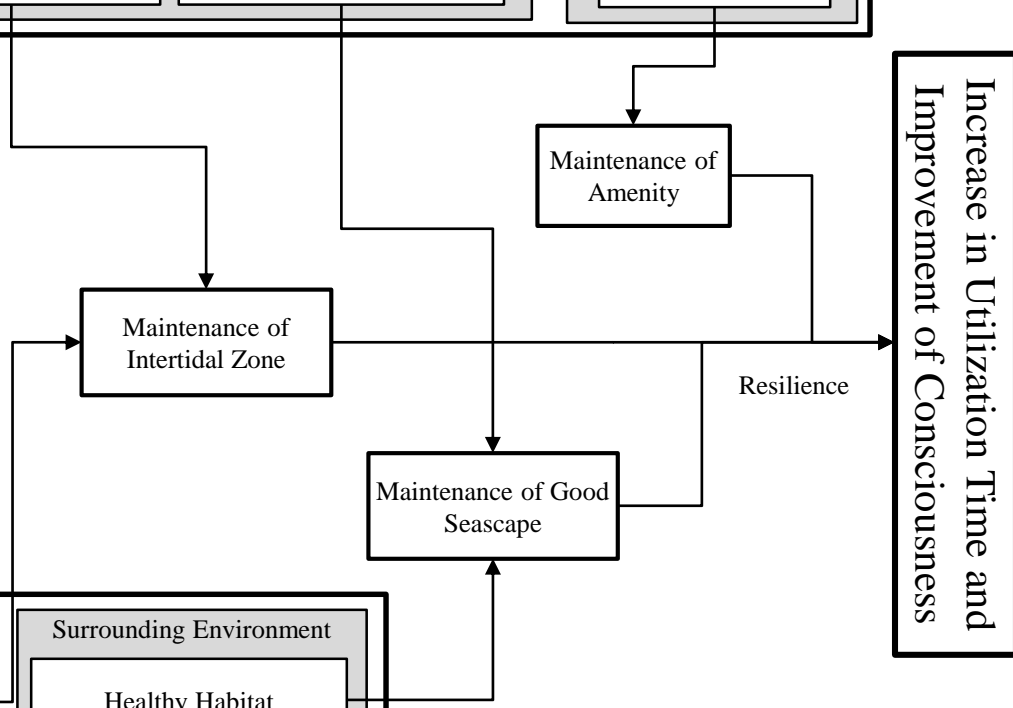

**Maintenance of Places for  
Rest and Relaxation**

Supplement: Supplemental Information 16 [file peerj-07-6234-s016.pdf]

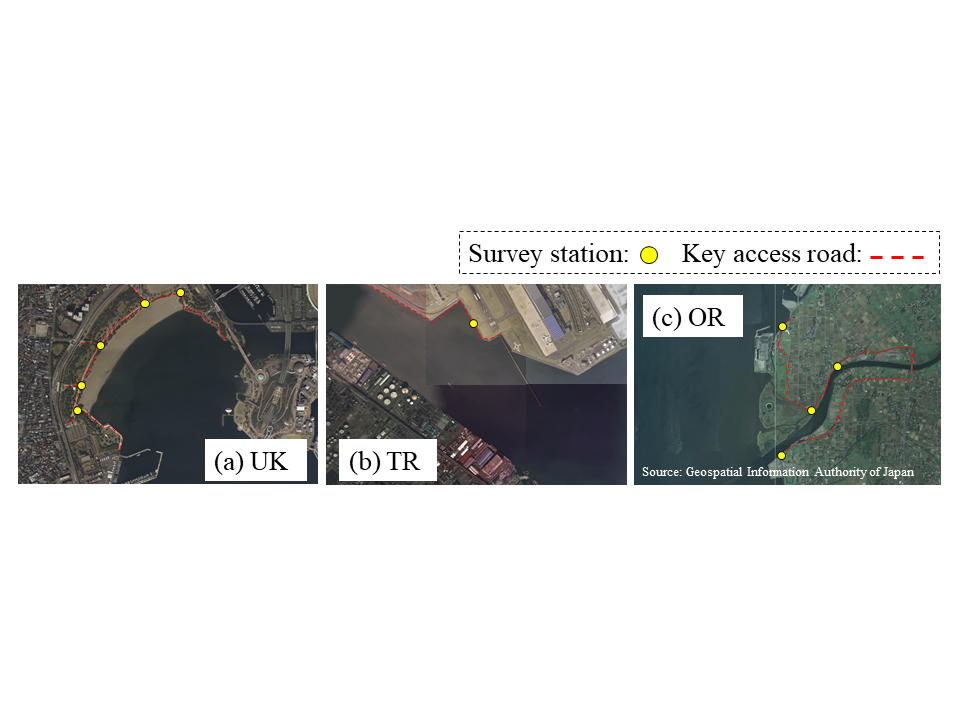

Supplement: Supplemental Information 17 — (c) source: aerial photograph of Geospatial Information Authority of Japan, https://mapps.gsi.go.jp/. [file peerj-07-6234-s017.png]

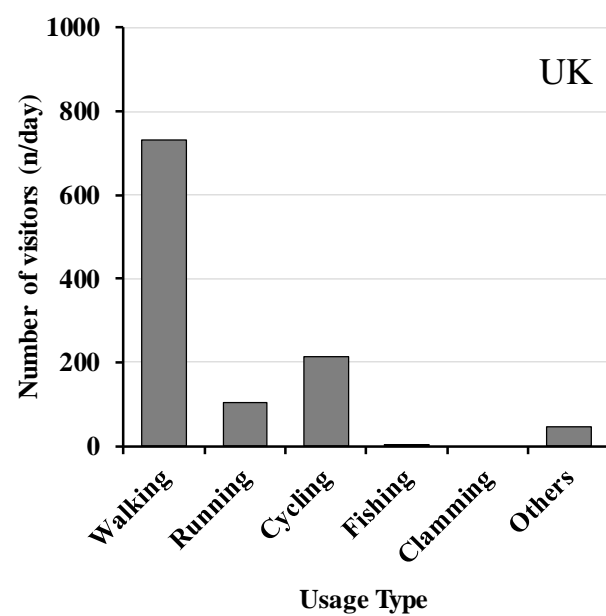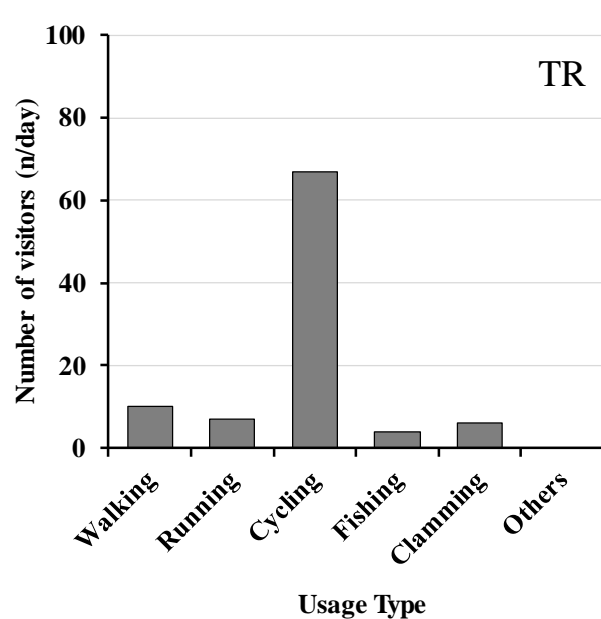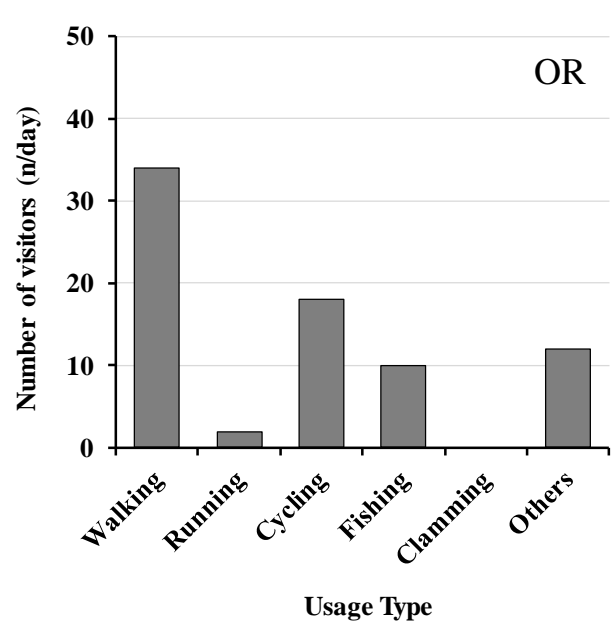

Supplement: Supplemental Information 18 [file peerj-07-6234-s018.pdf]

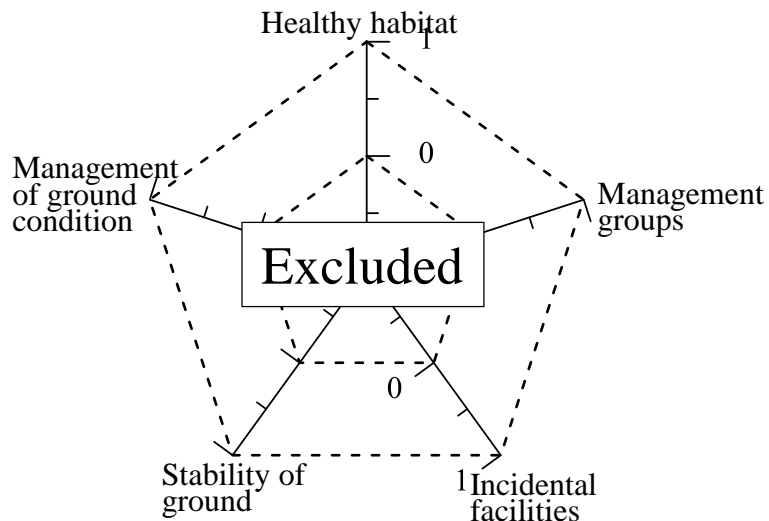

(a)

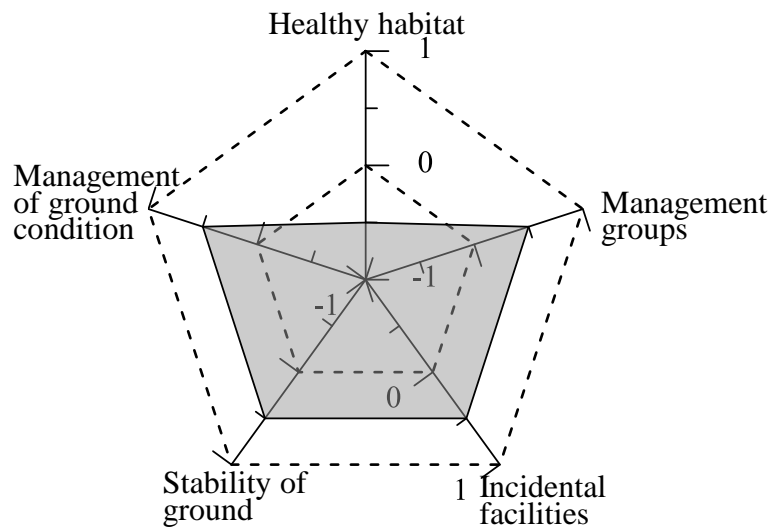

(b)

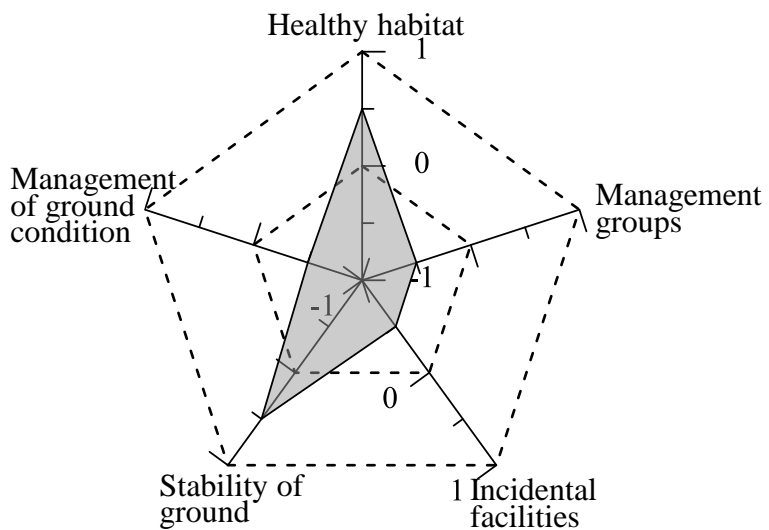

(c)

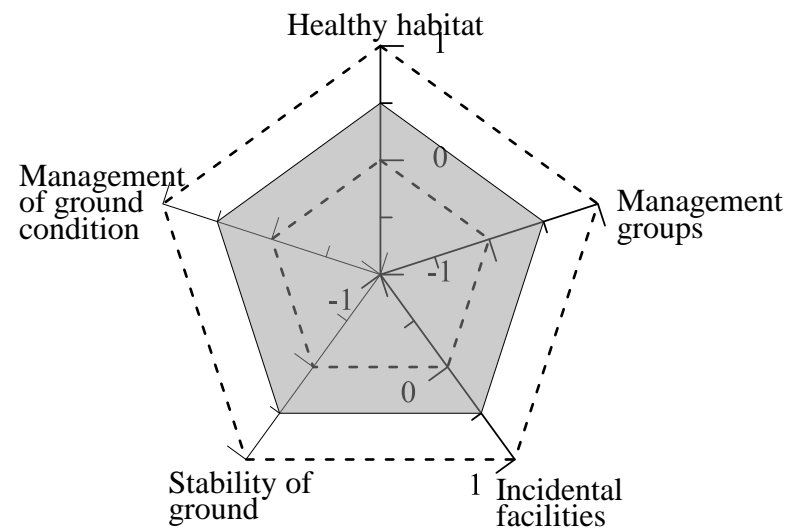

(d)

Supplement: Supplemental Information 20 — SN was excluded because these activities are not permitted in this area. [file peerj-07-6234-s020.pdf]

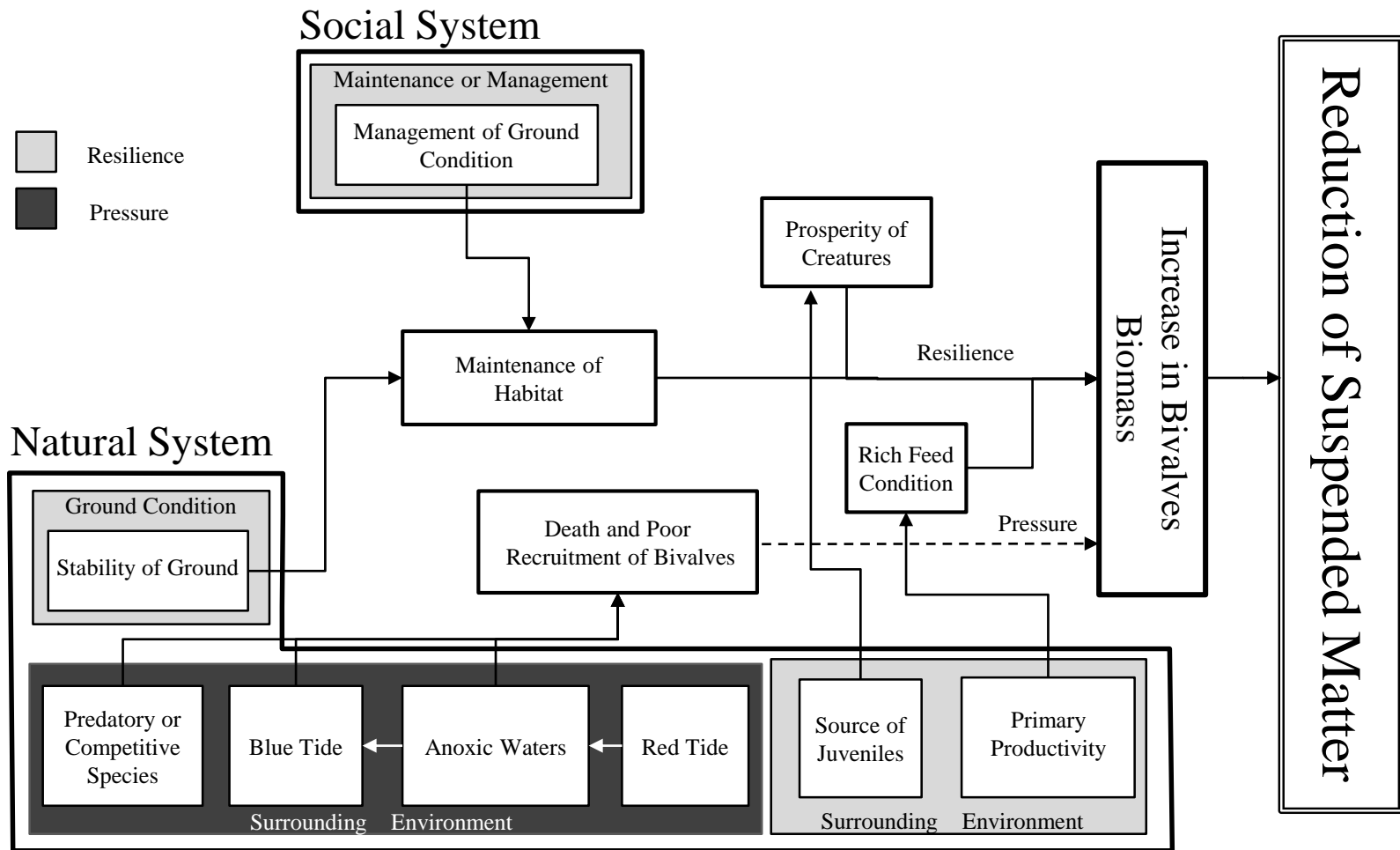

Supplement: Supplemental Information 21 [file peerj-07-6234-s021.pdf]

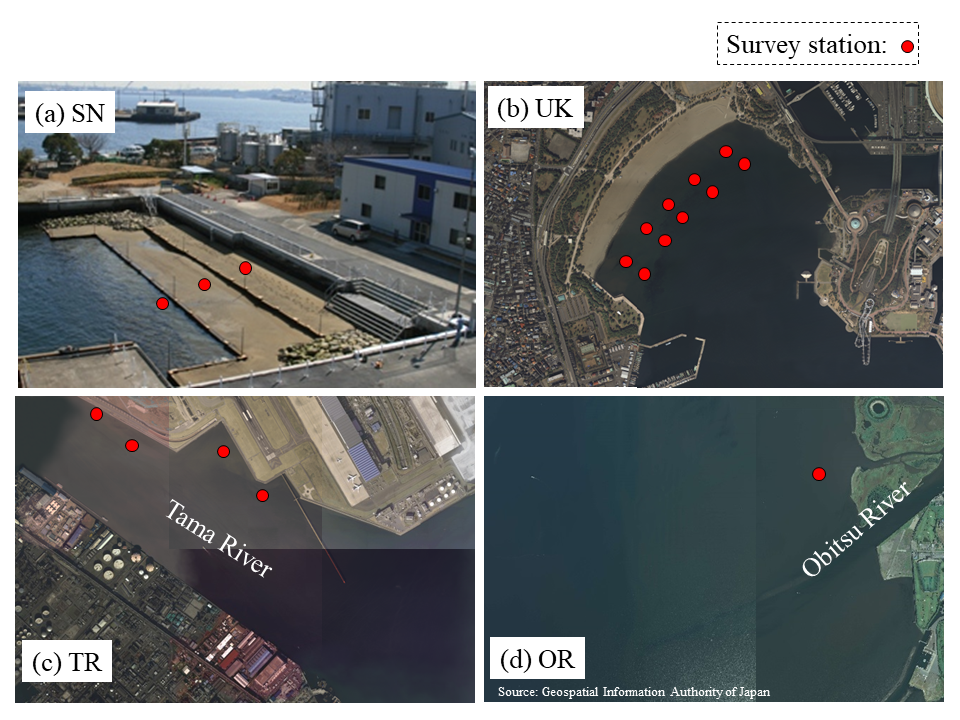

Supplement: Supplemental Information 22 — (d) source: aerial photograph of Geospatial Information Authority of Japan, https://mapps.gsi.go.jp/. [file peerj-07-6234-s022.png]

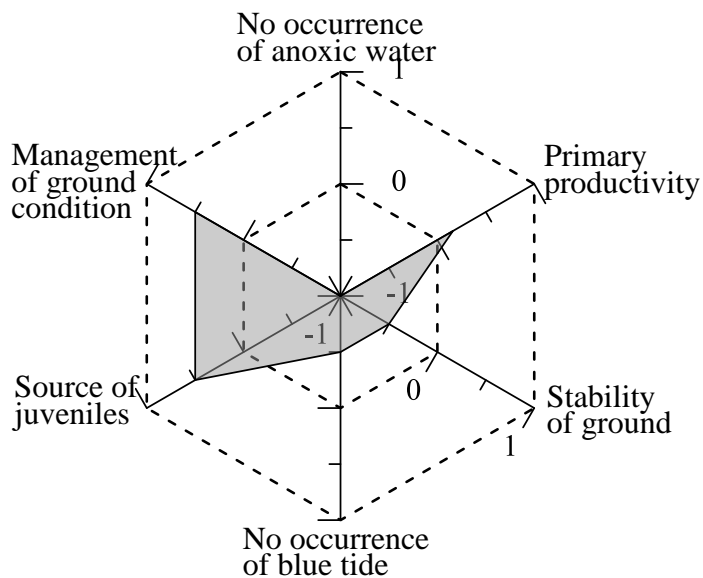

(a)

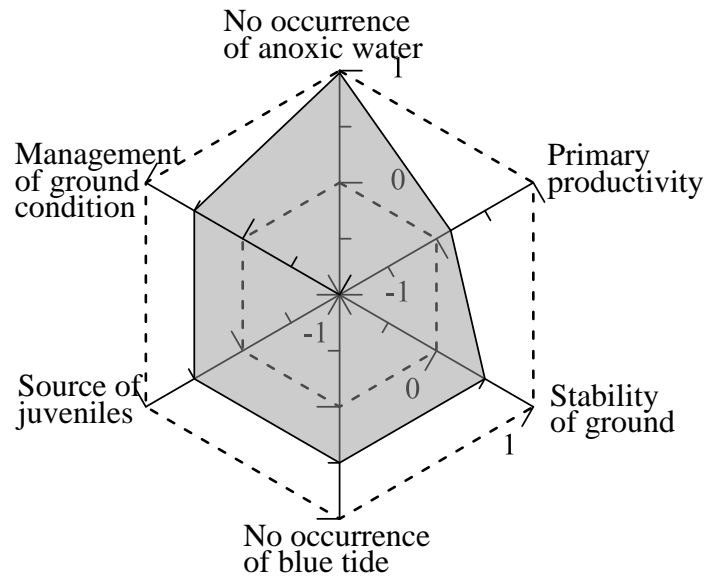

(b)

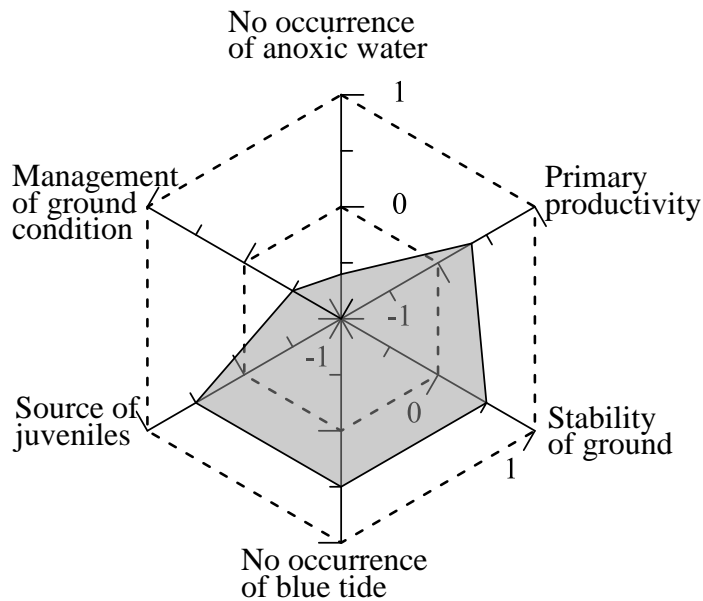

(c)

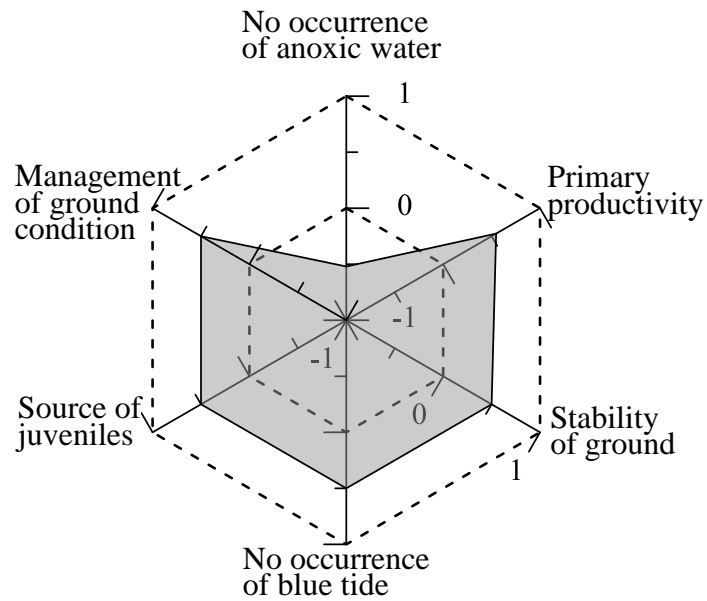

(d)

Supplement: Supplemental Information 23 [file peerj-07-6234-s023.pdf]

## Social System

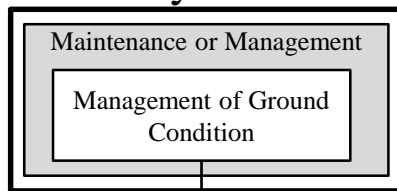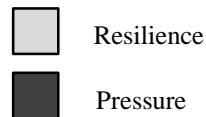

## Natural System

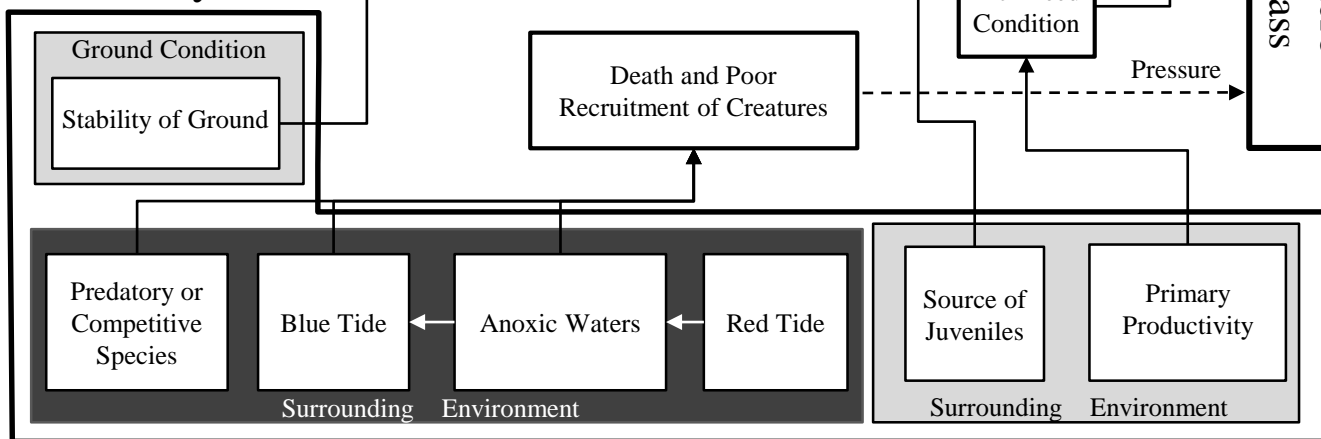

Improvement of Organic  
Purification Function

Supplement: Supplemental Information 24 [file peerj-07-6234-s024.pdf]

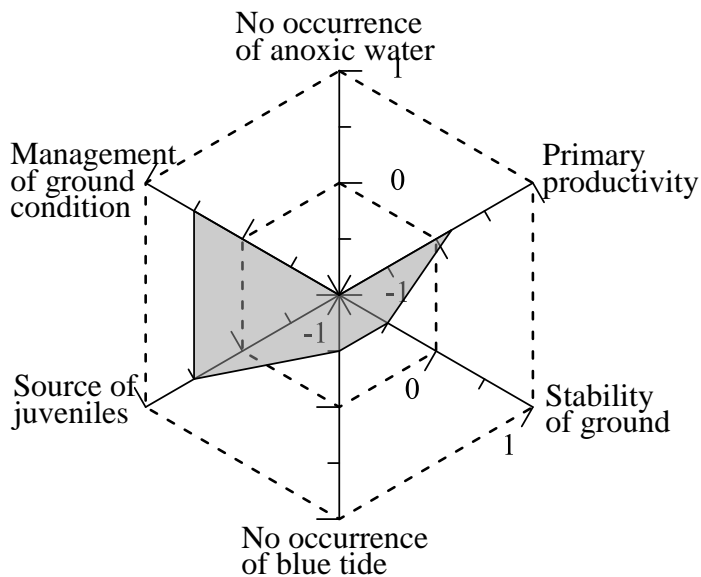

(a)

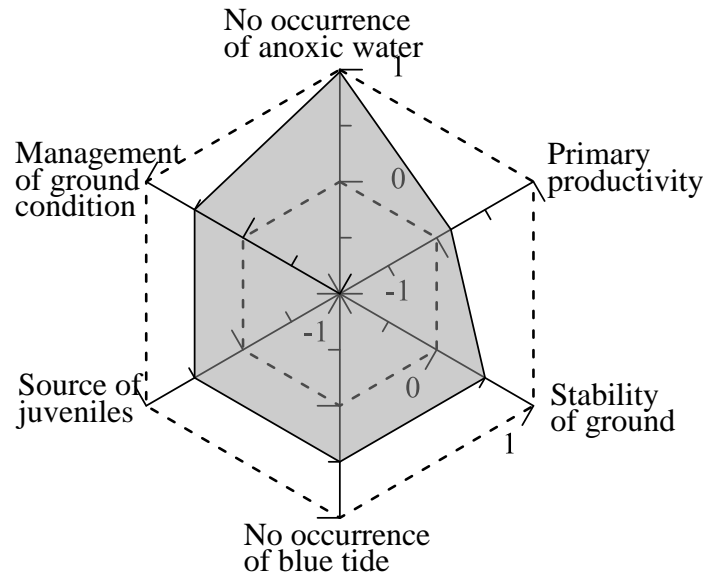

(b)

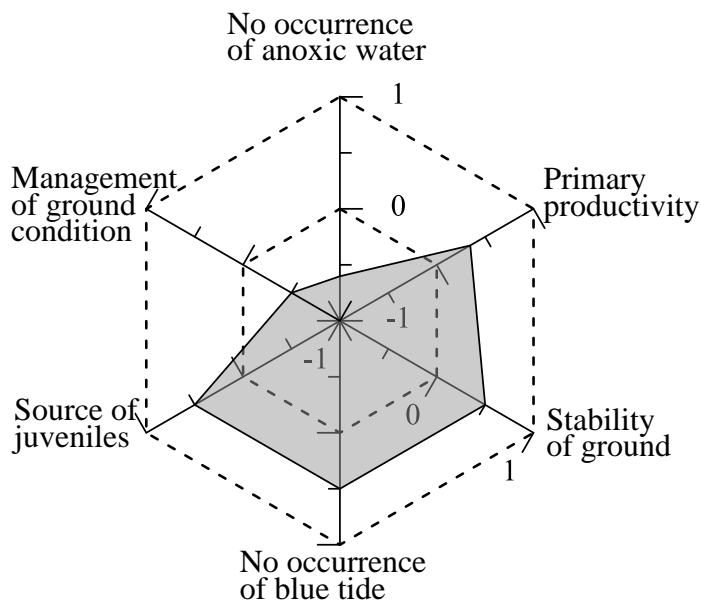

(c)

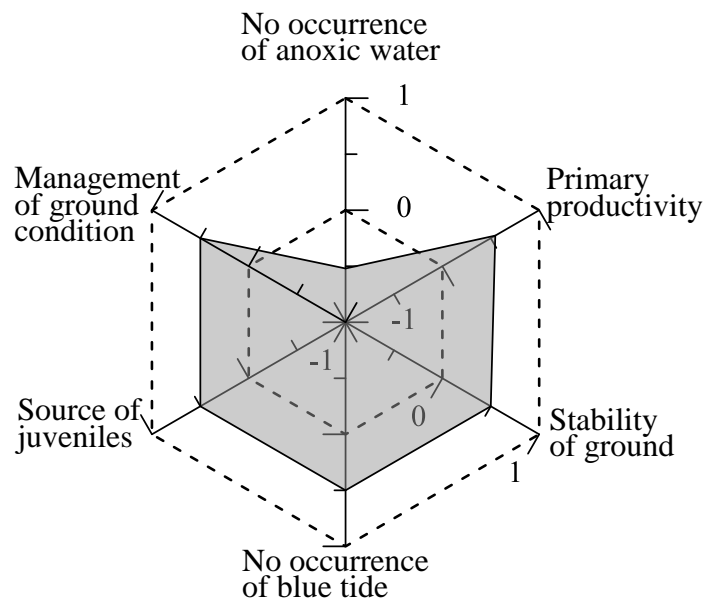

(d)

Supplement: Supplemental Information 25 [file peerj-07-6234-s025.pdf]

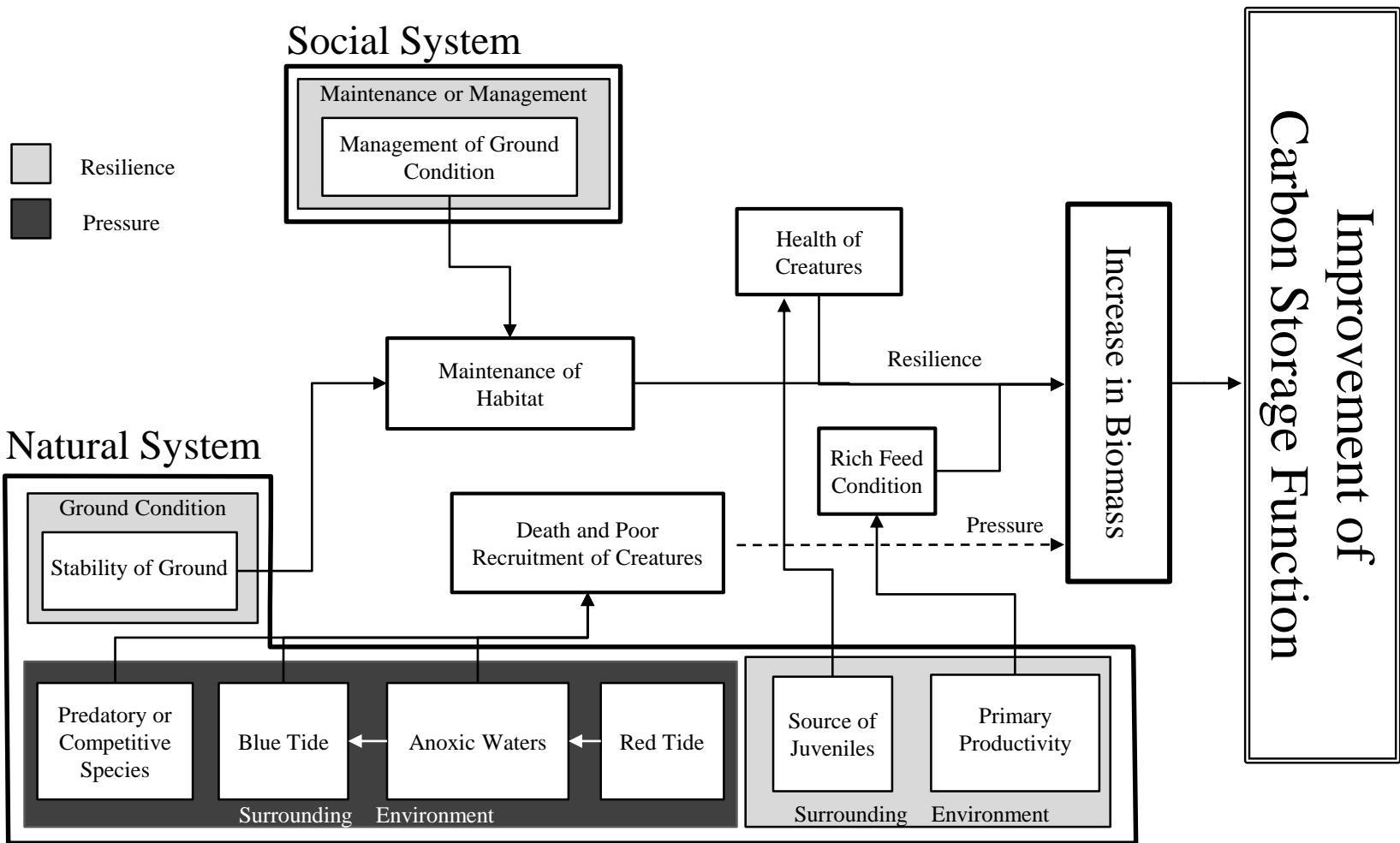

Supplement: Supplemental Information 26 [file peerj-07-6234-s026.pdf]

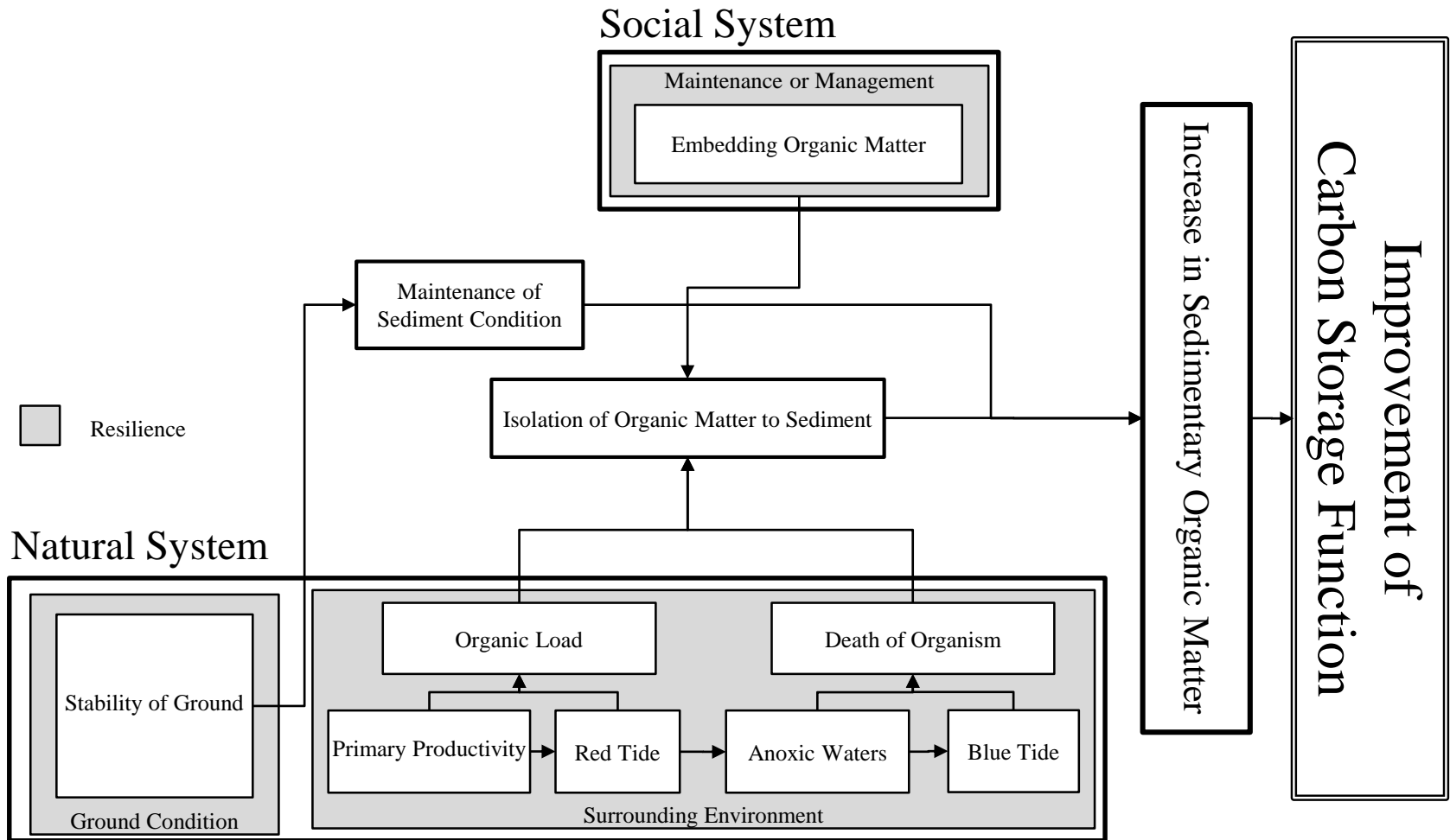

Supplement: Supplemental Information 27 [file peerj-07-6234-s027.pdf]

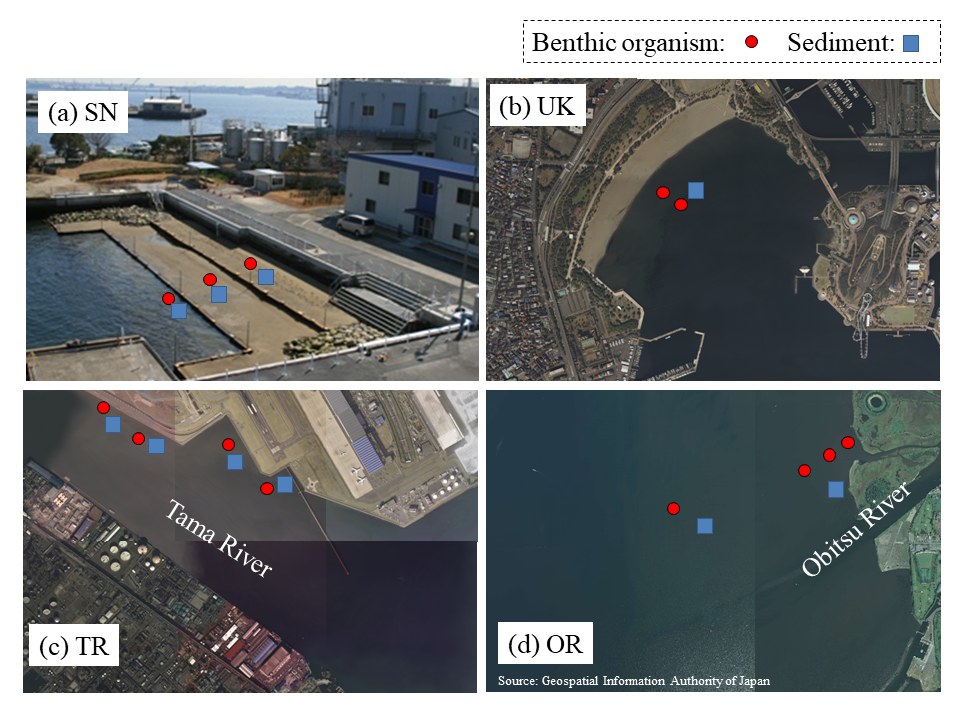

Supplement: Supplemental Information 28 — (d) source: aerial photograph of Geospatial Information Authority of Japan, https://mapps.gsi.go.jp/. [file peerj-07-6234-s028.png]

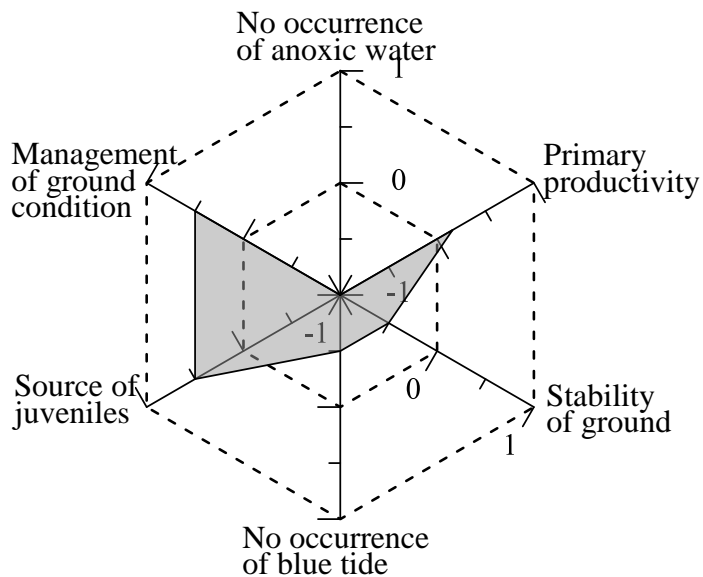

(a)

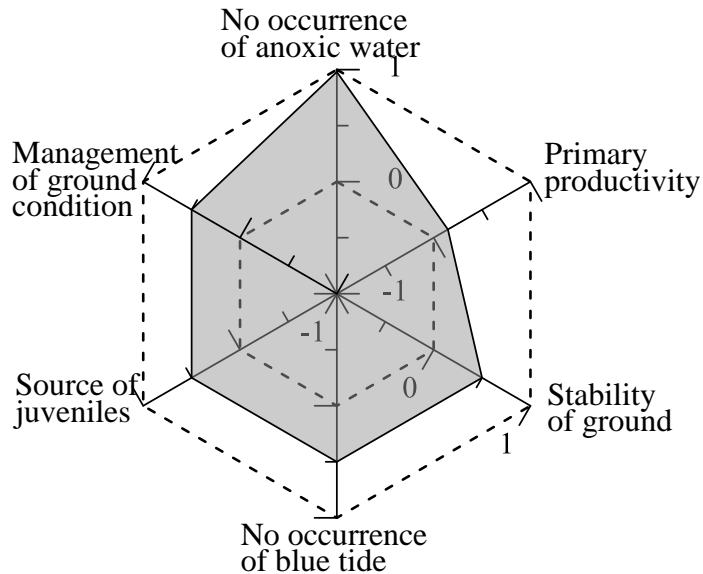

(b)

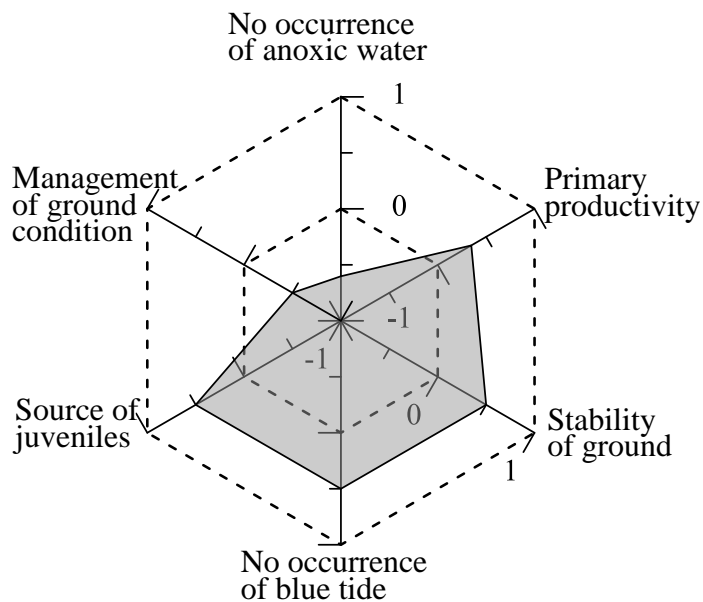

(c)

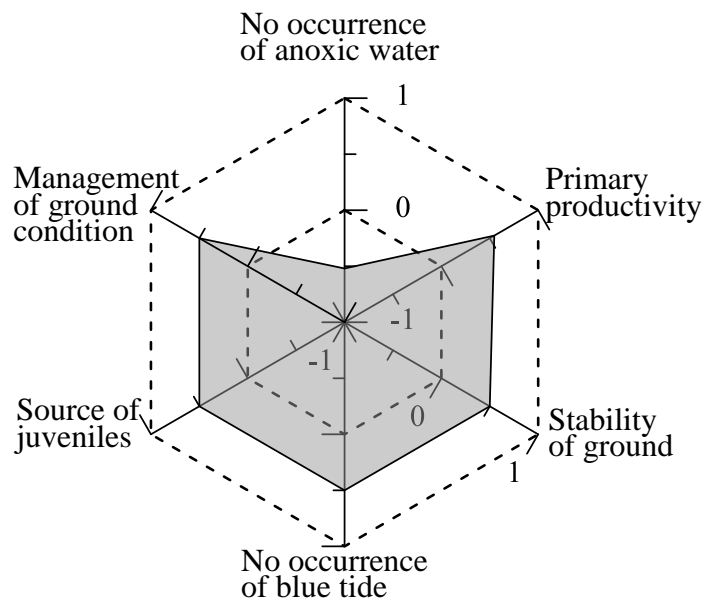

(d)

Supplement: Supplemental Information 29 [file peerj-07-6234-s029.pdf]

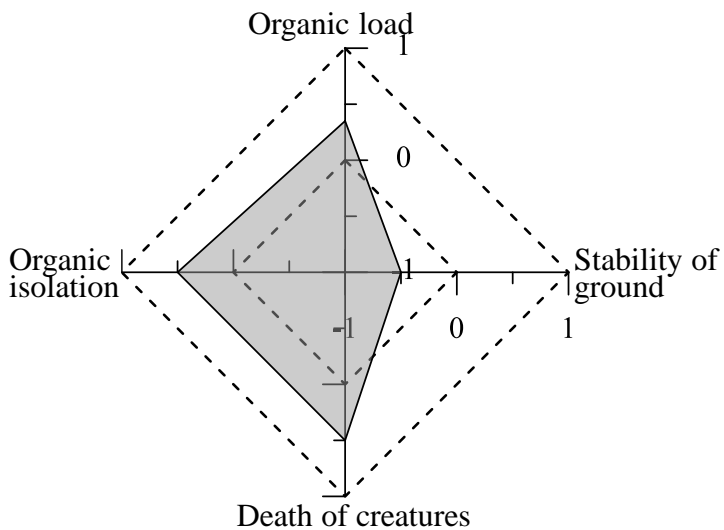

(a)

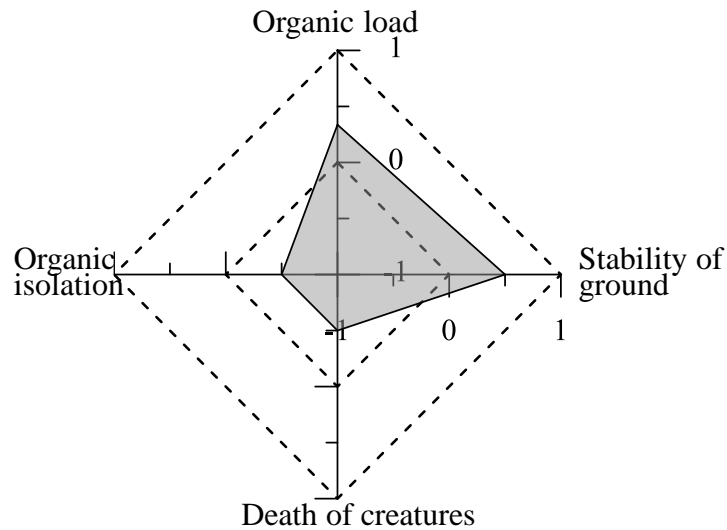

(b)

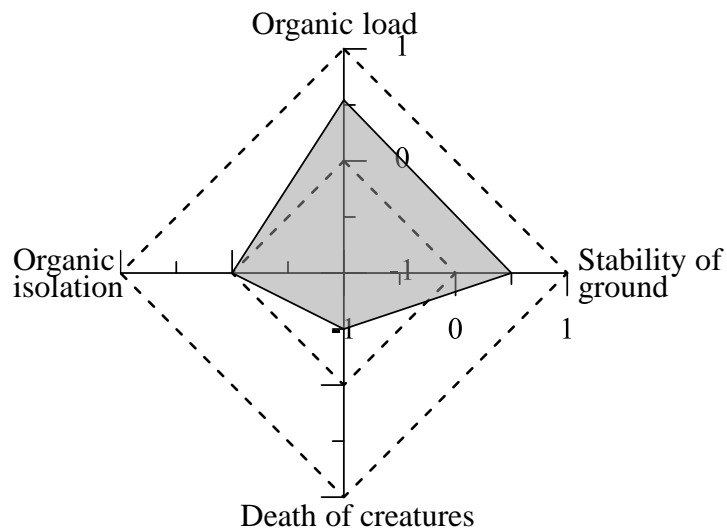

(c)

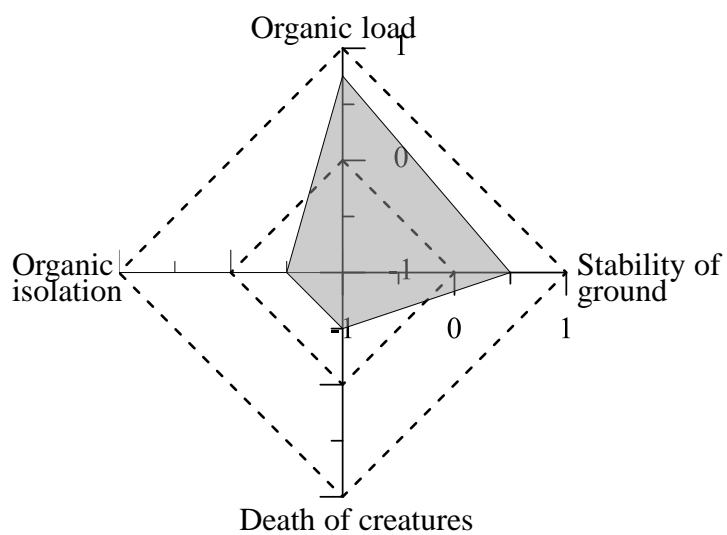

(d)

Supplement: Supplemental Information 30 [file peerj-07-6234-s030.pdf]

# Social System

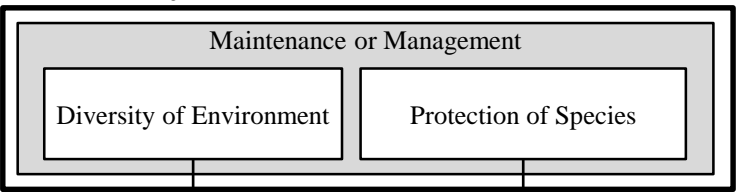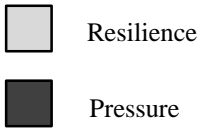

# Natural System

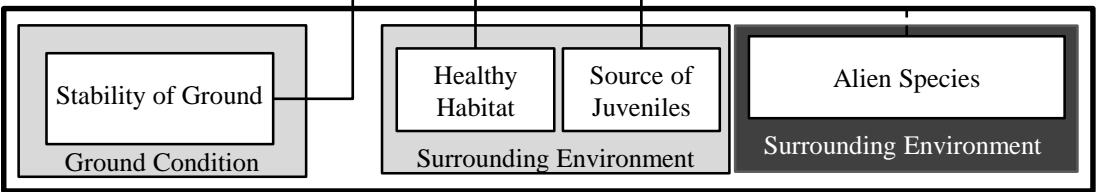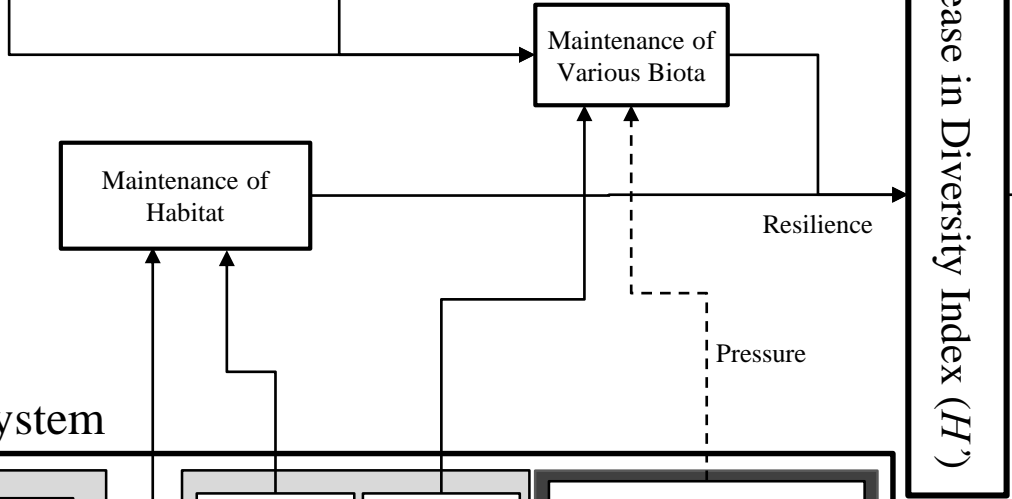

Improvement of Species Diversity

Supplement: Supplemental Information 31 [file peerj-07-6234-s031.pdf]

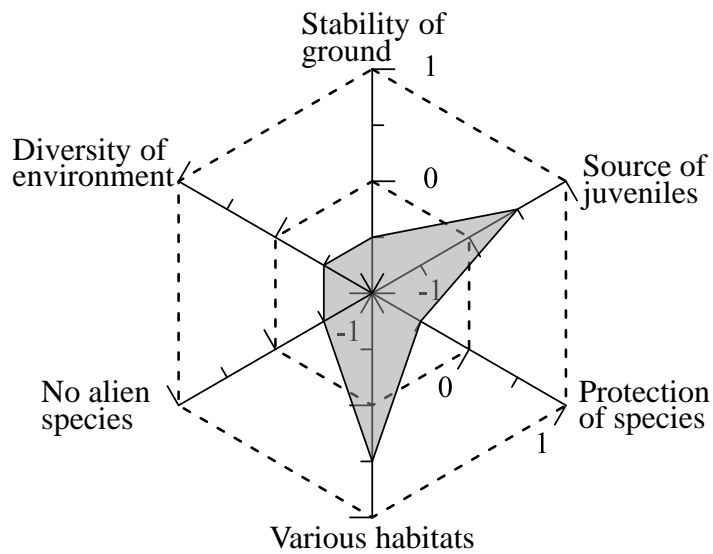

(a)

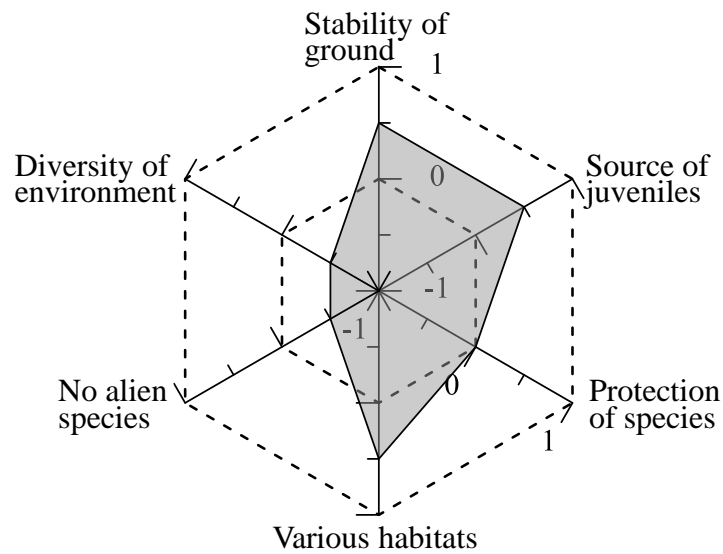

(b)

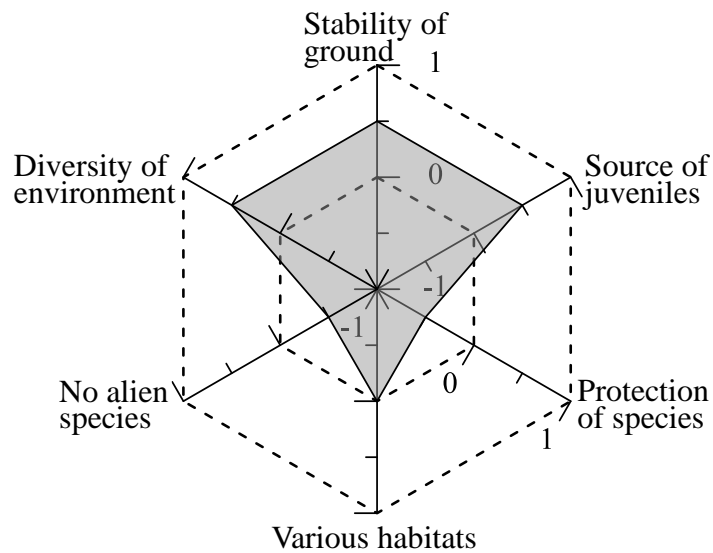

(c)

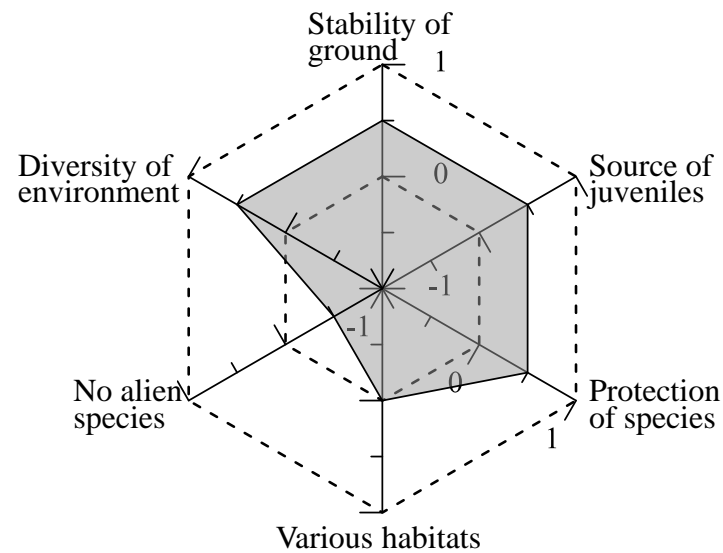

(d)

Supplement: Supplemental Information 32 [file peerj-07-6234-s032.pdf]

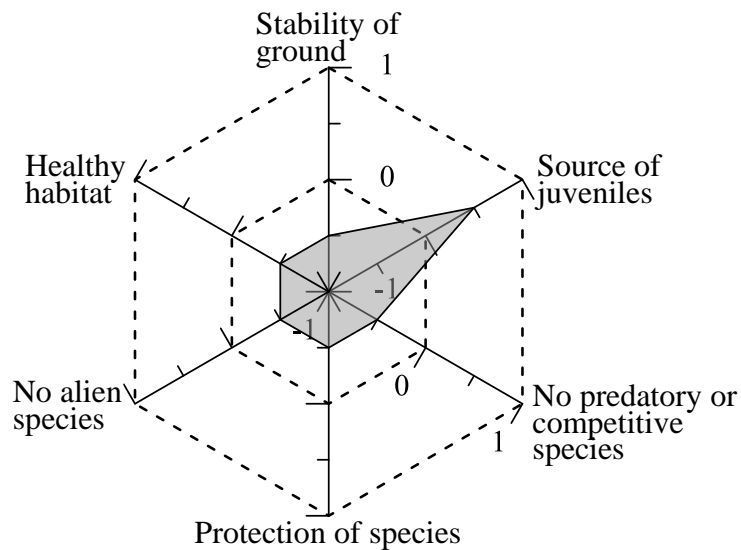

(a)

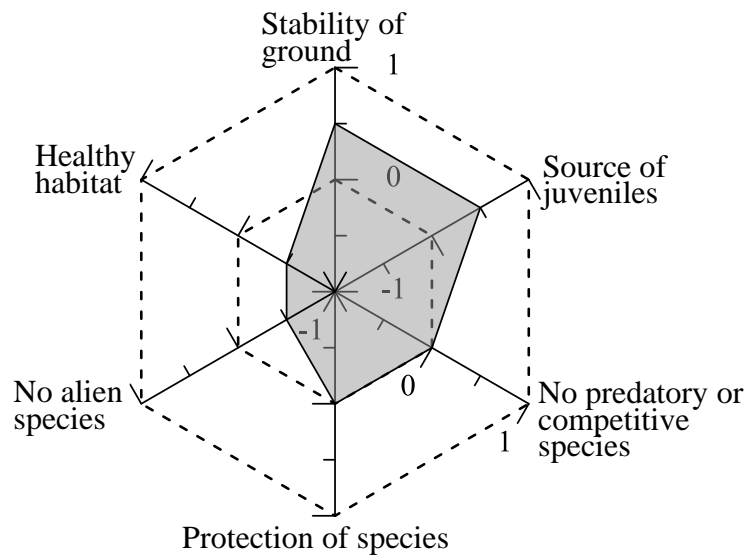

(b)

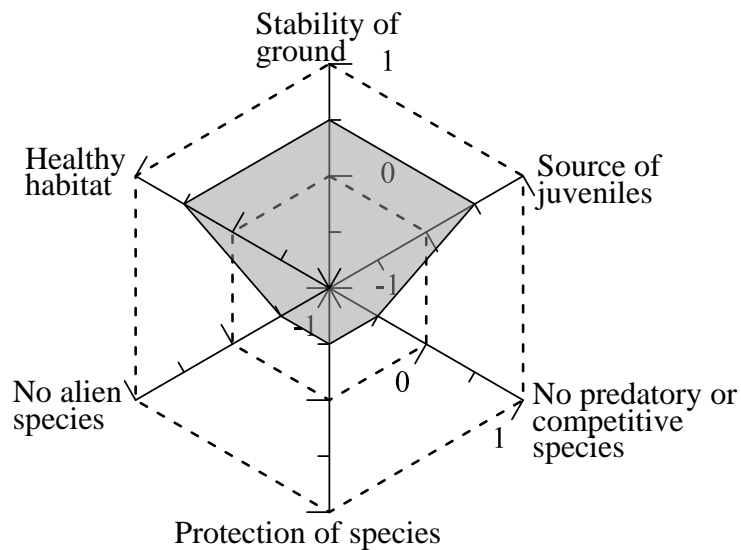

(c)

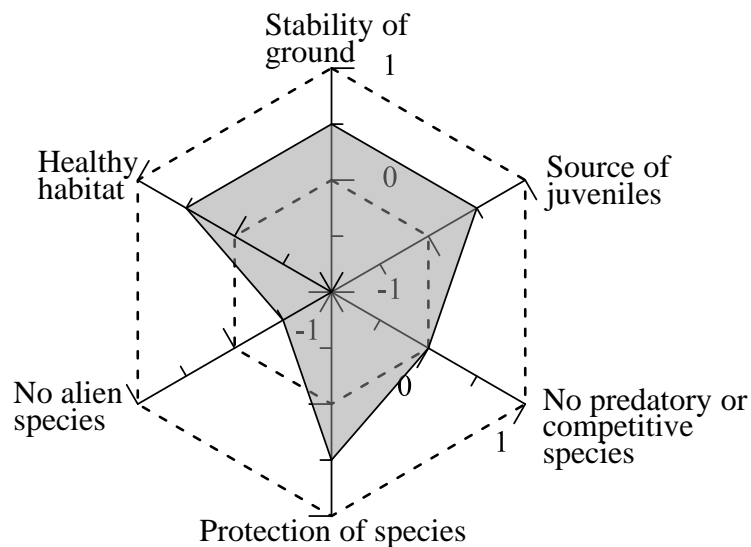

(d)

Supplement: Supplemental Information 34 [file peerj-07-6234-s034.pdf]
