# Supplementary material for: Method for the quantitative evaluation of ecosystem services in coastal regions
Source: PeerJ. 2019 Jan 14;6:e6234. doi: 10.7717/peerj.6234 (PMC6336092; doi:10.7717/peerj.6234)
Supplement: Supplemental Information 6 [file peerj-07-6234-s006.pdf]

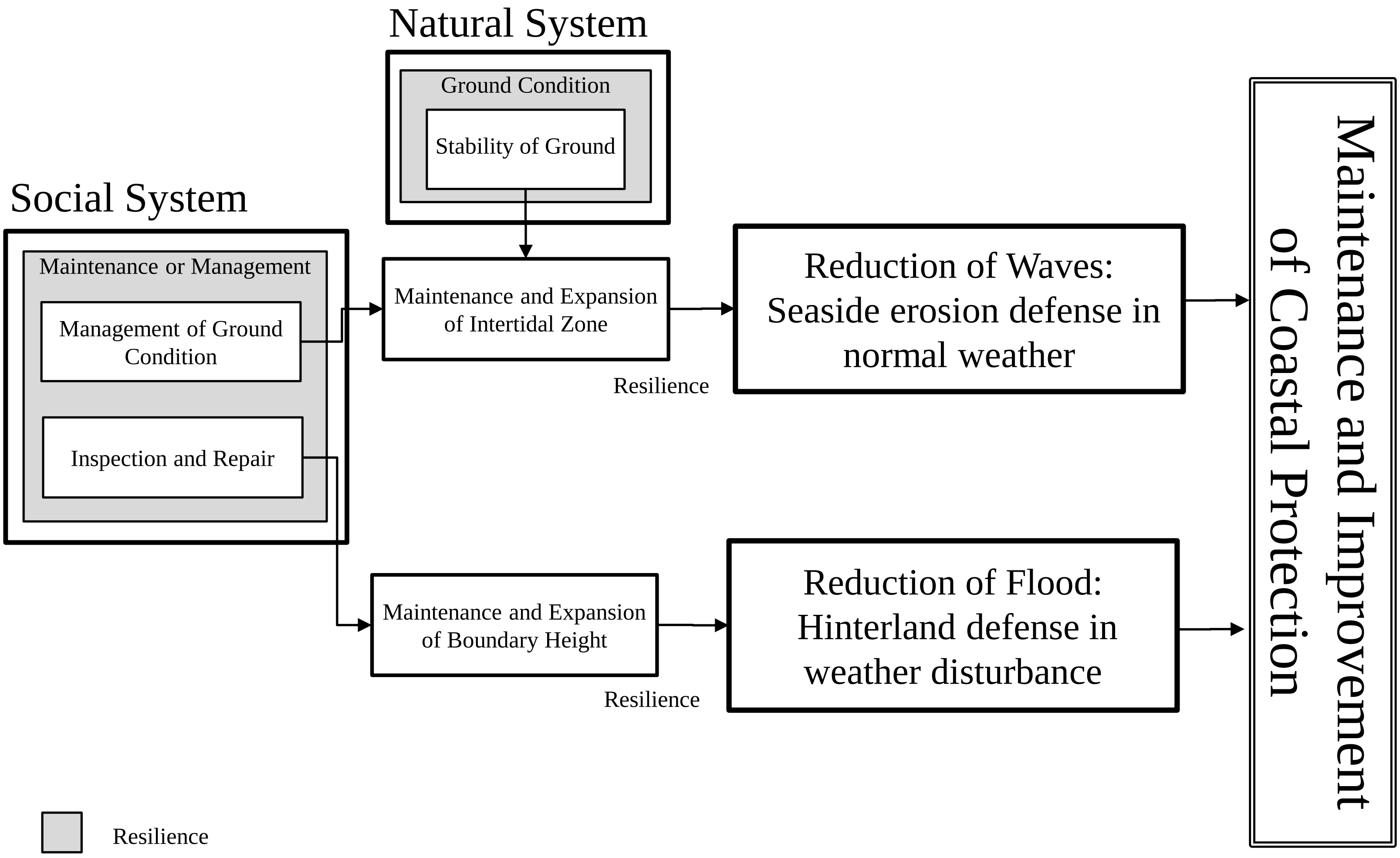
Natural System
Ground Condition
Stability of Ground
Social System
Maintenance or Management
Management of Ground Condition
Inspection and Repair
Maintenance and Expansion of Intertidal Zone
Resilience
Reduction of Waves: Seaside erosion defense in normal weather
Maintenance and Expansion of Boundary Height
Resilience
Reduction of Flood: Hinterland defense in weather disturbance
Maintenance and Improvement of Coastal Protection
Resilience
