# Supplementary material for: Method for the quantitative evaluation of ecosystem services in coastal regions
Source: PeerJ. 2019 Jan 14;6:e6234. doi: 10.7717/peerj.6234 (PMC6336092; doi:10.7717/peerj.6234)
Supplement: Supplemental Information 13 [file peerj-07-6234-s013.pdf]

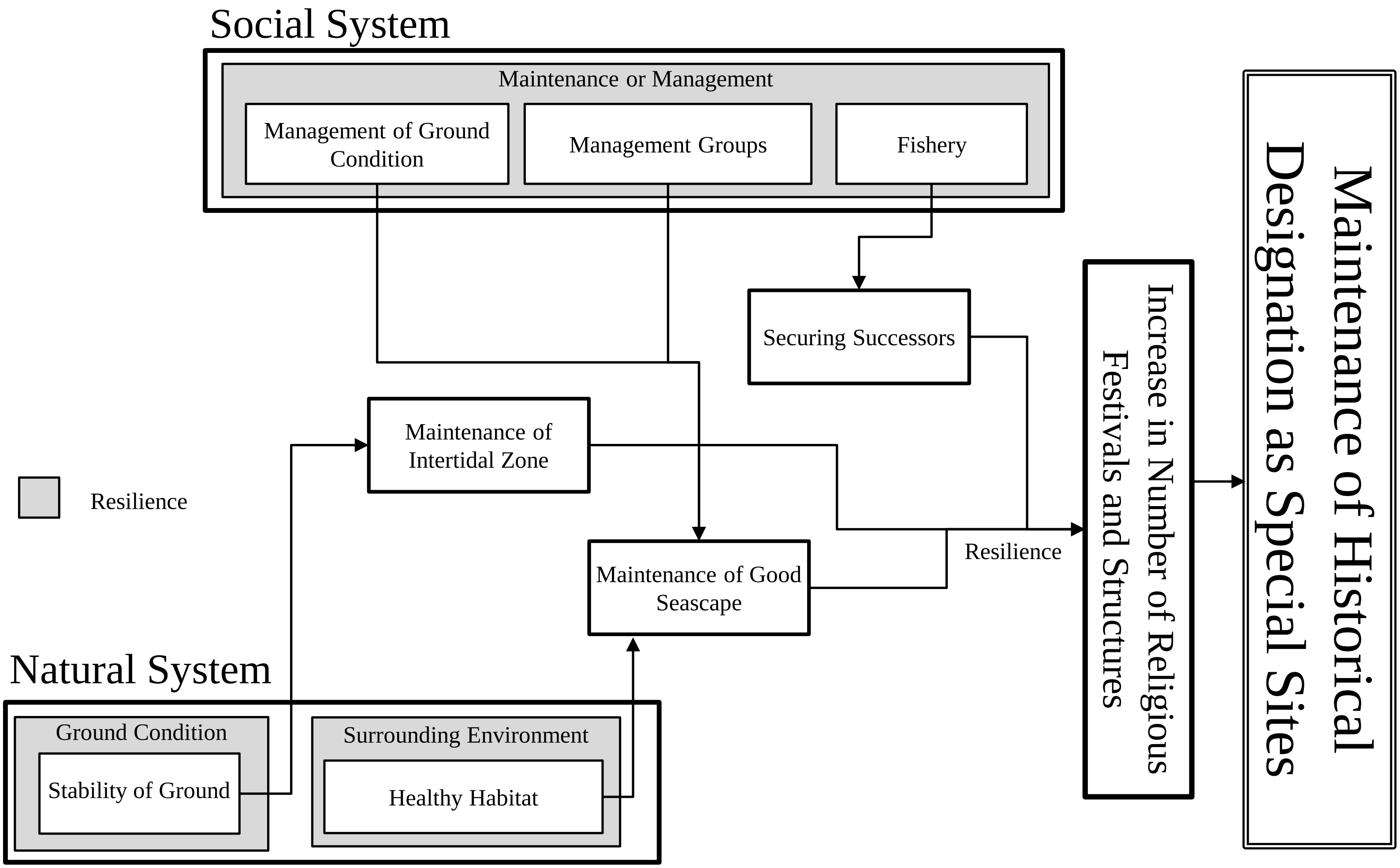

Social System
Maintenance or Management
Management of Ground Condition
Management Groups
Fishery
Securing Successors
Maintenance of Intertidal Zone
Resilience
Maintenance of Good Seascape
Resilience
Increase in Number of Religious Festivals and Structures
Maintenance of Historical Designation as Special Sites
Natural System
Ground Condition
Stability of Ground
Surrounding Environment
Healthy Habitat
