# Supplementary material for: Method for the quantitative evaluation of ecosystem services in coastal regions
Source: PeerJ. 2019 Jan 14;6:e6234. doi: 10.7717/peerj.6234 (PMC6336092; doi:10.7717/peerj.6234)
Supplement: Supplemental Information 19 [file peerj-07-6234-s019.pdf]

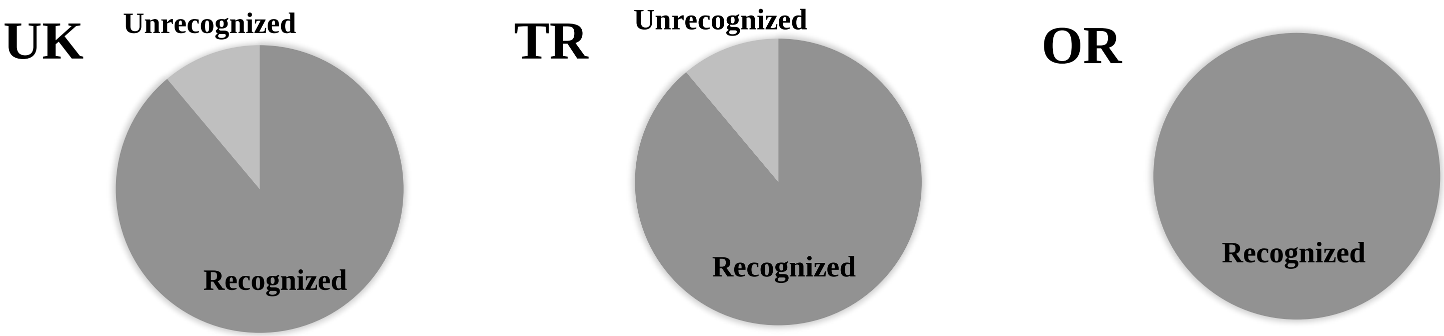

(a) Recognition of the existence of the tidal flat

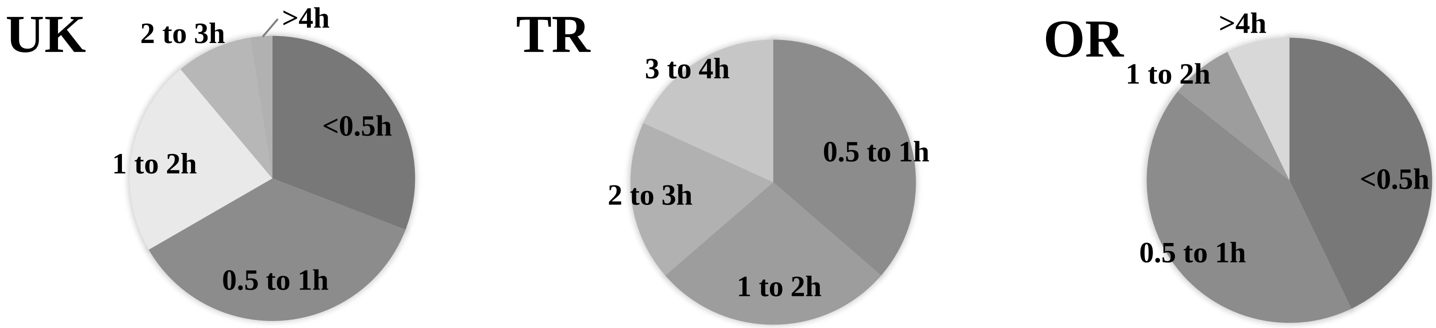

(b) Duration of stay

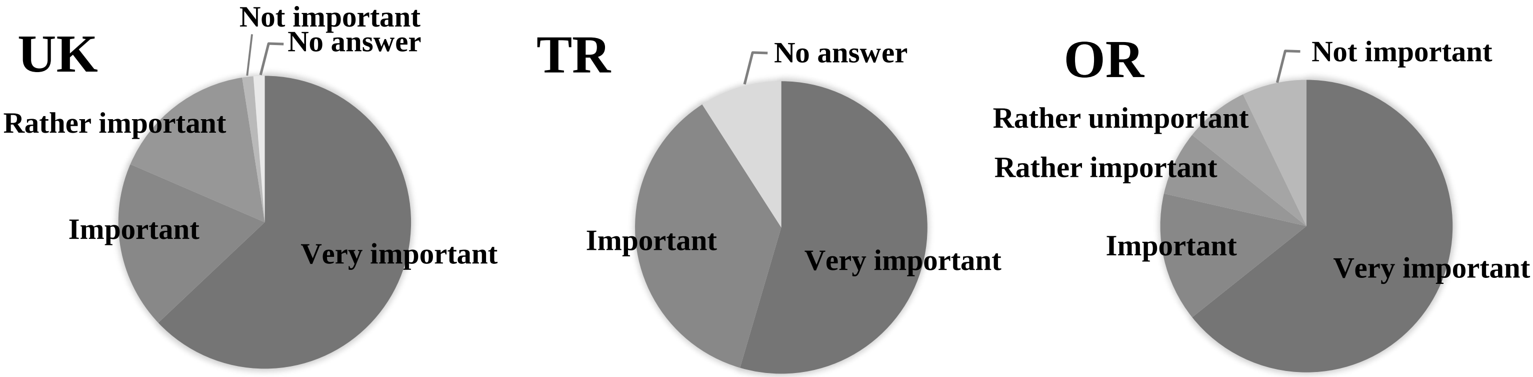

(c) Awareness of the importance of the tidal flat
