# Supplementary material for: Method for the quantitative evaluation of ecosystem services in coastal regions
Source: PeerJ. 2019 Jan 14;6:e6234. doi: 10.7717/peerj.6234 (PMC6336092; doi:10.7717/peerj.6234)
Supplement: Supplemental Information 33 [file peerj-07-6234-s033.pdf]

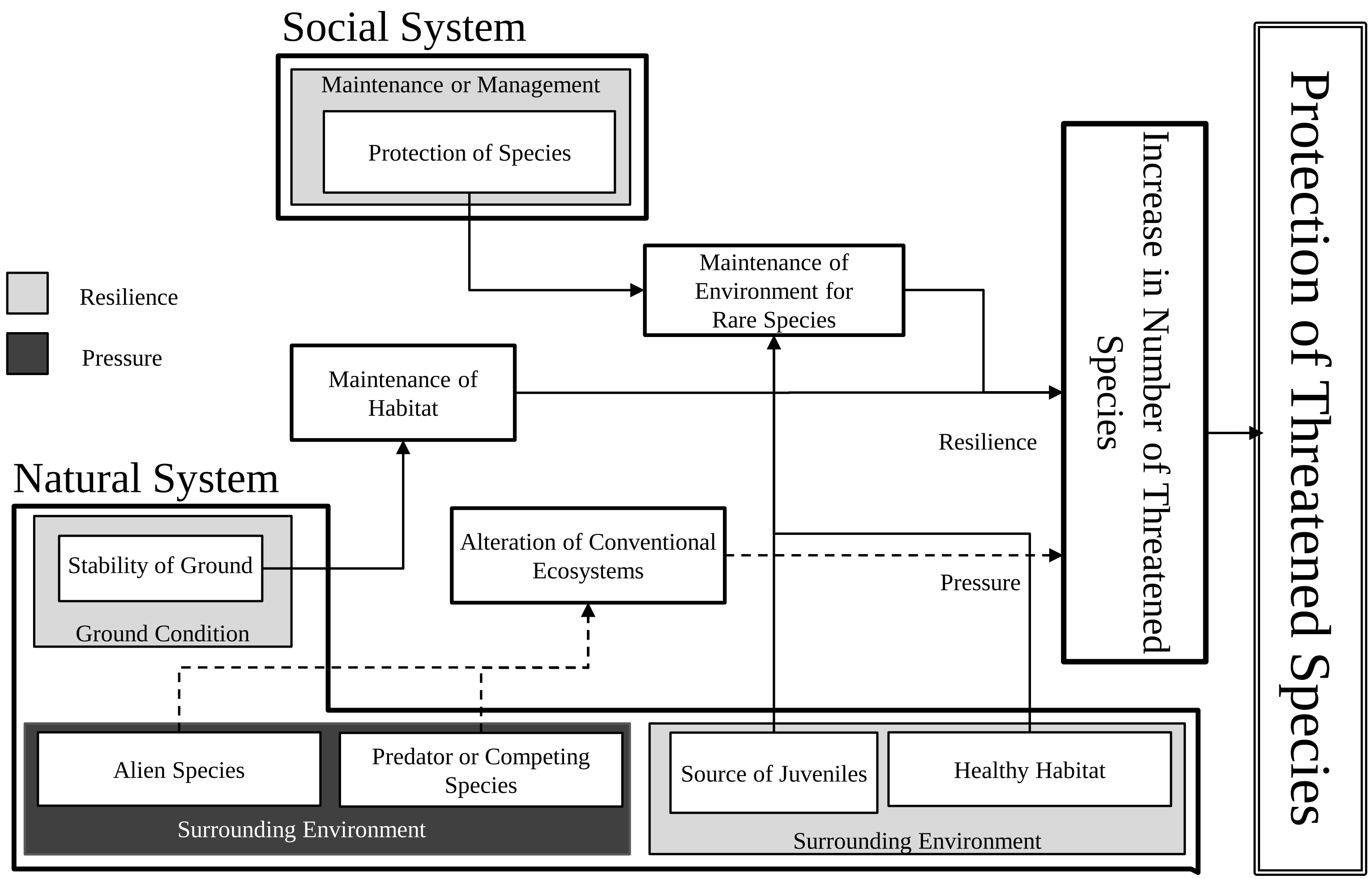

Social System
Maintenance or Management
Protection of Species
Resilience
Pressure
Maintenance of Environment for Rare Species
Maintenance of Habitat
Natural System
Stability of Ground
Ground Condition
Alteration of Conventional Ecosystems
Resilience
Pressure
Increase in Number of Threatened Species
Protection of Threatened Species
Alien Species
Predator or Competing Species
Surrounding Environment
Source of Juveniles
Healthy Habitat
Surrounding Environment
