# Supplementary material for: Method for the quantitative evaluation of ecosystem services in coastal regions
Source: PeerJ. 2019 Jan 14;6:e6234. doi: 10.7717/peerj.6234 (PMC6336092; doi:10.7717/peerj.6234)
Supplement: Supplemental Information 35 [file peerj-07-6234-s035.docx]

| Environmental factor | | Pressure or resilience condition |
| --- | --- | --- |
| Anoxic water | Resilience | DO concentration > 4 mg/L |
|  | Pressure | DO concentration < 4 mg/L |
| Blue tide | Resilience | No occurrence of blue tide |
|  | Pressure | Occurrence of blue tide |
| Predatory or competitive species | Resilience | No predatory or competitive species against commercially important species |
|  | Pressure | Existence of predatory or competitive species against commercially important species |
| Primary productivity | Resilience | Chl-a concentration > 3 μg/L |
|  | Pressure | Chl-a concentration < 3 μg/L |
| Stability of ground | Resilience | Stable ground |
|  | Pressure | Low stability of the ground (e.g., erosion, deposition, consolidation, subsidence) |
| Source of juveniles | Resilience | Existence of a nearby tidal flat |
|  | Pressure | No nearby tidal flat |
| Management of ground condition | Resilience | Existence of management of ground condition (management of fishing, seeding, control of predator or competitors, sand capping; cultivation; removal of *Ulva* sp.) |
|  | Pressure | No management of ground condition |
| Protection of species | Resilience | Existence of efforts to protect species (e.g., implementation of seedlings, control of predator or competitors) |
|  | Pressure | No efforts to protect species |
