# Supplementary material for: Method for the quantitative evaluation of ecosystem services in coastal regions
Source: PeerJ. 2019 Jan 14;6:e6234. doi: 10.7717/peerj.6234 (PMC6336092; doi:10.7717/peerj.6234)
Supplement: Supplemental Information 36 [file peerj-07-6234-s036.docx]

| Year | | 2009 | 2010 | 2011 | 2012 | 2013 |
| --- | --- | --- | --- | --- | --- | --- |
| SN | *X*_1_ | 889 | 3323 | 2175 | 773 | 518 |
|  | *x*_1_ | 0.27 | 1.00 | 0.65 | 0.23 | 0.16 |
| UK | *X*_1_ | 39 | － | － | 283 | 161 |
|  | *x*_1_ | 0.01 | － | － | 0.09 | 0.05 |
| TR | *X*_1_ | 41 | 69 | 150 | 121 | 132 |
|  | *x*_1_ | 0.01 | 0.02 | 0.05 | 0.04 | 0.04 |
| OR | *X*_1_ | 100 | 171 | 244 | 196 | 176 |
|  | *x*_1_ | 0.03 | 0.05 | 0.07 | 0.06 | 0.05 |
