# Supplementary material for: Method for the quantitative evaluation of ecosystem services in coastal regions
Source: PeerJ. 2019 Jan 14;6:e6234. doi: 10.7717/peerj.6234 (PMC6336092; doi:10.7717/peerj.6234)
Supplement: Supplemental Information 37 — Present status (x1), trend score (T1), PR score (PR1), likely near-term future status (x1,F), service score (I1), and sustainability score (S1). [file peerj-07-6234-s037.docx]

| Tidal flat | SN | UK | TR | OR |
| --- | --- | --- | --- | --- |
| *x*_1_ | 0.16 | 0.05 | 0.04 | 0.05 |
| *T*_1_ | –0.50 | 0.06 | 0.04 | 0.03 |
| *PR*_1_ | –0.23 | 0.39 | 0.03 | 0.32 |
| *x*_1,F_ | 0.09 | 0.06 | 0.04 | 0.06 |
| *I*_1_ | 12.4 | 5.3 | 4.0 | 5.6 |
| *S*_1_ | –41% | +17% | +1% | +12% |
