# Supplementary material for: Method for the quantitative evaluation of ecosystem services in coastal regions
Source: PeerJ. 2019 Jan 14;6:e6234. doi: 10.7717/peerj.6234 (PMC6336092; doi:10.7717/peerj.6234)
Supplement: Supplemental Information 38 [file peerj-07-6234-s038.docx]

| Environmental factor | | Condition of pressure or resilience |
| --- | --- | --- |
| Stability of ground | Resilience | No erosion, floating sand, subsidence |
|  | Pressure | Presence of erosion, floating sand, subsidence |
| Management of ground condition | Resilience | Implementation of beach nourishment, leveling |
|  | Pressure | Absence of these practices |
| Inspection and repair | Resilience | Implementation of appropriate inspection and repair of seawall |
|  | Pressure | Absence of inspection and repair of seawall |
