# Supplementary material for: Method for the quantitative evaluation of ecosystem services in coastal regions
Source: PeerJ. 2019 Jan 14;6:e6234. doi: 10.7717/peerj.6234 (PMC6336092; doi:10.7717/peerj.6234)
Supplement: Supplemental Information 39 [file peerj-07-6234-s039.docx]

| Year | | 2009 | 2010 | 2011 | 2012 | 2013 |
| --- | --- | --- | --- | --- | --- | --- |
| SN | *X*_2.1_ | 0.75 | 0.75 | 0.75 | 0.75 | 0.75 |
|  | *x*_2.1_ | 0.77 | 0.77 | 0.77 | 0.77 | 0.77 |
| UK | *X*_2.1_ | 0.82 | 0.82 | 0.82 | 0.82 | 0.81 |
|  | *x*_2.1_ | 0.85 | 0.85 | 0.85 | 0.85 | 0.81 |
| TR | *X*_2.1_ | 0.95 | 0.96 | 0.95 | 0.95 | 0.93 |
|  | *x*_2.1_ | 0.98 | 0.98 | 0.98 | 0.98 | 0.98 |
| OR | *X*_2.1_ | 0.97 | 0.97 | 0.96 | 0.96 | 0.97 |
|  | *x*_2.1_ | 1.00 | 1.00 | 1.00 | 1.00 | 1.00 |
