# Supplementary material for: Method for the quantitative evaluation of ecosystem services in coastal regions
Source: PeerJ. 2019 Jan 14;6:e6234. doi: 10.7717/peerj.6234 (PMC6336092; doi:10.7717/peerj.6234)
Supplement: Supplemental Information 40 [file peerj-07-6234-s040.docx]

| Site | Wave height  *H*_0_ (m) | Crushing depth  *H_b_* (m) | Run-up height  *R* (m) | Boundary height  *hc* (m) | *X*_2.2_  (1 – *R*/*hc*) |
| --- | --- | --- | --- | --- | --- |
| SN | 0.250 | 0.37 | 0.25 | 3.0 | 0.75 |
| UK | 0.448 | 0.66 | 0.14 | 3.0 | 0.86 |
| TR | 1.38 | 2.0 | 0.66 | 4.2 | 0.68 |
| OR | 2.03 | 3.0 | 0.58 | 4.4 | 0.75 |
