# Supplementary material for: Method for the quantitative evaluation of ecosystem services in coastal regions
Source: PeerJ. 2019 Jan 14;6:e6234. doi: 10.7717/peerj.6234 (PMC6336092; doi:10.7717/peerj.6234)
Supplement: Supplemental Information 42 [file peerj-07-6234-s042.docx]

| Year | 2009 | 2010 | 2011 | 2012 | 2013 |
| --- | --- | --- | --- | --- | --- |
| SN | 0.81 | 0.81 | 0.81 | 0.81 | 0.81 |
| UK | 0.90 | 0.87 | 0.87 | 0.92 | 0.91 |
| TR | 0.92 | 0.94 | 0.94 | 0.93 | 0.87 |
| OR | 0.94 | 0.93 | 0.90 | 0.90 | 0.92 |
