# Supplementary material for: Method for the quantitative evaluation of ecosystem services in coastal regions
Source: PeerJ. 2019 Jan 14;6:e6234. doi: 10.7717/peerj.6234 (PMC6336092; doi:10.7717/peerj.6234)
Supplement: Supplemental Information 43 — Present status (x2), trend score (T2), PR score (PR2), likely near-term future status (x2,F), service score (I2), and sustainability score (S2). [file peerj-07-6234-s043.docx]

| Tidal flat | SN | UK | TR | OR |
| --- | --- | --- | --- | --- |
| *x*_2_ | 0.81 | 0.91 | 0.87 | 0.92 |
| *T*_2_ | 0.00 | 0.03 | –0.06 | –0.02 |
| *PR*_2_ | 0.00 | 0.33 | 0.00 | 0.17 |
| *x*_2,F_ | 0.81 | 1.02 | 0.83 | 0.96 |
| *I*_2_ | 81.2 | 96.1 | 85.1 | 94.3 |
| *S*_2,_ | 0% | +13% | –4% | +4% |
