# Supplementary material for: Method for the quantitative evaluation of ecosystem services in coastal regions
Source: PeerJ. 2019 Jan 14;6:e6234. doi: 10.7717/peerj.6234 (PMC6336092; doi:10.7717/peerj.6234)
Supplement: Supplemental Information 44 [file peerj-07-6234-s044.docx]

| Environmental factor | | Condition of pressure or resilience |
| --- | --- | --- |
| Healthy habitat | Resilience | No occurrence of odor due to blue tide or other organisms, mass death of organisms, or outbreak of specific species (*Ulva.*sp) |
|  | Pressure | Occurrence of them |
| Stability of ground | Resilience | No erosion or subsidence |
|  | Pressure | Occurrence of erosion or subsidence |
| Management groups | Resilience | Presence of management groups about amenities |
|  | Pressure | Absence of them |
| Attracting visitors | Resilience | Presence of sports, fishing, or clamming events |
|  | Pressure | Absence of them |
| Publicity work | Resilience | Presence of public relations via the web and distribution of advertisements |
|  | Pressure | Absence of them |
| Incidental facilities | Resilience | Presence of rest huts, public toilets, event rooms, etc. |
|  | Pressure | Absence of them |
| Accessibility | Resilience | Presence of public transport station or parking |
|  | Pressure | Lack of them |
