# Supplementary material for: Method for the quantitative evaluation of ecosystem services in coastal regions
Source: PeerJ. 2019 Jan 14;6:e6234. doi: 10.7717/peerj.6234 (PMC6336092; doi:10.7717/peerj.6234)
Supplement: Supplemental Information 45 [file peerj-07-6234-s045.docx]

| Year | | 2009 | 2010 | 2011 | 2012 | 2013 |
| --- | --- | --- | --- | --- | --- | --- |
| SN | *X*_3_ | － | － | － | － | － |
|  | *x*_3_ | － | － | － | － | － |
| UK | *X*_3_ | 2220 | 1748 | 1474 | 1844 | 1755 |
|  | *x*_3_ | 1.00 | 0.79 | 0.66 | 0.83 | 0.79 |
| TR | *X*_3_ | 6 | － | － | － | 3 |
|  | *x*_3_ | 0.00 | － | － | － | 0.00 |
| OR | *X*_3_ | 83 | 100 | 37 | 46 | 63 |
|  | *x*_3_ | 0.04 | 0.05 | 0.02 | 0.02 | 0.03 |
