# Supplementary material for: Method for the quantitative evaluation of ecosystem services in coastal regions
Source: PeerJ. 2019 Jan 14;6:e6234. doi: 10.7717/peerj.6234 (PMC6336092; doi:10.7717/peerj.6234)
Supplement: Supplemental Information 46 — Present status (x3), trend score (T3), PR score (PR3), likely near-term future status (x3,F), service score (I3), and sustainability score (S3). [file peerj-07-6234-s046.docx]

| Tidal flat | SN | UK | TR | OR |
| --- | --- | --- | --- | --- |
| *x*_3_ | － | 0.79 | 0.00 | 0.00 |
| *T*_3_ | － | –0.19 | 0.00 | 0.00 |
| *PR*_3_ | － | 0.36 | –0.21 | 0.50 |
| *x*_3,F_ | － | 0.78 | 0.00 | 0.00 |
| *I*_3_ | － | 78.7 | 0.3 | 0.3 |
| *S*_3_ | － | –1% | –7% | +16% |

Note: SN was excluded because recreation is not allowed in this area.
