# Supplementary material for: Method for the quantitative evaluation of ecosystem services in coastal regions
Source: PeerJ. 2019 Jan 14;6:e6234. doi: 10.7717/peerj.6234 (PMC6336092; doi:10.7717/peerj.6234)
Supplement: Supplemental Information 47 [file peerj-07-6234-s047.docx]

| Environmental factor | | Condition of pressure or resilience |
| --- | --- | --- |
| Healthy habitat | Resilience | No occurrence of odor due to blue tide or other organisms, mass death of organisms, or outbreak of specific species (*Ulva* sp.) |
|  | Pressure | Occurrence of them |
| Stability of ground | Resilience | No erosion or subsidence |
|  | Pressure | Occurrence of them |
| Management groups | Resilience | Presence of management groups about amenities or conveniences |
|  | Pressure | Absence of them |
| Attracting visitors | Resilience | Presence of sports, fishing, or shell-gathering events |
|  | Pressure | Absence of them |
| Publicity work | Resilience | Presence of public relations via the web and distribution of advertisements |
|  | Pressure | Absence of them |
| Incidental facilities | Resilience | Presence of rest huts, public toilets, event rooms, wash facilities |
|  | Pressure | Absence of them |
| Accessibility | Resilience | Presence of public transportation station or parking |
|  | Pressure | Lack of them |
| Protection of species | Resilience | Implementation of protected areas, removal of alien species, and presence of educational activities |
|  | Pressure | Absence of them |
| Diversity of creatures | Resilience | Maximum score of diversity index (*H’*) in the most recent 5 years |
|  | Pressure | Diversity index (*H’*) equal to 0 |
