# Supplementary material for: Method for the quantitative evaluation of ecosystem services in coastal regions
Source: PeerJ. 2019 Jan 14;6:e6234. doi: 10.7717/peerj.6234 (PMC6336092; doi:10.7717/peerj.6234)
Supplement: Supplemental Information 48 [file peerj-07-6234-s048.docx]

| Year | | 2009 | 2010 | 2011 | 2012 | 2013 |
| --- | --- | --- | --- | --- | --- | --- |
| SN | *X*_4_ | 331 | 252 | 86 | 223 | 168 |
|  | *x*_4_ | 1.00 | 0.76 | 0.26 | 0.67 | 0.51 |
| UK | *X*_4_ | － | － | － | － | 210 |
|  | *x*_4_ | － | － | － | － | 0.63 |
| TR | *X*_4_ | － | － | － | － | 0 |
|  | *x*_4_ | － | － | － | － | 0.00 |
| OR | *X*_4_ | － | － | － | － | 120 |
|  | *x*_4_ | － | － | － | － | 0.36 |
