# Supplementary material for: Method for the quantitative evaluation of ecosystem services in coastal regions
Source: PeerJ. 2019 Jan 14;6:e6234. doi: 10.7717/peerj.6234 (PMC6336092; doi:10.7717/peerj.6234)
Supplement: Supplemental Information 49 — Present status (x4), trend score (T4), PR score (PR4), likely near-term future status (x4,F), service score (I4), and sustainability score (S4). [file peerj-07-6234-s049.docx]

| Tidal flat | SN | UK | TR | OR |
| --- | --- | --- | --- | --- |
| *x*_4_ | 0.51 | 0.63 | 0.00 | 0.36 |
| *T*_4_ | –0.54 | 0.00 | 0.00 | 0.00 |
| *PR*_4_ | –0.16 | 0.32 | –0.14 | 0.53 |
| *x*_4,F_ | 0.30 | 0.70 | 0.00 | 0.43 |
| *I*_4_ | 40.3 | 66.8 | 0.0 | 39.4 |
| *S*_4_ | –41% | +11% | – | +17% |
