# Supplementary material for: Method for the quantitative evaluation of ecosystem services in coastal regions
Source: PeerJ. 2019 Jan 14;6:e6234. doi: 10.7717/peerj.6234 (PMC6336092; doi:10.7717/peerj.6234)
Supplement: Supplemental Information 50 [file peerj-07-6234-s050.docx]

| Tidal flat | Key words | | |
| --- | --- | --- | --- |
|  | No. 1 | No. 2 | No. 3 |
| SN | Siosai | pro-environmental seawall | urban wetland/Yokohama |
| UK | Uminokouen | Yokohama/artificial/beach | tidal flat/Yokohama/Tokyo Bay |
| TR | Tama River/tidal flat/Tokyo Bay | Tama River/estuary/Tokyo Bay | Tama/Tokyo Bay |
| OR | Obitsu River/tidal flat/Tokyo Bay | banzu/tidal flat/ Tokyo Bay | Obitsu/Tokyo Bay |
