# Supplementary material for: Method for the quantitative evaluation of ecosystem services in coastal regions
Source: PeerJ. 2019 Jan 14;6:e6234. doi: 10.7717/peerj.6234 (PMC6336092; doi:10.7717/peerj.6234)
Supplement: Supplemental Information 51 [file peerj-07-6234-s051.docx]

| Year | | 2009 | 2010 | 2011 | 2012 | 2013 |
| --- | --- | --- | --- | --- | --- | --- |
| SN | *X*_5_ | 2 | 5 | 4 | 4 | 8 |
|  | *x*_5_ | 0.14 | 0.36 | 0.29 | 0.29 | 0.57 |
| UK | *X*_5_ | 2 | 3 | 3 | 4 | 3 |
|  | *x*_5_ | 0.4 | 0.21 | 0.21 | 0.29 | 0.14 |
| TR | *X*_5_ | 4 | 3 | 6 | 7 | 9 |
|  | *x*_5_ | 0.29 | 0.21 | 0.43 | 0.50 | 0.64 |
| OR | *X*_5_ | 3 | 4 | 4 | 14 | 8 |
|  | *x*_5_ | 0.21 | 0.29 | 0.29 | 1.00  0.57000.0 | 0.57 |
