# Supplementary material for: Method for the quantitative evaluation of ecosystem services in coastal regions
Source: PeerJ. 2019 Jan 14;6:e6234. doi: 10.7717/peerj.6234 (PMC6336092; doi:10.7717/peerj.6234)
Supplement: Supplemental Information 52 — Present status (x5), trend score (T5), PR score (PR5), likely near-term future status (x5,F), service score (I5), and sustainability score (S5). [file peerj-07-6234-s052.docx]

| Tidal flat | SN | UK | TR | OR |
| --- | --- | --- | --- | --- |
| *x*_5_ | 0.57 | 0.14 | 0.64 | 0.57 |
| *T*_5_ | 0.39 | 0.04 | 0.50 | 0.71 |
| *PR*_5_ | 0.00 | 0.00 | 0.00 | 0.00 |
| *x*_5,F_ | 0.72 | 0.15 | 0.86 | 0.84 |
| *I*_5_ | 64.7 | 14.5 | 75.1 | 70.8 |
| *S*_5_ | +26% | +2% | +34% | +48% |
