# Supplementary material for: Method for the quantitative evaluation of ecosystem services in coastal regions
Source: PeerJ. 2019 Jan 14;6:e6234. doi: 10.7717/peerj.6234 (PMC6336092; doi:10.7717/peerj.6234)
Supplement: Supplemental Information 53 [file peerj-07-6234-s053.docx]

| Environmental factor | | Condition of pressure or resilience |
| --- | --- | --- |
| Healthy habitat | Resilience | No occurrence of odor due to blue tide or other organisms, mass death of organisms, outbreak of specific species (*Ulva* sp.) |
|  | Pressure | Occurrence of them |
| Stability of ground | Resilience | No occurrence of erosion, subsidence |
|  | Pressure | Occurrence of them |
| Management of ground condition | Resilience | Implementation of beach nourishment, leveling |
|  | Pressure | Absence of them |
| Management groups | Resilience | Presence of management groups of tidal flat about amenities and conveniences |
|  | Pressure | Absence of them |
| Fishery | Resilience | Fishing activities are active or stable in the surrounding area |
|  | Pressure | Fishermen are not active or stable in the surrounding area |
