# Supplementary material for: Method for the quantitative evaluation of ecosystem services in coastal regions
Source: PeerJ. 2019 Jan 14;6:e6234. doi: 10.7717/peerj.6234 (PMC6336092; doi:10.7717/peerj.6234)
Supplement: Supplemental Information 54 [file peerj-07-6234-s054.docx]

| Site | *X*_6.1_ (times/ y) | *x*_6.1_ | *X*_6.2_ (structures) | *x*_6.2_ | *x*_6_ |
| --- | --- | --- | --- | --- | --- |
| TR | 2 | 1.00 | 0 | 0.00 | 0.50 |
| OR | 1 | 0.50 | 1 | 1.00 | 0.75 |
