# Supplementary material for: Method for the quantitative evaluation of ecosystem services in coastal regions
Source: PeerJ. 2019 Jan 14;6:e6234. doi: 10.7717/peerj.6234 (PMC6336092; doi:10.7717/peerj.6234)
Supplement: Supplemental Information 55 — Present status (x6), trend score (T6), PR score (PR6), likely near-term future status (x6,F), service score (I6), and sustainability score (S6). [file peerj-07-6234-s055.docx]

| Tidal flat | SN | UK | TR | OR |
| --- | --- | --- | --- | --- |
| *x*_6_ | － | － | 0.50 | 0.75 |
| *T*_6_ | － | － | 0.00 | 0.00 |
| *PR*_6_ | － | － | 0.10 | 0.40 |
| *x*_6,F_ | － | － | 0.52 | 0.85 |
| *I*_6_ | － | － | 50.8 | 80.0 |
| *S*_6_ | － | － | +3% | +13% |

Note: SN and UK were excluded because these types of activities and structures are not permitted in these areas.
