# Supplementary material for: Method for the quantitative evaluation of ecosystem services in coastal regions
Source: PeerJ. 2019 Jan 14;6:e6234. doi: 10.7717/peerj.6234 (PMC6336092; doi:10.7717/peerj.6234)
Supplement: Supplemental Information 57 [file peerj-07-6234-s057.docx]

| Site | *V* (people/d) | *X*_7_ | *x*_7_ |
| --- | --- | --- | --- |
| UK | 836 | 522 | 1.00 |
| TR | 17 | 17 | 0.03 |
| OR | 36 | 13 | 0.03 |
