# Supplementary material for: Method for the quantitative evaluation of ecosystem services in coastal regions
Source: PeerJ. 2019 Jan 14;6:e6234. doi: 10.7717/peerj.6234 (PMC6336092; doi:10.7717/peerj.6234)
Supplement: Supplemental Information 58 — Present status (x7), trend score (T7), PR score (PR7), likely near-term future status (x7,F), service score (I7), and sustainability score (S7). [file peerj-07-6234-s058.docx]

| Tidal flat | SN | UK | TR | OR |
| --- | --- | --- | --- | --- |
| *x*_7_ | － | 1.00 | 0.03 | 0.03 |
| *T*_7_ | － | 0.00 | 0.00 | 0.00 |
| *PR*_7_ | － | 0.30 | –0.10 | 0.50 |
| *x*_7,F_ | － | 1.10 | 0.03 | 0.03 |
| *I*_7_ | － | 100 | 3.2 | 2.8 |
| *S*_7_ | － | +10% | –3% | +17% |

Note: SN was excluded because these activities are not permitted in this area.
