# Supplementary material for: Method for the quantitative evaluation of ecosystem services in coastal regions
Source: PeerJ. 2019 Jan 14;6:e6234. doi: 10.7717/peerj.6234 (PMC6336092; doi:10.7717/peerj.6234)
Supplement: Supplemental Information 60 [file peerj-07-6234-s060.docx]

| Year | | 2009 | 2010 | 2011 | 2012 | 2013 |
| --- | --- | --- | --- | --- | --- | --- |
| SN | *X*_8_ | 7.60 | 13.7 | 5.15 | 13.1 | 10.0 |
|  | *x*_8_ | 0.55 | 1.00 | 0.38 | 0.95 | 0.73 |
| UK | *X*_8_ | 3.87 | 4.05 | 4.35 | 3.94 | 3.94 |
|  | *x*_8_ | 0.28 | 0.30 | 0.32 | 0.29 | 0.29 |
| TR | *X*_8_ | 1.55 | 2.34 | 5.96 | 3.55 | 9.59 |
|  | *x*_8_ | 0.11 | 0.17 | 0.43 | 0.26 | 0.70 |
| OR | *X*_8_ | 1.60 | 2.29 | 2.35 | 2.60 | 2.91 |
|  | *x*_8_ | 0.12 | 0.17 | 0.17 | 0.19 | 0.21 |
