# Supplementary material for: Method for the quantitative evaluation of ecosystem services in coastal regions
Source: PeerJ. 2019 Jan 14;6:e6234. doi: 10.7717/peerj.6234 (PMC6336092; doi:10.7717/peerj.6234)
Supplement: Supplemental Information 61 — Present status (x8), trend score (T8), PR score (PR8), likely near-term future status (x8,F), service score (I8), and sustainability score (S8). [file peerj-07-6234-s061.docx]

| Tidal flat | SN | UK | TR | OR |
| --- | --- | --- | --- | --- |
| *x*_8_ | 0.73 | 0.29 | 0.70 | 0.21 |
| *T*_8_ | 0.15 | 0.02 | 0.63 | 0.11 |
| *PR*_8_ | –0.11 | 0.53 | 0.27 | 0.40 |
| *x*_8,F_ | 0.78 | 0.34 | 1.06 | 0.25 |
| *I*_8_ | 75.6 | 31.4 | 87.7 | 23.3 |
| *S*_8_ | +18% | +13% | +9% | +10% |
