# Supplementary material for: Method for the quantitative evaluation of ecosystem services in coastal regions
Source: PeerJ. 2019 Jan 14;6:e6234. doi: 10.7717/peerj.6234 (PMC6336092; doi:10.7717/peerj.6234)
Supplement: Supplemental Information 62 [file peerj-07-6234-s062.docx]

|  | Mollusk | Annelid | Arthropod | Others |
| --- | --- | --- | --- | --- |
| P/B ratio | 1 | 3 | 1 | 2 |
| $\gamma_{s}$ | 0.036 | 0.084 | 0.090 | 0.095 |
