# Supplementary material for: Method for the quantitative evaluation of ecosystem services in coastal regions
Source: PeerJ. 2019 Jan 14;6:e6234. doi: 10.7717/peerj.6234 (PMC6336092; doi:10.7717/peerj.6234)
Supplement: Supplemental Information 64 [file peerj-07-6234-s064.docx]

| Year | | 2009 | 2010 | 2011 | 2012 | 2013 |
| --- | --- | --- | --- | --- | --- | --- |
| SN | *X*_9_ | 68 | 214 | 152 | 83 | 47 |
|  | *x*_9_ | 0.32 | 1.00 | 0.71 | 0.39 | 0.22 |
| UK | *X*_9_ | 1 | - | - | 14 | 9 |
|  | *x*_9_ | 0.00 | - | - | 0.07 | 0.04 |
| TR | *X*_9_ | 11 | 11 | 14 | 17 | 28 |
|  | *x*_9_ | 0.05 | 0.05 | 0.07 | 0.08 | 0.13 |
| OR | *X*_9_ | 2 | 7 | 8 | 4 | 13 |
|  | *x*_9_ | 0.01 | 0.03 | 0.04 | 0.02 | 0.06 |
