# Supplementary material for: Method for the quantitative evaluation of ecosystem services in coastal regions
Source: PeerJ. 2019 Jan 14;6:e6234. doi: 10.7717/peerj.6234 (PMC6336092; doi:10.7717/peerj.6234)
Supplement: Supplemental Information 65 — Present status (x9), trend score (T9), PR score (PR9), likely near-term future status (x9,F), service score (I9), and sustainability score (S9). [file peerj-07-6234-s065.docx]

| Tidal flat | SN | UK | TR | OR |
| --- | --- | --- | --- | --- |
| *x*_9_ | 0.22 | 0.04 | 0.13 | 0.06 |
| *T*_9_ | –0.40 | 0.06 | 0.10 | 0.05 |
| *PR*_9_ | –0.11 | 0.53 | 0.27 | 0.40 |
| *x*_9,F_ | 0.15 | 0.05 | 0.15 | 0.07 |
| *I*_9_ | 18.8 | 4.6 | 14.3 | 6.6 |
| *S*_9_ | –8% | +16% | +54% | +31% |
