# Supplementary material for: Method for the quantitative evaluation of ecosystem services in coastal regions
Source: PeerJ. 2019 Jan 14;6:e6234. doi: 10.7717/peerj.6234 (PMC6336092; doi:10.7717/peerj.6234)
Supplement: Supplemental Information 66 [file peerj-07-6234-s066.docx]

|  | Mollusk | Annelid | Arthropod | Others |
| --- | --- | --- | --- | --- |
| Carbon contents g-C/g-wet | 0.026 | 0.060 | 0.064 | 0.068 |
