# Supplementary material for: Method for the quantitative evaluation of ecosystem services in coastal regions
Source: PeerJ. 2019 Jan 14;6:e6234. doi: 10.7717/peerj.6234 (PMC6336092; doi:10.7717/peerj.6234)
Supplement: Supplemental Information 67 [file peerj-07-6234-s067.docx]

| Environmental factor | | Condition of pressure or resilience |
| --- | --- | --- |
| Anoxic water | Resilience | DO concentration is >4 mg/L |
|  | Pressure | DO concentration is <4 mg/L |
| Blue tide | Resilience | No occurrence of blue tide |
|  | Pressure | Occurrence of blue tide |
| Primary productivity | Resilience | Chl-a concentration is > 3 μg/L |
|  | Pressure | Chl-a concentration is < 3 μg/L |
| Stability of ground | Resilience | No occurrence erosion, floating sand, subsidence |
|  | Pressure | Occurrence of them |
| Source of juveniles | Resilience | Existence of a nearby tidal flat |
|  | Pressure | No nearby tidal flat |
| Management of ground condition | Resilience | Implementation of sand capping, cultivation, removal of *Ulva* sp. |
|  | Pressure | Absence of them |
