# Supplementary material for: Method for the quantitative evaluation of ecosystem services in coastal regions
Source: PeerJ. 2019 Jan 14;6:e6234. doi: 10.7717/peerj.6234 (PMC6336092; doi:10.7717/peerj.6234)
Supplement: Supplemental Information 68 [file peerj-07-6234-s068.docx]

| Environmental factor | | Condition of pressure or resilience |
| --- | --- | --- |
| Stability of ground | Resilience | No occurrence of erosion, floating sand |
|  | Pressure | Occurrence of erosion, floating sand |
| Organic load | Resilience | Highest Chl-a concentration in the most recent 5 years |
|  | Pressure | Chl-a concentration equal to 0 |
| Death of organisms | Resilience | Occurrence of mass death of organisms by hypoxia or blue tide |
|  | Pressure | Lack of them |
| Embedding organic matter | Resilience | Implementation of sand capping |
|  | Pressure | Implementation of cultivation, dredging |
