# Supplementary material for: Method for the quantitative evaluation of ecosystem services in coastal regions
Source: PeerJ. 2019 Jan 14;6:e6234. doi: 10.7717/peerj.6234 (PMC6336092; doi:10.7717/peerj.6234)
Supplement: Supplemental Information 69 [file peerj-07-6234-s069.docx]

| Year | | 2009 | 2010 | 2011 | 2012 | 2013 |
| --- | --- | --- | --- | --- | --- | --- |
| SN | *X*_10_ | 661 | 1060 | 464 | 494 | 608 |
|  | *x*_10_ | 0.62 | 0.94 | 0.43 | 0.46 | 0.57 |
| UK | *X*_10_ | - | - | - | 190 | 180 |
|  | *x*_10_ | - | - | - | 0.18 | 0.17 |
| TR | *X*_10_ | 1070 | 1012 | 1044 | 874 | 890 |
|  | *x*_10_ | 1.00 | 0.95 | 0.98 | 0.82 | 0.83 |
| OR | *X*_10_ | 136 | 139 | 141 | 137 | 144 |
|  | *x*_10_ | 0.13 | 0.13 | 0.13 | 0.13 | 0.13 |
