# Supplementary material for: Method for the quantitative evaluation of ecosystem services in coastal regions
Source: PeerJ. 2019 Jan 14;6:e6234. doi: 10.7717/peerj.6234 (PMC6336092; doi:10.7717/peerj.6234)
Supplement: Supplemental Information 70 — Present status (x10), trend score (T10), PR score (PR10), likely near-term future status (x10,F), service score (I10), and sustainability score (S10). [file peerj-07-6234-s070.docx]

| Tidal flat | SN | UK | TR | OR |
| --- | --- | --- | --- | --- |
| *x*_10_ | 0.57 | 0.17 | 0.83 | 0.13 |
| *T*_10_ | –0.29 | –0.05 | –0.23 | 0.01 |
| *PR*_10_ | 0.18 | 0.02 | 0.37 | 0.10 |
| *x*_10,F_ | 0.49 | 0.16 | 0.80 | 0.14 |
| *I*_10_ | 53.0 | 16.6 | 81.8 | 13.7 |
| *S*_10_ | –5% | –1% | –7% | +1% |
