# Supplementary material for: Method for the quantitative evaluation of ecosystem services in coastal regions
Source: PeerJ. 2019 Jan 14;6:e6234. doi: 10.7717/peerj.6234 (PMC6336092; doi:10.7717/peerj.6234)
Supplement: Supplemental Information 71 [file peerj-07-6234-s071.docx]

| Environmental factor | | Condition of pressure or resilience |
| --- | --- | --- |
| Alien species | Resilience | No alien species |
|  | Pressure | Existence of alien species |
| Healthy habitat | Resilience | No occurrence of mass death of organisms, outbreak of specific species (*Ulva* sp.) |
|  | Pressure | Occurrence of them |
| Stability of ground | Resilience | No occurrence of erosion, floating sand, subsidence |
|  | Pressure | Occurrence of them |
| Source of juveniles | Resilience | Existence of a nearby tidal flat |
|  | Pressure | No nearby tidal flat |
| Protection of species | Resilience | Existence of removal of alien species, protected areas |
|  | Pressure | No removal alien species or protected areas |
| Diversity of environment | Pressure | Presence of countermeasures to diversify the ground environment |
|  | Resilience | Absence of them |
