# Supplementary material for: Method for the quantitative evaluation of ecosystem services in coastal regions
Source: PeerJ. 2019 Jan 14;6:e6234. doi: 10.7717/peerj.6234 (PMC6336092; doi:10.7717/peerj.6234)
Supplement: Supplemental Information 72 [file peerj-07-6234-s072.docx]

| Year | | 2009 | 2010 | 2011 | 2012 | 2013 |
| --- | --- | --- | --- | --- | --- | --- |
| SN | *X*_11_ | 2.29 | - | 2.75 | 2.95 | 2.86 |
|  | *x*_11_ | 0.66 | - | 0.79 | 0.85 | 0.83 |
| UK | *X*_11_ | 2.12 | - | - | 3.00 | 3.13 |
|  | *x*_11_ | 0.61 | - | - | 0.87 | 0.90 |
| TR | *X*_11_ | 3.21 | 3.41 | 2.91 | 3.15 | 3.08 |
|  | *x*_11_ | 0.93 | 0.98 | 0.84 | 0.91 | 0.89 |
| OR | *X*_11_ | 3.31 | 2.89 | 3.00 | 3.46 | 2.68 |
|  | *x*_11_ | 0.95 | 0.84 | 0.86 | 1.00 | 0.77 |
