# Supplementary material for: Method for the quantitative evaluation of ecosystem services in coastal regions
Source: PeerJ. 2019 Jan 14;6:e6234. doi: 10.7717/peerj.6234 (PMC6336092; doi:10.7717/peerj.6234)
Supplement: Supplemental Information 73 [file peerj-07-6234-s073.docx]

| Categories | State | Weighting factor ($\alpha_{i}$) |
| --- | --- | --- |
| CR | The risk of extinction in the wild in the very near future | 0.8 |
| EN | The risk of extinction in the wild in the near future  (not as high as CR) | 0.8 |
| VU | The risk of extinction is increasing | 0.8 |
| NT | The viable basis is vulnerable | 0.4 |
| DD | Information is missing | 0.2 |
| LP | Populations isolated locally and high risk of extinction | 0.4 |
