# Supplementary material for: Method for the quantitative evaluation of ecosystem services in coastal regions
Source: PeerJ. 2019 Jan 14;6:e6234. doi: 10.7717/peerj.6234 (PMC6336092; doi:10.7717/peerj.6234)
Supplement: Supplemental Information 75 [file peerj-07-6234-s075.docx]

| Year | | 2009 | 2010 | 2011 | 2012 | 2013 |
| --- | --- | --- | --- | --- | --- | --- |
| SN | *X*_12_ | 0.40 | 0.00 | 0.24 | 0.50 | 0.10 |
|  | *x*_12_ | 0.33 | 0.00 | 0.20 | 0.42 | 0.08 |
| UK | *X*_12_ | 0.00 | - | - | 0.40 | 0.13 |
|  | *x*_12_ | 0.00 | - | - | 0.33 | 0.11 |
| TR | *X*_12_ | 0.20 | 0.87 | 0.95 | 0.92 | 1.13 |
|  | *x*_12_ | 0.17 | 0.72 | 0.79 | 0.77 | 0.94 |
| OR | *X*_12_ | 1.20 | 0.40 | 0.40 | 1.20 | 1.20 |
|  | *x*_12_ | 1.00 | 0.33 | 0.33 | 1.00 | 1.00 |
