# Supplementary material for: Method for the quantitative evaluation of ecosystem services in coastal regions
Source: PeerJ. 2019 Jan 14;6:e6234. doi: 10.7717/peerj.6234 (PMC6336092; doi:10.7717/peerj.6234)
Supplement: Supplemental Information 76 — Present status (x12), trend score (T12), PR score (PR12), likely near-term future status (x12,F), service score (I12), and sustainability score (S12). [file peerj-07-6234-s076.docx]

| Tidal flat | SN | UK | TR | OR |
| --- | --- | --- | --- | --- |
| *x*_12_ | 0.08 | 0.11 | 0.94 | 1.00 |
| *T*_12_ | –0.04 | 0.24 | 0.80 | 0.33 |
| PR_12_ | –0.33 | 0.00 | 0.00 | 0.25 |
| *x*_12,F_ | 0.07 | 0.13 | 1.45 | 1.31 |
| *I*_12_ | 7.8 | 12.0 | 100 | 100 |
| *S*_12_ | +10% | +28% | –2% | +2% |
