# Supplementary material for: Method for the quantitative evaluation of ecosystem services in coastal regions
Source: PeerJ. 2019 Jan 14;6:e6234. doi: 10.7717/peerj.6234 (PMC6336092; doi:10.7717/peerj.6234)
Supplement: Supplemental Information 77 — Present status (x11), trend score (T11), PR score (PR11), likely near-term future status (x11,F), service score (I11), and sustainability score (S11). [file peerj-07-6234-s077.docx]

| Tidal flat | SN | UK | TR | OR |
| --- | --- | --- | --- | --- |
| *x*_11_ | 0.83 | 0.90 | 0.89 | 0.77 |
| *T*_11_ | 0.23 | 0.38 | –0.07 | –0.10 |
| *PR*_11_ | –0.17 | 0.08 | 0.08 | 0.25 |
| *x*_11,F_ | 0.90 | 1.16 | 0.87 | 0.79 |
| *I*_11_ | 86.5 | 100 | 87.9 | 78.1 |
| *S*_11_ | +10% | +28% | –2% | +2% |
